# Place and behavioral modulation of hippocampal neurons during immobility

**DOI:** 10.1038/s41467-026-75492-w

**Published:** 2026-07-24

**Authors:** Nicola Sartorato, Ioannis S. Zouridis, Ulzii-Utas Narantsatsralt, Eduardo Blanco-Hernández, Andrea Burgalossi

**Affiliations:** 1 Institute for Neurobiology, Eberhard Karls University of Tübingen, Tübingen, Germany; 2 Werner Reichardt Centre for Integrative Neuroscience, Tübingen, Germany; 3 Graduate Training Centre of Neuroscience, International Max Planck Research School (IMPRS), Tübingen, Germany; 4 Max Planck Institute for Biological Cybernetics, Tübingen, Germany; 5 Institute of Experimental Epileptology and Cognition Research, University Hospital Bonn, Bonn, Germany

**Keywords:** Hippocampus, Neural circuits

## Abstract

Behavioral state fluctuations profoundly impact episodic memory processing. To explore the underlying mechanisms, we recorded CA1 place cells in head-fixed male mice and focused on awake immobility to capture spontaneous behavioral state fluctuations by facial motion, pupillometry, and local field potential (LFP) analysis. We found that during awake immobility, the duration of spontaneous whisker-pad motion events correlated with ongoing levels of arousal and modulated both the frequency and the power of theta oscillations. CA1 place cells continued to encode location during immobility, with the spatial code being primarily driven by a subset of behaviorally-modulated place cells which increased their firing upon behavioral state transitions. Single-cell stimulation during immobility was sufficient for the induction of place fields, indicating that plasticity mechanisms can be engaged even in the absence of locomotion. Altogether, these data indicate that behavioral state fluctuations might contribute to episodic memory processing by modulating theta oscillatory dynamics and hippocampal gain via the engagement of a discrete place-cell ensemble.

## Abstract (web summary)

Neural mechanisms underlying episodic memory processing are not fully understood. Here authors show that arousal fluctuations modulate CA1 theta dynamics and recruit a subset of hippocampal place cells that continue to encode space during awake immobility. Single-cell stimulation induced novel place fields, revealing place-field plasticity independent of locomotion.

## Introduction

Place cells are a striking neural correlate of hippocampal memory. These neurons encode the animal’s position in an environment^1^ and, more generally, events of episodic experience^2–4^ and have been causally linked to memory functions^5–7^. Given their importance for episodic memory, the phenomenology of the place representation, as well as its dynamics and plasticity during memory processing, has been extensively investigated^8,9^. Since place cells exhibit their characteristic spatial firing pattern during exploratory behavior (i.e., during locomotion), recordings in freely moving animals have become the standard approach for recording place cells. As a result, awake immobility periods have been largely excluded from place cell analysis. However, awake immobility is an ethologically relevant behavioral state, especially important for episodic memory. Numerous exploratory behaviors linked to episodic learning occur in stationary animals (i.e., at a fixed spatial location), such as interactions with objects or conspecifics, rearing, and head-scanning behavior^10–12^. So far, only a few studies have focused on place cell dynamics during immobility periods (e.g., refs. ^13–15^) with a recent study^13^ showing that unique hippocampal processes are indeed engaged in specific hippocampal subfields (e.g., the CA2 region). Place cell plasticity mechanisms have also been almost exclusively studied in locomoting animals^16–20^. Active locomotion is known to engage spatial entorhinal inputs onto CA1 pyramidal neurons, and rapid plasticity mechanisms at these distal inputs are thought to support place field formation^17,20^. Recent behavioral and electrophysiological studies have shown that new place fields can also emerge in correspondence with awake immobility-related behaviors (e.g., head scanning^10^, rearing^11^); however, the underlying plasticity mechanisms have remained unresolved.

During active locomotion, the rodent hippocampus displays prominent local field potential (LFP) oscillations in the theta frequency range (6–10 Hz). Theta oscillations—although not necessary for the emergence of the place code^21,22^—play a major role in organizing place cell activity into temporal sequences that map the available space (and, similarly, episodic events^4,23,24^). In contrast to the large body of knowledge on theta oscillations during locomotion, comparatively little is known about immobility-related theta^25–28^. Although earlier work indicated that LFP theta bouts can be evoked by emotionally salient stimuli^26–28^ and being associated with specific body movements^29^, the behavioral correlates of these theta oscillations have remained largely unresolved. At present, it is not known whether and how hippocampal oscillatory activity is modulated by behavioral state fluctuations, and whether place cell activity is temporally organized during immobility-related theta oscillations.

Fluctuating levels of arousal play a fundamental role in episodic memory processes. For example, emotionally salient events, which engage the arousal system, may facilitate the encoding and drive enhanced consolidation of the corresponding memory traces (e.g., refs. ^30,31^). These mechanisms are thought to serve a fundamental ethological purpose in that they ensure that salient experiences are optimally encoded, stored, and retrieved for flexibly guiding behavior, while uninformative experiences are forgotten. Yet, despite the importance of arousal-mediated mechanisms in episodic memory processing, which is well documented in several species, the underlying hippocampal mechanisms have remained largely unexplored.

Exploring whether and how hippocampal place cells are modulated by arousal has remained methodologically challenging. This is because locomotion—where place cell activity has been classically characterized—is a homogeneous behavioral state, tightly coupled to arousal^32–35^. Indeed, the onset of locomotion, as well as vestibular activation, are correlated with pupil dilations and brain state transitions^32,34,36^. Hence, during locomotion, the contribution of behavioral state fluctuations to hippocampal dynamics cannot be easily dissociated from the effect of self-motion.

To overcome these limitations, we monitored CA1 place cell activity in head-fixed mice while they were running on a linear track, as well as during awake immobility at random locations along the track. This “space clamping” approach provided us with the opportunity to study the effect of spontaneous behavioral state fluctuations on hippocampal dynamics, in the absence of active locomotion and vestibular activation.

## Results

### CA1 place cell recordings and behavioral analysis during running and immobility

**Fig. 1:**
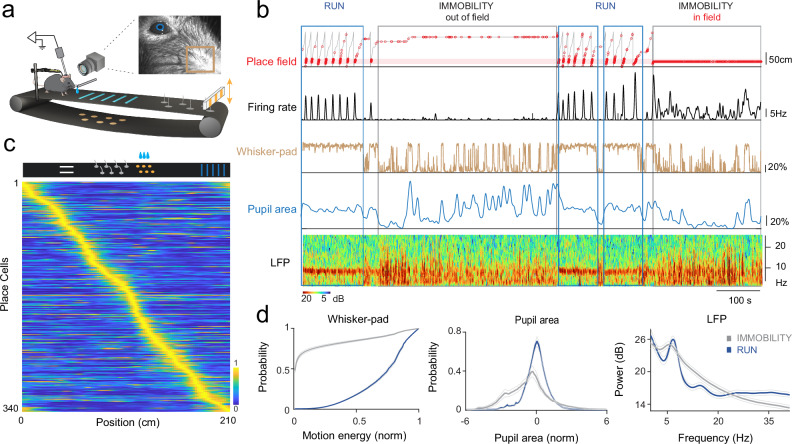
Place cell recordings and behavioral state changes during running and immobility. **a** Schematic showing combined videography and silicon probe recordings in the CA1 region of the hippocampus from a head-fixed mouse on a linear treadmill. The treadmill brake (bar icon) was used to impose immobility at random track locations. The inset shows an example video frame of the mouse face, and the corresponding regions of interest for behavioral analysis: pupil (blue), whisker-pad (light brown) (see Methods). **b** Representative place-cell recording and behavioral state changes during running and awake immobility. From top to bottom: spike-trajectory plot for a representative place cell (red dots indicate spikes, the place field is indicated as a red shadow, and black rectangles indicate two immobility epochs, one inside and one outside the place field); instantaneous firing rate (black); whisker-pad motion energy (light brown); pupil area (blue); LFP time-frequency spectrogram from a representative channel in the hippocampus. **c** Normalized ratemaps of the recorded place cells (*n* = 340 cells). On top, schematic depicting the sensory cues and the reward along the treadmill belt. **d** Behavioral metrics and hippocampal LFP during running (blue) and immobility (gray) (*n* = 34 sessions, *n* = 5 mice). Left, whisker-pad motion energy; middle, normalized pupil area; right, LFP power spectra. For all three metrics and all 34 sessions, *p* < 0.001 after Bonferroni correction, two-sided Kolmogorov–Smirnov test. Bonferroni-corrected *α* = 1.47e-3. Lines indicate means; error bars indicate SEM. Source data for this figure are provided as a Source Data file.

To record hippocampal place cells during periods of running and immobility, we trained head-fixed mice to run for water rewards on a 2.1 m-long linear track with visuo-tactile sensory cues (Fig. 1a; refs. ^37,38^). Then, we performed silicon-probe recordings in the dorsal CA1, while mice alternated between periods of active running and experimenter-imposed immobility, achieved by activating a treadmill brake at random locations along the track (Fig. 1a–c and Supplementary Fig. 1). To monitor the animal’s behavioral state, we combined single unit recordings with pupillometry, whisker-pad motion, and LFP analysis (Fig. 1a, b). A representative recording from a CA1 place cell is shown in Fig. 1b. During the running periods, this cell fired in a specific location of the belt and was classified as a place cell (see Methods). After running, the activity of the cell was also monitored while the animal was kept stationary within and outside the place field (Fig. 1b).

Altogether, we recorded the activity of 520 CA1 neurons from 5 mice. Consistent with previous work^37–39^, running along the treadmill reliably engaged the hippocampal place representation, with the large majority of putative principal neurons (340 out of 520) being classified as place cells (see Methods). Place fields uniformly tiled the available space (Fig. 1c), with a slight over-representation of the reward’s position and of prominent tactile cues on the belt (see also refs. ^39–41^).

In line with previous work in head-fixed and freely-moving animals, running was accompanied by sustained LFP theta oscillations^42^ (Fig. 1b, d). During immobility, the brain state fluctuated over time, corresponding to changes in LFP spectral characteristics (Fig. 1b, d). Pupil and whisker-pad motion dynamics were also remarkably different. While during locomotion, animals engaged in whisker-pad motion and the pupil area remained rather constant, during immobility, the behavioral state was more heterogeneous, characterized by intermittent periods of spontaneous whisker-pad motion and larger pupil fluctuations (Fig. 1b, d). These data indicate that during running, the behavioral state is more homogeneous (and likely corresponding to a high-arousal state^34–36^) compared to immobility, where the internal state is more variable and spontaneously fluctuating.

### Hippocampal place coding during awake immobility

**Fig. 2:**
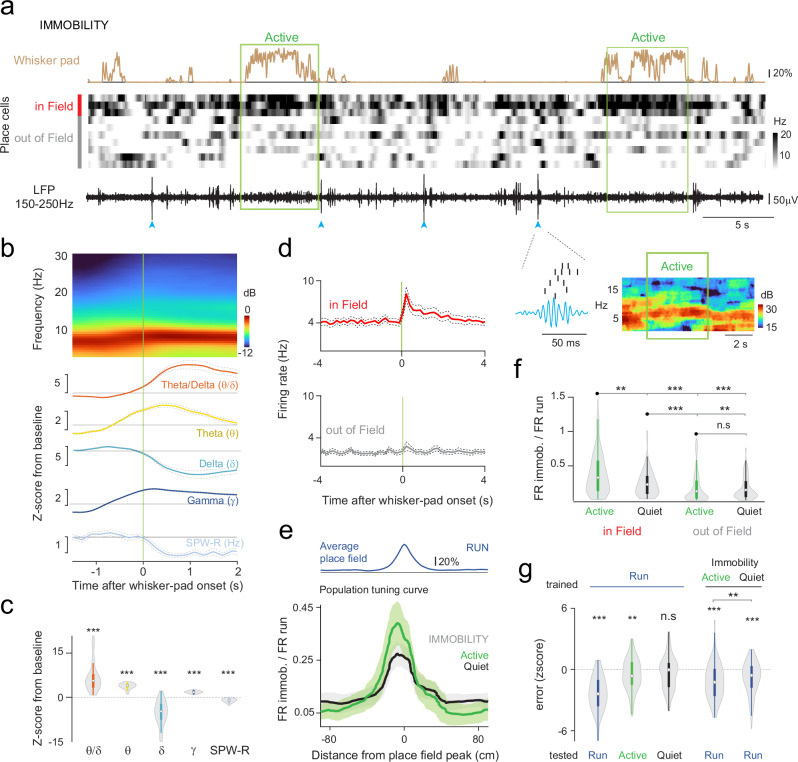
CA1 place coding during awake immobility. **a** Awake immobility epoch showing place cell activity and brain state transitions associated with whisker-pad motion (active immobility, green). From top to bottom: whisker-pad motion energy (light brown); Gaussian-smoothed heatmap of place cell activities, with place fields located either within (red) or outside (gray) the current immobility position; band-pass filtered LFP trace showing hippocampal ripples (gray). Insets show a representative ripple (left) and time-resolved spectrogram of the first active period (right). **b** Perievent time changes of brain state at the onset of whisker-pad motion. From top to bottom: average event-triggered spectrogram of hippocampal LFP; theta/delta ratio (orange); theta power (yellow); delta power (cyan); gamma power (dark blue); sharp-wave ripple rate (light blue). *n* = 32 sessions, *n* = 30 sessions for the SPW-R rate. Traces were z-scored to pre-event periods. Lines indicate means; error bars indicate SEM. **c** Z-scored changes in theta/delta ratio and spectral power for theta (6–10 Hz), delta (0.5–4 Hz), gamma (70–90 Hz) power, and SPW-R rate upon transition to active immobility. Two-sided Wilcoxon signed-rank test against baseline (from −2 to 0 s; theta-delta, *p* = 7.95e-7; delta, *p* = 2.04e-6; theta, *p* = 2.69e-6; gamma, *p* = 7.95e-7; SPW-R, *p* = 2.56e-6; Bonferroni corrected *α* = 0.01). *** *p* < 0.001, same *n* as in (**b**). Violins show probability density; inner boxplots show median (white dot), Interquartile Range (IQR, solid box), and 1.5× IQR (whiskers). **d** Peristimulus time histogram showing place cell firing rates in the field (top) and out of the field (bottom) upon transitions to active immobility (per-session averages, *n* = 26 sessions with “in field” cells and *n* = 27 with “out field” cells). Mean ± SEM are shown. **e** Population average tuning curves showing place cell firing (normalized to in-field rates during running) as a function of distance from the field during active (green) and quiet immobility (black). Trend lines computed over *n* = 280 immobility periods; *n* = 131 place cells. Top panel: average place field during running (blue). Mean ± SEM are shown. **f** Place-cell firing rates during active and quiet immobility in the field (“in Field” cells, *n* = 166, red) or out of field (“out of Field” cells, *n* = 164 cells, gray). Color codes as in (**e**). Kruskal–Wallis test, *p* = 4.23e-19. Post-hoc tests: active vs quiet (in field, *p* = 1.60e-3; out of field, *p* = 0.74); in field vs out of field (active, *p* = 9.41e-17; quiet, *p* *=* 8.49e-4). *** *p* < 0.001, ** *p* < 0.01 (dots indicate the group being compared to the others). Violins show probability density; inner boxplots show median (white dot), IQR (solid box), and 1.5× IQR (whiskers). **g** Bayesian decoding errors with different combinations of training-testing behavioral windows (indicated in labels, *n* = 88 immobility periods, see also Supplementary Fig. 2). Errors were z-scored with respect to a null distribution. Trained run, tested run, *p* = 1.27e-15; trained run, tested active immobility, *p* = 1.92e-3; trained run, tested quiet immobility, *p* = 0.037; trained active immobility, tested run, *p* = 1.86e-8; trained quiet immobility, tested run, *p* = 3.10e-05; Bonferroni-corrected *α* = 0.01, one-tailed Wilcoxon signed-rank test. Active vs quiet immobility encoding models, *p* = 4.32e-3, Wilcoxon signed-rank test. Violins show probability density; inner boxplots show median (white dot), IQR (solid box), and 1.5× IQR (whiskers). ** *p* < 0.01, *** *p* < 0.001. Source data for this figure are provided as a Source Data file.

We used whisker-pad motion to segment behavioral states into “active” and “quiet” immobility (Fig. 2a and Supplementary Fig. 1; see Methods). Indeed, in line with previous work^43,44^, the onset of whisker-pad motion was a reliable correlate of brain state transitions. The “active” state was associated with increased LFP theta (6–10 Hz) and gamma power (70–90 Hz), alongside a reduction in delta power (0.5–4 Hz) (Fig. 2b, c). Sharp-wave ripples predominantly occurred during “quiet” immobility (~ 70% reduction in ripple rate from quiet immobility: 0.18 ± 0.16 Hz to active immobility: 0.05 ± 0.10 Hz, *p* = 2.60 × 10^−6^, Wilcoxon signed-rank test; Fig. 2b, c), further supporting the binary brain state classification.

The dynamic behavioral state during immobility (Figs. 1b, d and 2a) provided us with the opportunity to test whether place cell activity is modulated by the animal’s behavioral state. To this end, we analyzed all immobility periods inside and outside the cells’ place fields (*n* = 166 “in field” place cells; *n* = 164 “out of field” cells; see Methods). We found that when animals were kept stationary within the cell’s receptive field, transitions to the active state were accompanied by a ~1.7-fold increase in place cell firing (firing rates pre- and post- whisker-pad motion; pre-, 4.04 ± 4.12 Hz; post-, 6.50 ± 6.90 Hz; *p* = 2.00 × 10^−3^, Wilcoxon signed-rank test; Fig. 2d). This increase in firing rates was not observed outside of the place fields (Fig. 2d) indicating that behavioral modulation is gated by spatial location.

The early observations of disrupted place cell firing in restrained rats^25^ led to the assumption that locomotion is necessary for the expression of the hippocampal place representation. To explore whether CA1 spatial modulation was preserved during periods of immobility, we first qualitatively examined spatial tuning during immobility. To this end, we computed population tuning curves during active and quiet states by averaging firing activity across all immobility periods from the recorded cells (since individual tuning curves could not be computed because not all belt locations were systematically sampled during immobility within each session; see Methods). Despite the reduced firing rates compared to running (in-field firing rates; running, 12.90 ± 5.68 Hz; awake immobility, 3.82 ± 3.78 Hz; *n* = 166 place cells; *p* = 5.94 × 10⁻^29^; Wilcoxon signed-rank test) the characteristic bell-shaped profile was retained during immobility, with maximal activity in the “active” state (Fig. 2e)—consistent with the observed behavioral modulation (Fig. 2d). Consistent with these observations, by comparing firing rates inside and outside the cells’ place fields (*n* = 166 “in field” place cells; *n* = 164 “out of field” cells; see Methods), we found that on average, place cells fired at higher rates when animals were kept stationary within the cell’s place field, as compared to outside, irrespective of the behavioral state (firing rates normalized to run periods; active immobility: in field, 0.38 ± 0.29, out of field, 0.18 ± 0.21, post-hoc comparison *p* = 1.02 × 10^−^^16^; quiet immobility: in field, 0.24 ± 0.17, out of field, 0.18 ± 0.16, post-hoc comparison *p* = 8.67 × 10^−^^4^; *p* = 4.23 × 10^−^^19^, Kruskal–Wallis test; Fig. 2f and Supplementary Fig. 2). To directly test whether CA1 place cells still represented the animal’s spatial location during immobility, we used a Bayesian population decoding approach, reconstructing spatial locations using encoding models trained on periods of running, active or quiet immobility (for details, see Methods and Supplementary Fig. 2). We found that, using the “running” model, spatial locations could be decoded during active immobility but not during quiet immobility (active: *p* = 1.93 × 10^−^^3^, quiet: *p* = 3.70 × 10^−^^2^; one-tailed Wilcoxon signed-rank test, Bonferroni-corrected *α* = 0.01; Fig. 2g and Supplementary Fig. 2). Consistently, decoding running locations with the “active immobility“ model yielded lower errors than with the “quiet immobility” model (active: *p* = 1.87 × 10^−^^8^, quiet: *p* = 3.10 × 10^−^^5^, one-tailed Wilcoxon signed-rank test, Bonferroni-corrected *α* = 0.01; Fig. 2g and Supplementary Fig. 2). Neuronal reactivations during sharp wave ripples were also slightly biased by the animal’s location, with a higher tendency of firing within the place field as compared to outside (Supplementary Fig. 3; see also refs. ^45,46^). This effect was, however, abolished by downsampling of spike rates (Supplementary Fig. 3), suggesting that it was driven by higher in-field firing. Decoded spatial locations during sharp-wave ripple–related reactivations clustered around the reward zone (Supplementary Fig. 3; see also refs. ^47,48^), indicating preferential reactivation of this behaviorally salient area. Altogether, these data indicate that during awake immobility, the hippocampal place code is reinstated and dynamically modulated by the behavioral state.

**Fig. 3:**
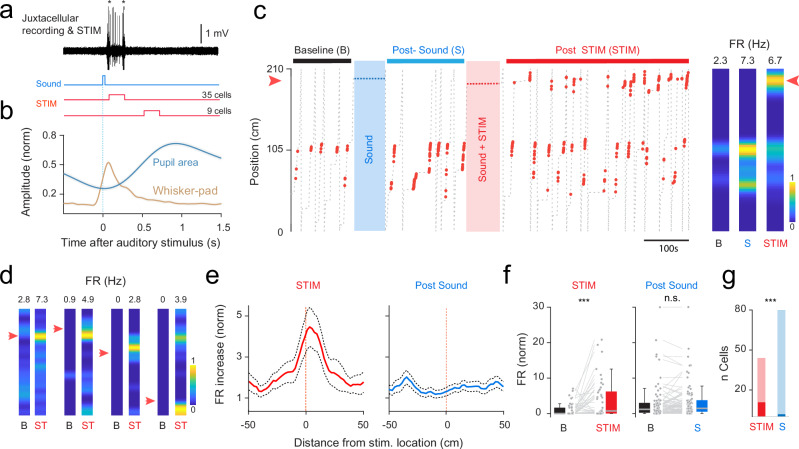
Place field induction by single-cell stimulation during immobility. **a** Representative high-passed filtered juxtacellular trace (top, black) showing a spike train evoked by current injection (bottom, red) during immobility. Stimulation artifacts (asterisks) have been truncated for display purposes. Bottom, time course of the experimental protocol indicating the delivery of the sound stimulus (blue) and the current pulses (red) delivered at two different times in two independent subsets of cells (*n* cells is indicated). **b** Perievent average of whisker-pad motion (light brown) and pupil area (dark blue) during the protocol shown in (**a**). Note that the sound stimulus evoked an active immobility state (see the associated increase in whisker-pad motion and pupil dilation). Current pulses were delivered within the window of maximal whisker-pad motion or pupil dilation. Place field induction was observed with both protocols; hence, data were pooled together. Lines indicate means, shadows indicate SEM (*n* = 170 events). **c** Representative spike trajectory (left) and normalized ratemaps (right) for a CA1 neuron during sequential baseline recording (B), “sound-only” stimulation (S), and “juxtacellular + sound” stimulation (STIM) (same protocol as in (**a**, **b**)). The red arrow indicates the stimulation location. Ratemaps were normalized to the maximal firing rate within each cell (colormaps show normalized values). Max firing rates (Hz) are indicated. **d** Pairs of normalized ratemaps from additional representative recordings, computed before (left, baseline, B) and after (right, STIM, ST) single-cell stimulation during immobility (as in **c**). Note the emergence of a place field and place-field remapping at the stimulus locations (red arrows). Normalization was conducted as in (**c**). Max firing rates (Hz) are indicated. **e** Average firing rate increase centered on the “juxtacellular + sound” stimulation location (left, *n* = 44 cells) and on the “sound-only” stimulation location (right, *n* = 80 cells). Note the increased firing rate around the stimulation location in the juxtacellular stimulation dataset, which was not observed in the sound-only stimulation control dataset. Mean ± SEM is shown. **f** Firing rate comparison before-after condition around the stimulation location for “juxtacellular + sound” stimulated cells (*n* = 44 cells, *p* = 4.90 × 10⁻⁴, two-sided Wilcoxon signed-rank test) and “sound-only” control cells (*n* = 80 cells, *p* = 0.13, Wilcoxon signed-rank test). Boxplots show median (grey line), IQR (solid box), and 1.5× IQR (whiskers). **g** Quantification of the proportion of cells classified as responsive (dark color) and non-responsive (light color) following the “sound + juxtacellular” stimulation condition (STIM, 11 responsive out of 44 cells) versus the “only-sound” stimulation condition (S, 2 responsive out of 80 cells). *p* = 1.89 × 10⁻⁴, two-sided Fisher exact test. Source data for this figure are provided as a Source Data file.

Recent work has demonstrated that during active locomotion, evoking spiking activity in single hippocampal neurons can be sufficient for inducing de novo place fields^16,17,20^. To explore whether similar place-field induction mechanisms might take place during active immobility, we performed juxtacellular recording and stimulation of single CA1 neurons while animals were kept stationary at a random location along the track (Fig. 3a–c). To time juxtacellular stimulation during active immobility, a click-like sound (~ 65–80 dB, 30 ms^49^) was used to reliably evoke an active immobility state prior to single-cell stimulation (Fig. 3b). We found that evoking spikes during active immobility was sufficient to generate a de novo place field at the stimulus location in ~25% (11 out of 44) of the stimulated cells (Fig. 3c, d), with sound stimulation alone not being accountable for this effect (~ 2.5%; 2 of 80 cells, *p* = 1.89 × 10^−4^, Fisher exact test; Fig. 3e–g). This evidence, albeit based on a relatively small number of observations, indicates that place field plasticity mechanisms can be engaged even in the absence of locomotion.

### Theta oscillations during active immobility

**Fig. 4:**
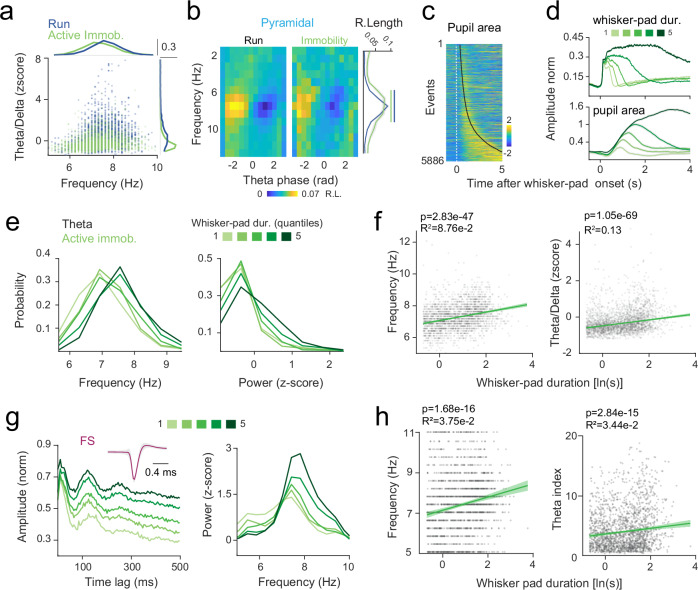
Behavioral modulation of LFP theta oscillations during awake immobility. **a** Theta/delta ratios and theta frequencies of detected theta epochs during active immobility (green; *n* = 2269, 5–10 Hz range) and running (blue; *n* = 2203, 5–10 Hz range, see Methods). Histogram distributions are shown on the side of the scatter plot. **b** Theta-phase analysis of spikes from putative pyramidal cells during running and theta epochs during immobility. Left, color-coded distribution of population phase locking as a function of theta frequency for run periods; middle, same analysis for theta periods during immobility; right panel, quantification of theta-frequency modulation (Raleigh vector length, R.L., mean values ± SEM) for run (blue) and immobility (green). *n* = 32 sessions. Color bars indicate significant regions shared across 75% of the sessions (*p* < 0.001, two-sided Rayleigh test, Bonferroni corrected). **c** Pupil area aligned to the onset of whisker-pad motion (*n* = 5886 events). Events are sorted by whisker-pad duration (indicated by a black line). Color ranges from −2 to +2 z-score. **d** Average time course of whisker-pad motion (top) and pupil area (bottom). Whisker-pad motion events (*n* = 5886 events) were grouped into five quantiles, based on their duration. Quantile groups are represented using a gradient of green. Mean ± SEM is shown. **e** Distributions of peak theta frequency (left) and theta/delta ratio (right) for detected theta epochs associated with whisker-pad motion events (*n* = 2277; see Methods). The data are separated by quantiles of whisker-pad motion duration as in (**d**). **f** Positive linear relationship between whisker-pad motion duration (ln transformed) and peak theta frequency (left, *R*^2^ = 8.76e-2, *F*_(2277,2275)_ = 218, two-sided *t*-test, *p* = 2.83e-47) and theta/delta ratio (right, *R*^2^ = 0.13, *F*_(2277,2275)_ = 334, two-sided *t*-test, *p* = 1.05e-69). Same data as in (**e**). Solid lines represent the best-fit linear regression; shaded areas indicate the 95% confidence intervals. No correction for multiple comparisons was applied. **g** Left, normalized population autocorrelograms of putative FS interneurons (“FS”, purple) during detected theta epochs (*n* = 1779 events, see Methods). The inset shows the average spike waveform ( ± SD) for fast-spiking interneurons. Right, power spectrum computed for the population autocorregrams shown on the left. The data are separated in five quantiles of whisker-pad motion duration (*n* = 1779 events). Color codes as in (e). **h** Positive linear relationship between whisker-pad motion duration (ln transformed) and theta-rhythmicity of FS spike autocorrelograms. Left, peak theta frequency (*R*^2^ = 3.75 × 10^−2^, *F*_(1779,1777)_ = 69.3, two-sided *t*-test, *p* = 1.68 × 10^−16^); right, Theta Index (*R*^2^ = 3.45 × 10^−2^, *F*_(1779,1777)_ = 63.5, two-sided *t*-test, *p* = 2.85 × 10^−15^). Solid lines represent the best-fit linear regression; shaded areas indicate the 95% confidence intervals. No correction for multiple comparisons was applied. Source data for this figure are provided as a Source Data file.

Since transitions to active immobility were associated with increases in theta power and theta/delta ratio (Fig. 2b, c), we sought to systematically characterize theta oscillatory dynamics during this state. To this end, theta epochs were identified via segmentation of time-resolved LFP spectrograms, based on standard criteria on theta/delta ratios^50–52^(theta band [6, 10] Hz, delta band [0.5, 4] Hz; see Methods). For each identified theta epoch, we extracted basic properties, including peak frequency, peak theta/delta ratio, and duration. We found that, compared to running, theta oscillations during immobility showed a largely overlapping frequency distribution with a small, but significant reduction in frequency (immobility, 7.29 ± 0.77 Hz; run, 7.54 ± 0.69 Hz; *p* = 2.37 × 10^−35^, one-tailed Wilcoxon rank-sum test) and theta/delta ratio (immobility: −0.23 ± 0.65, running: 0.51 ± 1.20, *p* = 2.52 × 10^−145^, one-tailed Wilcoxon rank-sum test; Fig. 4a). However, the spike-theta-phase relationship of putative pyramidal neurons was largely preserved during immobility (Fig. 4b), indicating that—although more variable and less prominent—LFP theta activity during immobility significantly entrains neuronal spiking.

Next, we sought to explore possible behavioral correlates of theta oscillations during immobility. Since theta frequencies (Fig. 4a), as well as whisker-pad motion and pupil dynamics (Fig. 1d), display a wide dynamic range during immobility, we reasoned that these metrics might be correlated. To test this hypothesis, we grouped behavioral data into quantiles, based on the whisker-pad motion duration (see Methods). We found that the duration of whisker-pad motion events correlated with the magnitude of pupil dilations (Fig. 4c, d); hence, in comparison to the slower pupil dynamics and their gradual buildup over time (Fig. 4d), whisker-pad motion provided a more rapid and temporally precise readout of arousal. Notably, the frequency and power of theta oscillations during immobility also correlated with these arousal-related metrics, with theta oscillations becoming progressively faster and exhibiting higher theta/delta ratio as a function of the duration of spontaneous whisker-pad motion events (theta frequency, *R*^2^ = 8.76 × 10^−2^, *F*_(2277,2275)_ = 218, *p* = 2.83 × 10^−47^; theta/delta ratio, *R*^2^ = 0.13, *F*_(2277,2275)_ = 334, *p* = 1.05 × 10^−69^, Fig. 4e, f and Supplementary Fig. 4).

Fast-spiking (FS) CA1 interneurons (classically referred to as “theta cells”^53^) have been mechanistically linked to the emergence of the LFP theta rhythm and the entrainment of pyramidal neurons in the hippocampus^42,54–56^. To further validate our observations, we hypothesized that the LFP theta frequency and power increase with the level of arousal should be paralleled by an increase in the intrinsic rhythmicity and frequency of FS interneuron discharges. To test this hypothesis, we first classified putative FS interneurons based on standard spike-width criteria (Supplementary Fig. 5; see Methods). In line with previous observations, putative FS interneurons displayed narrower spikes (Fig. 4g and Supplementary Fig. 5) and fired at higher rates than pyramidal cells (firing rate; FS interneurons: 20.31 ± 14.30 Hz, *n* = 105; pyramidal cells: 3.94 ± 4.36 Hz, *n* = 520; *p* = 1.40 × 10^−43^; Wilcoxon rank-sum test). To evaluate the spike train rhythmicity of FS interneurons, we first computed population auto-correlograms for each individual theta epoch, and then extracted both the peak frequency and power (quantified by a standard “Theta Index”^57,58^) within the theta range. Indeed, as evident from the population auto-correlograms (Fig. 4g), both the theta frequency and power (Fig. 4h) of FS interneuron spike-trains progressively increased as a function of whisker-pad motion duration (theta frequency, *R*^2^ = 3.75 × 10^−2^, *F*_(1779,1777)_ = 69.3, *p* = 1.68 × 10^−16^; Theta Index, *R*^2^ = 3.45 × 10^−2^, *F*_(1779,1777)_ = 63.5, *p* = 2.85 × 10^−15^; Fig. 4h), thus mirroring the increase estimated from the LFP theta oscillations (Fig. 4e, f).

Altogether, these data indicate that the frequency and power of LFP theta oscillations (as well as the rhythmic discharges of FS interneurons) correlate with the instantaneous level of arousal—indexed by whisker-pad motion duration, as well as by pupil area.

### Heterogeneous behavioral modulation of place cells during immobility

**Fig. 5:**
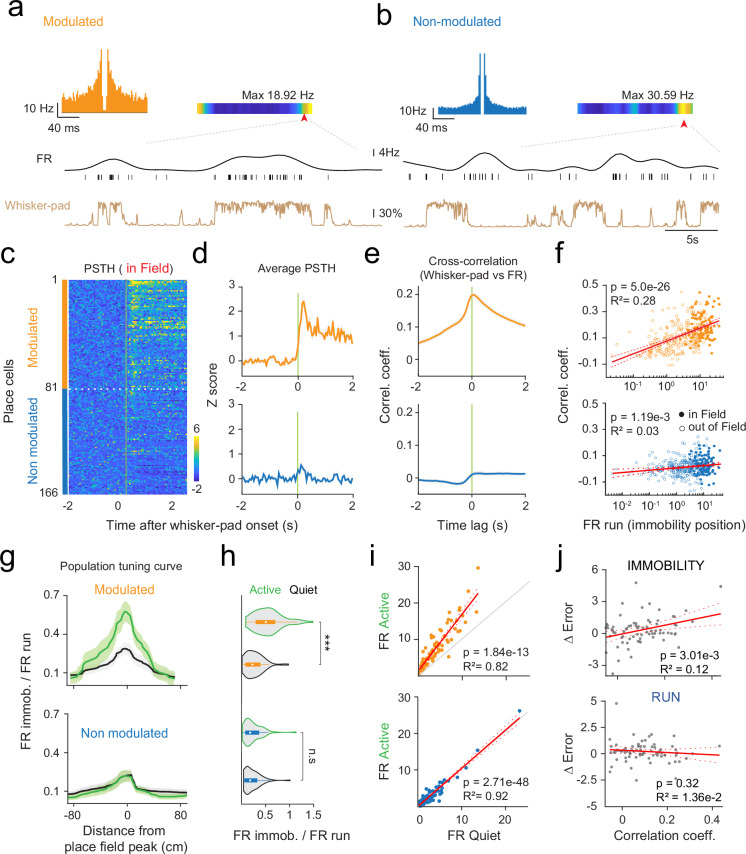
Heterogeneous behavioral modulation of CA1 place cells during immobility. **a** Representative behaviorally-modulated place cell. Top, spike-train autocorrelogram (orange) and ratemap along the treadmill (max Hz is indicated). Bottom, period during awake immobility in the field (red arrow) showing place-cell activity (spike raster and smoothed firing rate; black) and whisker-pad motion (light brown). **b** Same as (**a**) but for a non-modulated place cell. **c** Peristimulus time histogram (PSTH) of in-field place cell activity aligned to whisker-pad motion onsets, sorted by Behavioral Modulation Index. Behaviorally-modulated (orange, *n* = 81 cells) and non-modulated place cells (*n* = 85 cells) are indicated. Firing was z-scored with respect to the baseline period. **d** Group-level PSTH averages for behaviorally-modulated (orange) and non-modulated cells (blue). Same data as in (**c**). Lines indicate means; shadows indicate SEM. **e** Average cross-correlation between the instantaneous firing rate and the whisker-pad motion for behaviorally-modulated (orange) and non-modulated (blue) cells. Same *n* as in (**c**). Lines indicate means, shadows indicate SEM. **f** Behavioral modulation strength (peak of the cross-correlation defined as in (**e**)) as a function of firing rate during running at the corresponding immobility location (log_10_-transformed) for behaviorally-modulated cells (top, orange; *R*^2^ = 0.28, *F*_(341,339)_ = 132, two-sided *t*-test, *p* = 5.00e-26, *n* = 341 imm. periods) and non-modulated cells (bottom, blue; *R*^2^ = 3.06e-2, *F*_(340,338)_ = 10.70, two-sided *t*-test, *p* = 1.19e-3, *n* = 340 imm. periods). Red solid lines represent the best-fit linear regression; dashed lines indicate the 95% confidence intervals. Filled circles represent immobility periods within the field; empty circles, out of the field. No correction for multiple comparisons was applied. **g** Population average tuning curves during active (green) and quiet immobility (black). Top, behaviorally-modulated cells (*n* = 92 immobility periods, *n* = 44 cells); bottom, non-modulated cells (*n* = 132 immobility periods, *n* = 57 cells). Mean ± SEM are shown. Color codes as in (**c**). **h** In-field firing rates during immobility (normalized to in-field rates during running) of behaviorally-modulated (orange) and non-modulated (blue) cells, during active (green) and quiet (black) immobility. Violins show probability density; inner boxplots show median (white dot), IQR (solid box), and 1.5× IQR (whiskers). Active vs Quiet, modulated, *p* = 6.78e-23; non-modulated, *p* = 0.66, two-tailed Wilcoxon signed-rank test. *n* as in (**c**); *** *p* < 0.001, Bonferroni-corrected. **i** Firing rate comparison between quiet and active immobility for behaviorally-modulated cells (top, test of slope *β* ≠ 1, β = 1.56 ± 8.12e-2 SE, *t*_(79)_ = 6.87, two-sided *t*-test, *p* = 1.34e-9; linear model, *R*^2^ = 8.23e-1, *F*_(81,79)_ = 3.68e2, two-sided *t*-test, *p* = 1.84e-31) and non-modulated cells (bottom, test of slope *β* ≠ 1, *β* = 1.05 ± 3.29e-2 SE, *t*_(83)_ = 1.41, two-sided *t*-test, *p* = 0.16; linear model, *R*^2^ = 9.24e-1, *F*_(85,83)_ = 1.01e3, two-sided *t*-test, *p* = 2.71e-48). Color codes as in (**c**). Red solid lines represent the best-fit linear regression; dashed lines indicate the 95% confidence intervals. No correction for multiple comparisons was applied. **j** “Neuron dropping” procedure for general immobility (top) and running (bottom) encoding models. Immobility (*R*^2^ = 1.17e-1, *F*_(73,71)_ = 9.44, *p* = 3.01e-3); Run (*R*^2^ = 1.36e-2, *F*_(73,71)_ = 0.98, *p* = 0.33). Red solid lines represent the best-fit linear regression; dashed lines indicate the 95% confidence intervals. No correction for multiple comparisons was applied. Source data for this figure are provided as a Source Data file.

The above data (Fig. 2d–g) point to a global gain-modulation of the hippocampal representation during behavioral state fluctuations. To further explore the underlying cellular basis, we quantified the extent of behavioral modulation of individual place cells within their field. To this end, for each neuron, we computed a Behavioral Modulation Index, consisting in the difference between the peak of the cross-correlation (between whisker-pad motion and instantaneous firing rate during immobility) and the significance threshold based on the shuffled null distribution (for details, see Methods). The Behavioral Modulation Index across the place cell population did not show a clear bimodal distribution, but rather appeared to reflect a continuum (Fig. 5a–c and Supplementary Fig. 6); for analytical purposes, this distribution was partitioned into two discrete categories using statistical criteria. Specifically, approximately half of the place cells (81 out of 166) were classified as (positively) behaviorally-modulated (i.e., Behavioral Modulation Index > 0; Fig. 5a), while the other half were classified as non-modulated (85 out of 166 place cells; Fig. 5b; only 4 out of 85 non-modulated cells were classified as negatively-modulated, and hence not included as a separate group in the analysis; see Fig. 5c–e, and Methods).

During immobility, behaviorally-modulated cells exhibited higher in-field firing rates than non-modulated cells (modulated: 4.96 ± 3.88 Hz; non-modulated: 2.73 ± 3.34 Hz; *p* = 1.04 × 10⁻⁶, Wilcoxon rank-sum test); however, during running, both cell classes showed similar firing rates (average firing rates: modulated, 5.92 ± 3.68 Hz; non-modulated, 4.76 ± 2.89 Hz; *p* = 0.07, Wilcoxon rank-sum test). This indicates that while modulated and non-modulated cells cannot be distinguished by firing rates during running, their firing rate differences emerge in the absence of locomotion. As a result of this firing rate difference, behaviorally-modulated cells tended to be slightly more recruited during awake sharp-wave ripple oscillations—an effect that was abolished by downsampling of spike rates (Supplementary Fig. 3). We note however that firing rate differences during immobility were unlikely to account for the differential degree of behavioral modulation of the two cell classes since (i) behavioral modulation was still observed after down-sampling of spike rates (Supplementary Fig. 6), with the large majority of modulated cells (~ 95%; 77 out of 81) that remained significantly modulated after downsampling, and (ii) despite having similar firing rates during running, modulated cells exhibited a stronger linear relationship between firing activity during run and the degree of behavioral modulation than non-modulated cells (interaction term β between modulation group and firing rate predictor, *β* = 4.21 × 10^−2^ ± 1.12 × 10^−2^ SE, *t*_(681)_ = 3.77, *p* = 1.76 × 10^−4^, see Methods, Fig. 5f). The two cell classes were similarly distributed along the deep-superficial CA1 axis, displayed similar electrophysiological properties and were typically co-recorded within individual sessions (Supplementary Fig. 6), indicating that they are intermingled within CA1 circuits.

Modulation of spatial tuning was predominantly observed in behaviorally-modulated place cells during active immobility (Fig. 5g, h), indicating that, in the active state, the animal’s location was preferentially represented by the ensemble of behaviorally-modulated place cells. Consistent with enhanced spatial coding, behaviorally-modulated cells displayed a multiplicative gain during active immobility (i.e., linear model slope *β* ≠ 1, *β* = 1.56 ± 8.12 × 10^−^^2^ SE, *t*_(79)_ = 6.87, *p* = 1.34 × 10⁻^9^, Fig. 5i) which was not observed in non-modulated cells (*β* = 1.05 ± 3.29 × 10⁻^2^ SE, *t*_(83)_ = 1.41, *p* = 0.16, Fig. 5i; replicated in session-level analysis; not shown). To determine whether behavioral modulation indeed contributed to spatial coding, we applied a Bayesian decoding analysis combined with a neuron-dropping procedure^59,60^, quantifying the increase in decoding error following the removal of individual cells from the training set (see Methods). When using the immobility encoding model, decoding errors increased proportionally with the degree of behavioral modulation (*R*² = 1.17 × 10⁻¹, *F*_(73,71)_ = 9.44, *p* = 3.01 × 10⁻³, Fig. 5j), whereas no relationship was observed with the running encoding model (*R*² = 1.36 × 10⁻², *F*_(73,71)_ = 0.98, *p* = 0.33, Fig. 5j), consistent with the absence of spatial information (Supplementary Fig. 6) and firing-rate differences between the two cell subgroups during running. Further separation of immobility into active and quiet revealed an analogous effect only during active immobility (*R*² = 5.47 × 10⁻², *F*_(73,71)_ = 4.11, *p* = 4.64 × 10⁻²), and not during quiet immobility (*R*² = 1.13 × 10⁻², *F*_(73,71)_ = 0.81, *p* = 0.37; Supplementary Fig. 2).

Altogether, these findings show that the heterogeneity in behavioral modulation underlies differential contributions of CA1 place cells to spatial coding during immobility, with behaviorally-modulated cells being preferentially recruited into the hippocampal spatial representation as a function of arousal.

## Discussion

Storage capacity for episodic memories is limited. Hence, the brain needs to autonomously filter episodic experiences and encode only the ones worthy of retention. A large body of behavioral studies has demonstrated that this selection mechanism critically depends upon saliency and novelty signals originating from the arousal system (for reviews see refs. ^61–63^). Whether and how neural dynamics and spatial representations within the hippocampus—the neural substrate of episodic memory—are influenced by fluctuating levels of arousal has remained largely unresolved.

In the present study, we used a “space clamping” approach, and focused on awake immobility periods for capturing spontaneous fluctuations in the internal state (Fig. 1). While locomotion—the condition under which place cells have mostly been recorded—is accompanied by a homogeneous level of arousal, awake immobility is a heterogeneous behavioral state, under which arousal levels spontaneously fluctuate (Figs. 1b–d and 2a, b). Immobility offers a unique opportunity to disentangle the effect of self-motion cues from that of internal state fluctuations, since both self-locomotion and vestibular activation are sufficient to drive changes in arousal^32,34–36^. In the present study, we used a single variable (whisker-pad motion) for segmenting awake immobility into active and quiet periods, which served as a reliable correlate of arousal, consistent with recent observations^64,65^. By employing head-restrained preparations, a growing body of work has demonstrated that behavioral state fluctuations have a strong impact on neuronal gain and coding properties in primary sensory areas^36,43,66–70^ and modulate neuronal firing within brain-wide neuronal networks^44,71–74^. In the present work, we show that the place representation in the hippocampus is also dynamically shaped by behavioral state fluctuations. Specifically, we found that—along a behavioral modulation continuum (Fig. 5c–e)—about half of the place cells significantly increased their in-field firing during active immobility, while the other subset did not, indicating that the gain modulation observed at the population level (Fig. 2d–f) is primarily driven by a subset of behaviorally-modulated neurons. Our findings are in line with previous work, which showed that changes in arousal modulate the membrane potential of hippocampal pyramidal neurons^75^ and hippocampal dynamics^75–78^, and are gated by spatial location^77^. Overall, this shows that within CA1—the main output of the hippocampus—the animal’s location during awake immobility is primarily represented by the “high gain” ensemble of behaviorally-modulated place cells. Our findings thus extend previous observations by showing that during awake immobility, cell-type specific mechanisms are also engaged in the CA1, in addition to the “immobility-code” recently described in the CA2^13^.

Neuronal reactivations during awake sharp-wave ripples, which occur during quiet immobility^79^, were also biased by the animal’s location (Supplementary Fig. 3; see also refs. ^45,46^), indicating that irrespective of the behavioral and hippocampal state, the activity of CA1 pyramidal cells is systematically biased by the animal’s location (Fig. 2d–f). This observation supports the view that space is a fundamental dimension encoded within the hippocampal cognitive map^23,80^.

During running, behaviorally-modulated and non-modulated cells did not differ in firing rates or place field properties (Supplementary Fig. 6), suggesting similar levels of excitability. However, during immobility, behaviorally-modulated cells tended to fire at higher rates, and, as a result, to be slightly more recruited during awake sharp-wave ripple oscillations (Supplementary Fig. 3), pointing to a possible increase in excitation-inhibition ratios^81,82^.

The subset of behaviorally-modulated place cells might inherit modulation via direct (or indirect) inputs from the brainstem arousal centers (i.e., the cholinergic basal forebrain and the noradrenergic locus coeruleus), which have been shown to broadcast a coordinated behavioral-state signal across widespread cortical regions^65^. Another potential route for behavioral modulation of CA1 activity is CA2^83^, which projects to deep-located pyramidal neurons in CA1^84,85^ and has been shown to contain a subset of neurons that fire robustly during immobility^13,86^. Local microcircuits are also likely to be involved in shaping behavioral modulation of CA1 place cells, since different types of interneurons have been shown to be differentially modulated during immobility in the hippocampus and in cortical circuits^66,87–89^ (see also Supplementary Fig. 5). Future work will be required for testing these hypotheses and dissecting the underlying mechanisms.

CA1 pyramidal neurons show a high level of heterogeneity at the molecular, cellular, connectivity, and in-vivo properties^90–92^. Our work extends this heterogeneity by showing that CA1 neurons are differentially modulated during awake immobility, with only a fraction of place cells increasing their firing during behavioral state transitions and conveying a spatial signal. In our data, we did not find evidence for anatomical clustering of modulated and non-modulated units along the radial CA1 axis (Supplementary Fig. 6). Juxtacellular labeling^93,94^ will be required for exploring possible structure-function relationships at the single cell level.

Theta oscillations in rodents are the most prominent oscillatory clock that times neuronal spiking and organizes neural signals into packages of information^42,95,96^. Classical work in rodents has demonstrated the existence of two mechanistically distinct types of theta oscillations: fast Type-1 (atropine-insensitive) theta oscillations (7–11 Hz), which are observed during locomotion, and slower Type-2 (atropine-sensitive) oscillations (4–7 Hz), which are thought to preferentially occur during immobility^26–28,42,50,97^. Early reports suggested that Type-2 theta can be evoked by salient (aversive) stimuli^26–28^; however, evidence in rodents has remained scarce, and the behavioral correlates of Type-2 theta oscillations have remained largely unresolved. Here we show that during awake immobility, hippocampal theta oscillations span a broad frequency range, which encompasses both frequencies of Type-I and Type-II theta oscillations (Fig. 4a). Unlike during locomotion, where theta oscillations are a prominent and steady rhythm, theta oscillations during immobility occurred in short bouts (Fig. 2a and Supplementary Fig. 4), thus resembling the oscillatory dynamics described in other species (e.g., in the human^98^ and bat hippocampus^99^). Interestingly, we found that the frequency and power of theta oscillations increased with the level of arousal (Fig. 4e, f), thus offering a possible mechanistic link between the subcortical arousal- and theta-generating circuits, in line with previous suggestions^29,100,101^. The frequency of theta oscillations can be experimentally manipulated by optogenetically activating pacemaker cells within the medial septum^102^ as well as local FS interneurons within the hippocampus^103^. Consistent with these observations, we found that the spike-train rhythmicity of FS interneurons was modulated by the animal’s behavioral state. Specifically, the degree of theta rhythmicity, as well as the intrinsic frequency of spike discharges, increased as a function of arousal (Fig. 4g, h). It is noteworthy that active exploratory behaviors occurring in stationary animals—like head-scanning behavior^10^ or rearing^11^—are associated with fast (Type-1-like) theta oscillations. Most notably, during rearing—a novelty-driven behavior where the animal stands on the hind limbs and engages in sensory sampling of the environment—theta oscillations are even faster than during locomotion^11,29^. Based on this evidence and our current work, we speculate that fluctuating levels of arousal during stationary behaviors may be the prime driver of theta oscillatory regimes in the hippocampus. Future work involving, e.g., pupillometry in freely-behaving animals^104^ and causal manipulations of arousal circuits will be required for testing this hypothesis.

During spatial exploration, animals voluntarily stop and engage in sensory sampling of the local environment, concurrently displaying various associated behaviors, such as whisking, sniffing, head-scanning, and rearing^10,11,29,105–107^. These stationary behaviors punctuate exploration and are of strong ethological significance, since they are thought to mediate the encoding and retrieval of episodic experiences^10,108–110^. Indeed, recent studies have shown that head-scanning behavior and rearing are associated with rapid reorganizations of the hippocampal spatial map^10,11^. Interestingly, spontaneous spike bursts during head-scanning behavior have been shown to precede the rapid emergence of place fields^10^, indicating that place cell spiking alone might be sufficient for engaging plasticity mechanisms during immobility. To test this hypothesis, we employed juxtacellular stimulation^20,111,112^, and found that evoking spiking activity in single hippocampal neurons during instances of active immobility (Fig. 3) could be sufficient for the rapid emergence of place fields at the stimulus location. Thus, our data establish a causal relationship between spike-burst activity observed during awake immobility-related behaviors^10,16^ and place field plasticity mechanisms. Place field formation in CA1 neurons is thought to occur via rapid synaptic plasticity mechanisms at distal entorhinal inputs^16,17^. Hence, we speculate that in the absence of locomotion, spatial entorhinal inputs might still be sufficiently active to support associative plasticity upon pairing with postsynaptic activation^113^. This may support the rapid encoding of episodic experiences during immobility-related exploratory behaviors^10,107,109,110^. Future studies should investigate the behavioral state dependency of place field induction during immobility, currently tested only in the “active” state.

In summary, our data indicate that within the mouse CA1 hippocampus, a subset of place cells conjunctively encodes the animal’s location and is gain-modulated by the behavioral state. This “multiplexed code” is consistent with the general role of place cells in episodic memory, and the ability of these neurons to co-encode spatial and non-spatial episodic variables^3,114–117^. Hence, hippocampal neuronal gain- and theta-modulation might be basic mechanisms by which arousal modulates episodic memory processing.

## Methods

### Animals

Experimental procedures were performed in accordance with German guidelines on animal welfare under the supervision of the local ethics committees (Regierungspräsidium Tübingen, licenses CIN10/19G, CIN03/20G, CIN01/23G, CIN02/23G). Eight wild-type C57BL/6J mice (male, >12 weeks; Charles River, RRID:IMSR_JAX:000664) were used in this study. Mice were kept on a 12-h/12-h light/dark cycle (non-inverted) at a temperature of 20-24 °C and a humidity of 45-65%.

### Surgical procedures, behavioral training, and silicon-probe recordings

Surgery and implantation were performed as previously described^20,93,118^. Briefly, mice were anesthetized by intraperitoneal injection of ketamine/xylazine or with a mixture of fentanyl, midazolam, and medetomidine^49^ and implanted with a custom-made head-post. Craniotomies ( < 1 mm^2^) were performed at the coordinates for targeting the dorsal CA1 field of hippocampus (2 mm posterior from Bregma; 2 mm lateral from midline).

The linear treadmill consisted of a circular black belt (210 cm length; LN Treadmill, Luigs and Neumann GmbH) enriched with visuo-tactile cues (color tape patterns and velcro strips)^37,38^. To estimate the animal’s position, the treadmill was equipped with a rotary encoder to track wheel rotation, and an infrared photo-sensor was used to reset the position signal on a lap-by-lap basis. A licking spout was placed below the animal’s mouth to deliver water rewards at a specific location on the belt (see below). Awake immobility data were experimentally controlled and collected by “space clamping” the position of the mouse by means of a servo motor (3242G012BX4, Faulhaber), which acted as a brake for the belt. Custom MATLAB code was used for the online control of the brake via an Arduino.

After a post-surgery recovery period, mice were trained to run on the treadmill by collecting water rewards enriched with sucrose (5–10 µl, 10% sucrose) delivered in a defined zone of the belt. Access to water was restricted to 1 ml/day on a 5/7-day schedule. Once the animals could run comfortably, they were habituated to the immobility condition by introducing random immobility periods throughout the session (gradually increased from 10 s to the target duration of 5 min). To facilitate training, a white dim LED was used as a cue (positioned at the end of the treadmill and oriented towards the wall) and signaled when the animal was allowed to run. The immobility locations (between 1 and 9 per session) were randomly distributed and spanned the entire belt (see Supplementary Fig. 1b).

Silicon probe recordings were performed with commercially available, two-shank 16 or four-shank 32 channel electrodes (Buzsaki16-A16, Buzsaki32-A32; NeuroNexus). The electrode shanks were painted with DiI (D12730, Invitrogen), and the electrode recording location in the CA1 region was verified by post-hoc histological analysis. Recordings were performed as previously described^118^. Briefly, during each recording session, the probe was slowly advanced into the brain tissue using a standard micromanipulator (LN-Junior, Luigs and Neumann). After reaching the CA1 pyramidal layer (signaled by the presence of sharp-wave ripples^20,94,119^), the craniotomy was sealed with liquid agarose (1.5% in PBS solution) to aid stability during recordings. Recording sessions lasted ca. 45 minutes (42.7 ± 15.5 min). Run and immobility conditions were alternated, as during the training sessions.

### Juxtacellular recordings and stimulation

Juxtacellular stimulation recordings, signal acquisition, and processing in awake, head-fixed animals were performed as previously described^20,111,112,120^. Briefly, glass electrodes with resistance 7–9 MΩ were filled with 1.5–2% intracellular solution containing (in mM): 135 K-gluconate, 10 HEPES, 10 Na2-phosphocreatine, 4 KCl, 4 MgATP, and 0.3 Na3GTP (osmolarity adjusted to 280–310 mOsm). After a baseline recording, silent cells or place cells were stimulated at a random location during awake immobility. To evoke spiking activity during periods of active immobility, juxtacellular single-cell stimulations were paired with a sound stimulus (Fig. 3a), thereby ensuring that evoked spiking occurred during the active state, as signaled by the onset of whisker-pad motion and pupil dilation (Fig. 3b). Specifically, each current pulse (200 ms duration) was preceded by a “click-like” sound (duration of 30 ms; 65–80 dB), which is known to induce a transition to an active immobility state signaled by a stereotypical response in whisker-pad motion and pupil dilation^49^ (see Fig. 3b). The current pulse was delivered at two different delays from the sound stimulation (75 or 500 ms) to be coincident with the maximum whisker-pad motion or pupil dilation (see Fig. 3b). Additional juxtacellular and extracellular “sound-only” controls were conducted as described above, where only the sound was presented without juxtacellular stimulation (see Fig. 3c–g).

### Data acquisition

A custom-made brakebox served as an interface for all treadmill signals. The animal’s position on the belt was computed in real-time with a Real-Time Target machine (Baseline S, Speedgoat GmbH) interfaced with Simulink (MATLAB), running a custom model (MATLAB 2019b). The same system was used to control the reward delivery. The pupil and the whisker-pad motion were monitored during the behavioral task by means of an infrared camera (DMK33UX265, TheImagingSource), imaging the face of the animal from the side. The camera was externally triggered at 100 Hz, and videos were acquired using the “IC Capture” software (The ImagingSource). To increase contrast, the animal’s face was illuminated with an infrared LED light source (M850L3, Thorlabs; LEDD1B Driver, Thorlabs). Electrophysiology data and all treadmill signals were collected at 30 kHz with an Open Ephys acquisition system and a RHD2132 headstage (16, 32ch, Intan Technologies).

For juxtacellular recording and stimulation experiments, signals (including sound stimuli) were recorded with a Powerdata-acquisition interface (CED1401, Cambridge, UK) under the control of Spike2 v.8.02 software (Cambridge, UK) and sampled at 25 kHz. Ephys data were amplified with an ELC-03XS amplifier (NPI Electronic).

### Data analysis

#### Analysis of behavioral data

Pupil area and whisker-pad motion energy (ROI centered on the whisker pad) were extracted and analyzed with the open-source software Facemap (https://github.com/MouseLand/facemap 19) in Python^121,122^. Occasional artifacts in the whisker-pad motion trace (i.e., large-amplitude signal deflections and peaks) were removed by clipping (> 1st and <99.9th percentile). The whisker-pad motion trace was then normalized in the min-max range (from 0 to 1) and a moving mean was applied (with a window size of 250 ms). The pupil area was z-scored within the session with reference to the pupil area during running.

Active immobility periods were identified when whisker-pad motion was greater than 0.1, while quiet immobility periods were identified when it was less than 0.1 (see event distributions in Supplementary Fig. 1e, f). Only whisker pad motion events with a duration of at least 0.5 s were included in the analysis. The analyses shown in Figs. 2 and 5g–i included only sufficiently long active and quiet immobility periods (> 1.5 s) in order to capture steady-state dynamics (see Supplementary Fig. 5c, d), as done in previous work^75,123,124^.

#### Spike sorting and unit classification

Spike sorting was performed offline with Kilosort 2.5 software^125^ (https://github.com/MouseLand/Kilosort) and the visualization for manual curation was performed in Phy2 (https://github.com/cortex-lab/phy) with the aid of extra plugin tools (https://zenodo.org/records/3367782). Noise clusters were manually identified by waveform and autocorrelogram features. Potential single- and multi- units were identified in all recordings, and only single, well-isolated units were included in the analysis. Single isolated units were further classified into putative pyramidal neurons and FS interneurons using a 2-D Gaussian mixture model (GMM) based on spike waveform features (spike half-width and trough-to-peak time). Only units belonging to a cluster with a posterior probability greater than 0.95 were included in the analysis^126^. From a total of 644 CA1 units, 520 were classified as putative pyramidal neurons and 108 as putative FS interneurons (see Supplementary Fig. 5b). Misclassified units with atypical waveforms were either manually labeled (*n* = 7 units) or discarded as noise (*n* = 2 units).

#### Place cell analysis

For each putative pyramidal unit, ratemaps were computed by including only spikes occurring during movement (where the speed > 1 cm/s). To this end, the treadmill belt was discretized into bins of 2.1 cm (*n* = 100 bins), and, for each bin, the spike count was divided by the occupancy time^37,38^. Ratemaps were smoothed with a Gaussian kernel (width = 12.6 cm; 6 bins). To assess the statistical significance of spatial modulation, a standard circular shift was used. Specifically, for each trial of the shuffling procedure, spike times were randomly time-shifted (minimum ± 5 s). When shifted spike times exceeded the total duration of a recording, they were wrapped around to the beginning of the recording. For each permutation, the ratemap was calculated, and this procedure was reiterated 1000 times to generate a null distribution for each cell. Spatial bins with a *p*-value < 0.01 were considered to be significant^38,119^. Place fields were defined as contiguous significant spatial bins, with a minimum size of 7 bins (14.7 cm). Accordingly, the place field size was defined as the length of significant bins. Units classified as putative pyramidal neurons displaying at least one place field were classified as place cells (*n* = 340 out of 520). No difference in the distribution of place fields around the reward zone (between 126 and 158 cm, centered on the reward location) was observed for modulated and non-modulated cells (modulated: 16/81 cells; non-modulated: 20/85 cells; *p* = 0.58, Fisher’s exact test).

Place cell recordings were included in the analysis according to additional spatial stability criteria. Specifically, Pearson’s correlation between the ratemaps of the running epochs before and after each immobility period was computed and served as a stability index (see Supplementary Fig. 1c). Only portions of recordings with a stability index greater than 0.7 were included in the analysis (see Supplementary Fig. 1c). Behaviorally-modulated and non-modulated cells (Fig. 5) did not differ in spatial stability (pre-post immobility ratemap correlations, *p* = 0.58, Wilcoxon rank sum test). Immobility periods with less than 50 spikes and run periods with less than 100 spikes were excluded from further analyses. Only units with more than two consecutive stable immobility periods were included in the analysis. Although only a single environment (belt) was used in the present study and spatial representations were generally stable, we acknowledge that—even with our stability criteria during immobility—we cannot formally exclude the possibility of transient flickering between maps at the theta-cycle timescale^127^.

The coefficient of variation (CV) was obtained by dividing the standard deviation by the mean of the interspike interval (ISI)^128^. The burst index was computed as the sum of spikes with an ISI ≤ 6 ms, divided by the total number of spikes^129,130^. Spatial information in place cell units was computed as follows^131^:

$${I}_{{spike}}={\sum}_{n}({p}_{n}*\,\frac{{\lambda }_{n}}{\lambda }*{\log }_{2}\frac{{\lambda }_{n}}{\lambda })$$

The sparsity index of a given place cell^131,132^ was calculated as:

$${Sparsity}=\,\frac{{\lambda }^{2}}{{\sum}_{n}{p}_{n} \,{\lambda }_{n}^{\,2}}$$

In both cases, *p*_*n*_ is the probability of the animal being in *n*th space bin, *λ*_n_ is the mean firing rate in the *n*th space bin, and *λ* is the overall mean firing rate of the place cell unit.

To estimate the anatomical location of recorded units (Supplementary Fig. 6f, g), we used as a reference channel the one with the highest ripple power (150–250 Hz) during each recording session, and channels were numbered accordingly, resulting in an anatomical location index for each unit (following the procedures described in ref. ^133^; positive means ventral, i.e., towards the stratum radiatum). Place cells were considered to belong to the deep layer when the anatomical location index was negative, and to the superficial layer when the anatomical location index was positive. Only shanks coupled with putative pyramidal cell activity, and whose peak in ripple power was not detected in the first or last channels of the shank, were considered for this analysis.

#### Analysis of place cell firing during awake immobility

To visualize the relationship between place cell firing during immobility and place cell spatial tuning during running (Figs. 2e and 5g), population tuning curves during active and quiet immobility were constructed (since individual tuning curves could not be computed because not all belt locations were systematically sampled during immobility within each session). To this end, immobility periods from place cells with a single place field (*n* = 225 “single field” place cells, identified as described above) were sub-selected, including in the analysis only “in field“ immobility periods and “out of field“ immobility periods coupled with firing rates <2 Hz during running at the corresponding location (resulting in a selection of *n* = 280 immobility periods, coming from *n* = 131 out of 225 “single field” place cells). For each immobility period, firing rates were normalized by dividing them by the in-field firing rate during running. The signed distance of the animal from the place-field peak was computed, with positive values indicating that the animal’s position was ahead of the place-field center. Normalized firing rates and signed distances were then combined, and a trendline was fitted to assess their relationship. For this, a moving window (30-element size) was used to compute the median firing rate and signed distance, and a moving average was applied for smoothing (with a window size of 25 elements). Population tuning curves for modulated and non-modulated (Fig. 5g) were computed in the same way, using subsets of the data described above (modulated: *n* = 93 immobility periods from *n* = 44 out of 81 cells; non-modulated: *n* = 139 immobility periods from *n* = 57 out of 85 cells). These population tuning curves were used solely for display purposes (Figs. 2e and  5g).

For in-field and out-of-field quantifications of place cell activity during immobility (see Fig. 2f), place cells were classified into two subgroups: those with immobility periods within their place field (“in-field” cells; *n* = 166 of 340) and those with immobility periods outside their place field (“out-of-field” cells; *n* = 164 of 340). Notably, the majority of “in-field” cells were also recorded during out-of-field immobility (“in- & out-of-field” cells; *n* = 105 of 340). If cells had multiple immobility periods in the field, behavioral and spiking data were combined. For each cell, the out-of-field immobility period was chosen based on the lowest firing rate during running at that corresponding spatial location. Only periods in which the running firing rate was <2 Hz were included. Firing rate normalization was performed by dividing the firing rates during immobility by the firing rates in the field during running (defined as the firing rate of the closest spatial bin in the ratemap).

#### Behavioral modulation analysis

To quantify the degree of behavioral modulation of individual place cells during immobility, a cross-correlation (max lag 3 s) was computed between the instantaneous firing rate (20 ms bins, smoothed with a Gaussian kernel, 250 ms width) and the whisker-pad motion trace (as described above) during the whole immobility period in the field. Statistical significance of the cross-correlogram peak was assessed with a shuffling procedure (e.g., ref. ^134^). Specifically, a null distribution was constructed by applying a random circular shift (of at least 3 s) to the whisker-pad motion trace. For each shuffle, a cross-correlogram between the instantaneous firing rate and the shifted whisker-pad motion trace was computed, and the procedure was reiterated 1000 times. The 99% confidence interval based on the null distribution was later used to classify “in field” place cells into two classes (*n* = 166 cells). Specifically, cells with a cross-correlogram peak (in the lag range [0,1] s) that crossed the upper boundary of the confidence interval were classified as positively modulated (“behaviorally-modulated” cells, *n* = 81 cells), and the rest were classified as non-positively modulated (“non-modulated” cells, *n* = 85 cells). The Behavioral Modulation Index was defined as the difference between the cross-correlation peak (in the lag range [0,1] s) and the upper boundary of the confidence interval (i.e., > 0 for positively modulated cells, <0 for non-modulated cells). Only a minority of cells (*n* = 4) from the non-modulated group were found to be negatively modulated (i.e., cells with a cross-correlogram trough in the lag range [0,1] s which crossed the lower boundary of the confidence interval). Given their very small number, they were not considered as a separate group for subsequent analysis. For FS interneurons (Supplementary Fig. 5e), behavioral modulation statistics were computed independently for each stable immobility period, and combined by averaging (Supplementary Fig. 5e). Spike downsampling was implemented by randomly removing spikes during immobility periods from behaviorally-modulated units to match the median firing rate of non-modulated units (Supplementary Figs. 6b and 3g)^135,136^.

#### LFP analysis

The local field potential (LFP) signal was downsampled to 1250 Hz. High-frequency artifacts were identified and excluded from further analysis. Time-resolved spectrograms were computed using the multitaper method (1.5 s window, in the [0.5, 100] Hz band, Chronux toolbox, http://chronux.org). Theta/delta ratios were calculated by dividing the power in the theta range (6–10 Hz) by the average power in the delta range (0.5 to 4 Hz) and scaled to dB units (in a single animal, the delta range was restricted to 2–5 Hz due to the presence of low-frequency artifacts). The gamma range was between 70 and 90 Hz. In Fig. 1d, we extracted the peak power frequency across the entire spectrogram, and the frequency distributions for running and immobility were compared with a two-tailed Kolmogorov-Smirnov test (Bonferroni-corrected).

#### Theta oscillations segmentation and analysis

To segment theta oscillations during awake immobility, the LFP channel with the highest theta/delta ratio during each session was used (*n* = 32 sessions; due to the presence of low-frequency artifacts, 2 sessions out of 34 were excluded from this analysis). To this end, due to the low signal to noise ratio during immobility, we derived a per-session threshold, defined as the intersection of the theta/delta ratio distributions during immobility and running. Periods of theta/delta power during immobility above this threshold were considered as theta epochs. The onset and offset of each event were identified with a second threshold defined as the median between the first identified threshold and the peak of theta/delta power during immobility. Events with onsets closer than 1 s were merged. For each individual theta epoch, peak frequency, peak power, and duration were identified.

Given the temporal overlap between whisker-pad motion events and theta onsets (Supplementary Fig. 4c), we defined overlapping periods as associated, and linear correlations between theta properties and whisker-pad motion duration (Fig. 4f, h) were performed on these associated events (2277 from 4288 total theta epochs). Whisker-pad motion durations longer than 0.5 seconds were included in this analysis. For qualitative assessment (see e.g., Fig. 4d, e, g), all whisker-pad motion events were grouped in five quantiles, based on the whisker-pad motion duration. In Fig. 4a, for comparing theta properties during running versus immobility, theta epochs during running were extracted from each session by matching the duration and inter-event intervals of immobility theta periods, only events within the range 5–10 Hz were included for this analysis. Time-resolved changes in theta frequency and power after the whisker-pad onset in Supplementary Fig. 4d were extracted by finding the peak power in the 3–25 Hz range of the spectrogram. For analysis of power changes in oscillatory bands for different quantiles of whisker-pad motion duration in Supplementary Fig. 4, individual events with artifacts were removed; only 2177 from 2277 total theta epochs were included in the analysis.

#### Spike-theta phase analysis and theta rhythmicity

The LFP channel with the highest theta power in each recording session was band-pass filtered in 14 steps (0.85 Hz window each) in the range between 1 and 13 Hz. For each frequency step, the instantaneous theta phase was computed using the Hilbert transform. Each spike phase was obtained by interpolation with the instantaneous phase vector^37^. Theta phases were binned (*n* = 15 bins) and a phase histogram was constructed using all spikes from pyramidal cells (Fig. 4b) and FS interneurons (Supplementary Fig. 5f) during a recording session, independently for locomotion and immobility. The mean Rayleigh vector length and Rayleigh test *p*-value were computed to quantify the strength and statistical significance of spike entrainment in both conditions and across frequencies^137,138^ (Fig. 4b and Supplementary Fig. 5f). Bonferroni correction was applied to *p*-values for the number of frequency steps tested. Only spikes occurring during theta epochs (as identified in the segmentation) were used. For session analyses shown in Fig. 4b and Supplementary Fig. 5f, significance bars indicate the bins for which >75% of the sessions showed significant entrainment of spiking activity.

Theta rhythmicity of interneuronal spiking was quantified by first computing, for each theta epoch, a population train autocorrelogram from putative FS interneurons (half-width 500 ms, bin size 5 ms). To evaluate the spike train rhythmicity of FS interneurons, we computed the Theta Index as described in refs. ^57,58^. Briefly, the FFT of individual autocorrelograms was computed, and the power spectrum was squared and smoothed with a moving mean filter (2 Hz). The peak frequency was identified (6–10 Hz range) and the theta index computed as the mean power ±1 Hz around the peak divided by the mean power in 0–50 Hz range. To ensure a better estimation of the rhythmicity parameters, only autocorrelograms with >100 mean instantaneous firing rate and Theta Index >0.5 were included in the analyses shown in Fig. 4g, h (1779 out of 1839).

#### Sharp-wave ripple detection and analysis

Sharp-wave ripples were detected as previously described^13,139,140^. Briefly, the pyramidal layer was identified (i.e., the channel with the highest number of putative pyramidal neuronal spikes), and the corresponding LFP traces of the pyramidal layer channel (and the channels above and below it) were bandpass filtered in the ripple range (150–250 Hz). Then, the envelope of the Hilbert transform was computed (envelopes were squared and summed together). The resulting trace was then smoothed with a Gaussian kernel (4 ms width), and its square root was z-scored. Events crossing 4 standard deviations (SD) for more than 30 ms were considered as candidate ripple events. To identify the onset and the offset of the ripple event, the signal was followed backward and forward until it reached the threshold of 1 SD. Candidate ripples longer than 500 ms were discarded. Due to the occasional presence of high-frequency artifacts in the LFP trace during active immobility, candidate ripples that did not coincide with the reactivation of pyramidal cells were discarded (see Supplementary Fig. 3b). For this, the fraction of pyramidal cells reactivated during each sharp-wave ripple event occurring during quiet immobility was calculated, and the median value was taken as a reactivation threshold. For analyses reported in Supplementary Fig. 3d–g, only SPW-R occurring during quiet immobility (i.e., in quiet epochs > 1.5 s) were considered in the analysis.

The reactivation index was defined as the percentage of ripple events that contained spikes of a given place cell in a window spanning ± 50 ms around the ripple power peak (Supplementary Fig. 3f, g). The average number of spikes per ripple event (Supplementary Fig. 3f, g) was computed similarly, by dividing the total number of spikes in ripples by the number of ripples. Spike down-sampling was conducted as described above (see section “Behavioral modulation analysis”) to match the median firing rate of place cells out of the field (in Supplementary Fig. 3f) or the median firing rate of non-modulated place cells (in Supplementary Fig. 3g) during quiet immobility. SPW-R-coupled reactivations were identified as described in ref. ^141^. Briefly, population activity during immobility was binned (1 ms window), Gaussian smoothed (20 ms window), and z-scored. Reactivation events were identified when the normalized trace exceeded 3 standard deviations, and event onsets and offsets were identified when the normalized trace reached its average. Only reactivation events that lasted between 50 ms and 2 s, and whose peak was in the vicinity of a SPW-R (± 0.5 s) were considered in the analysis. Sessions in which the average SPW-R profile did not exhibit the polarity reversal across the pyramidal layer (as shown in Supplementary Fig. 3b) were excluded from the ripple analysis (*n* = 4 out of 34 sessions).

#### Bayesian decoding analysis

A Bayesian decoding algorithm^142^ was used to compute the maximum likelihood estimate of the animal’s location from place cell firing in non-overlapping time windows, either during running epochs or during immobility (window size: *τ* = 0.2 s). A uniform prior over position was used. The analysis was performed on portions of recordings where place cells exhibited stable firing during running. For each cell, firing stability was assessed by normalizing running-epoch firing rates to the cell’s maximum rate, and only sections with a normalized rate ≥ 0.5 were included. Analysis was further restricted to the longest recording segment containing the majority of stable place cells. After this stability correction, only sessions with more than four cells and at least two immobility periods were included (*n* = 22 sessions, *n* = 180 out of 340 total place cells).

The decoding analysis was restricted to locations of immobility (see Supplementary Fig. 2a, b). When training on running epochs, encoding models were computed using the bins in the “run” ratemaps (computed as described in section “Place cell analysis”) corresponding to locations neighboring immobility periods. When training on immobility, encoding models incorporated each cell’s firing rates during each immobility period, contextual to active immobility (used in Fig. 2g and Supplementary Fig. 2c, d), quiet immobility (used in Fig. 2g and Supplementary Fig. 2c, d) or general immobility (used in Fig. 5j). The duration of training data for active and quiet immobility was matched across periods (using the minimum duration), and testing data duration was matched within each immobility period. Epochs of active/quiet immobility > 1.5 s were included in the analysis. When training and testing occurred both on running epochs, decoders were trained on the 2nd half of the session and tested on the 1st half (Fig. 2g, Supplementary Fig. 2c, and Fig. 5j); otherwise, no restrictions were applied. When decoding the running location, only predictions coupled with speeds greater than 1 cm/s and positions close to immobility locations (ε ± 10.5 cm) were considered in the analysis (see Supplementary Fig. 2a).

Decoding errors were estimated by calculating the root mean square error (RMSE) for each immobility location (see Supplementary Fig. 2b). Additionally, to account for the discrete and non-contiguous distribution of immobility locations inherent to our “space-clamping” approach—which precludes direct comparison with standard absolute error metrics—RMSE was z-scored with reference to a null distribution for the error where predictions were randomly assigned to immobility locations (see also Supplementary Fig. 2b; 500 shuffles). The session-level error was calculated by averaging the RMSEs of the immobility periods and normalized in the same way (Supplementary Fig. 2b).

In the neuron-dropping procedure (Fig. 5j; refs. ^59,60,143^), decoders were trained on general immobility (or active/quiet immobility, Supplementary Fig. 2d) and running epochs. The original decoding error (error_original_) was compared to the error when a specific cell was omitted from training (error_drop_). For each cell, the increase in error during in-field immobility periods was calculated as Δerror = error_drop_–error_original_. If multiple immobility periods occurred within a field, only the period with the lowest original error was considered. Only cells with immobility periods in the field and sessions with more than 5 place cells were included in this analysis (*n* = 73 out of 166 “in field” cells, *n* = 13 sessions).

Bayesian decoding was used to estimate the animal’s offline position from the firing of place cells during SPW-R-coupled reactivations (window size: *τ*_react_ = 20 ms, with a 5 ms overlap, Supplementary Fig. 3d, e). Here, place-cell ratemaps were used as encoding models (computed as described in section “Place cell analysis”; 100 spatial bins). Maximum likelihood location predictions within reactivation events whose posterior probability was at least five times above chance level (0.05, 5 × 1/total position bins^141^) were binned in space (25 bins) and their distribution was normalized (Supplementary Fig. 3d, e, *n* = 19 sessions out of 22 decodable sessions; 3 were discarded because of the absence of polarity reversal across the pyramidal layer). In Supplementary Fig. 3c, predictions were binned in a confusion matrix (10 spatial bins) and each column was normalized by dividing by the total number of predictions in a specific location (i.e., each column) (*n* = 19 sessions).

#### Analysis of juxtacellular single-cell stimulation data

Single-cell juxtacellular recordings were performed under two conditions: sound paired with electrical stimulation (*n* = 44 cells; see paragraph “Juxtacellular recordings and stimulation”) and sound-only controls (*n* = 20 cells). Additional extracellular sound-only control experiments (60 putative pyramidal cells from 3 recording sessions) were performed, and single-units were spike-sorted as described above (see section “Spike sorting and unit classification”). For both sound-only controls and juxtacellular stimulation experiments, the recording protocol included a baseline recording (“Pre-run”), an immobility period during which either sound alone or sound followed by juxtacellular stimulation was delivered (Fig. 3c), and a second run period (“Post-run”). For extracellular recordings, units were excluded if the average firing increase across the belt between pre- and post-condition exceeded 3 standard deviations (*n* = 1 unit discarded).

Ratemaps for the pre- and post-run were computed as described above (see section “Place cell analysis”), shifted circularly so that the animal’s location was at the center of the ratemap vector, and smoothed with a Gaussian kernel (width = 25.2 cm; 12 bins). A differential ratemap was then computed by subtracting the pre-run ratemap from the post-run ratemap and divided by the median firing increase, respectively, to the recording methodology used (i.e., to factor out the intrinsic difference in firing rates between juxtacellular and extracellular recording configurations). In Fig. 3e, computed solely for display purposes, positive firing rate increases are shown (firing rate decreases in the differential ratemap set to NaN).

Effects of “sound-only” and “sound + juxtacellular” stimulation were quantified at the single-cell level by first computing the maximal firing increase around the stimulus location (−10.5 to 10.5 cm) and normalizing it using a median z-score. Cells with a normalized increase >1 SD were considered responsive, provided the increase was localized: firing within the stimulus location ([−10.5,10.5] cm) had to exceed that outside the stimulus ([−110,−63] ∪ [63,110] cm). Only cells meeting both criteria were classified as responsive (11 effects out of 44 cells for juxtacellular stimulation; 2 effects out of 80 for sound-only controls).

#### Dataset overview

From a total of 644 CA1 extracellular units (*n* = 34 recording sessions from *N* = 5 mice), 520 units were classified as putative pyramidal neurons and 108 units as FS interneurons, based on spike waveform features (see section “Spike sorting and unit classification”). Among FS interneurons, 105 out of 108 units were included in the analysis because of additional firing stability criteria (see section “Behavioral modulation identification” for details). 340 out of 520 putative pyramidal units were classified as place cells (see criteria in “Place cell analysis”) and grouped for analysis purposes into: “in field” group (*n* = 166 units, used for analysis related to Figs. 2 and 5, further classified in behaviorally modulated, *n* = 81 cells, and non-modulated, *n* = 85 cells); “out of field” group (*n* = 164 units, used for analysis related to Fig. 2), and “*in & out of field*” group (*n* = 105 units from *n* = 26 sessions, used for per-session validation analyses in Supplementary Fig. 2e). For the Bayesian decoding analysis, to ensure ratemap stability where decoding is performed, we applied additional stability criteria on place cell firing, which resulted in the inclusion of *n* = 180 out of 340 place cells (Fig. 2g and Supplementary Fig. 2c; *n* = 22 sessions); and 73 out of 166 “*in field*” cells (from 13 sessions) for the impact of behavioral modulation analysis shown in Fig. 5j and Supplementary Fig. 2d (details in “*Bayesian decoding analysis*” in Methods). The stimulation dataset (Fig. 3) included 44 juxtacellularly-stimulated and 80 sound-only stimulated putative pyramidal neurons (*n* = 20 juxtacellular, *n* = 60 extracellular). A subset of juxtacellularly recorded cells (*n* = 4) underwent both sound and juxtacellular stimulation sequentially, as illustrated by the representative cell in Fig. 3c.

#### Statistical analysis

Statistical analysis was conducted using MATLAB® 2022a (MathWorks), and non-parametric tests were used, unless described in individual figure legends. Wilcoxon rank-sum tests, Wilcoxon signed-rank tests, and Kruskal–Wallis tests (followed by a post-hoc test) were conducted as appropriate for each data set (information provided in the corresponding figure legend and Results section). Two-tailed tests were conducted, unless stated otherwise. Linear regression models were computed using the function fitlm (MATLAB®); the *R*^2^ values reported were not adjusted for multiple regressors. In Fig. 4f, h and in Fig. 5f, a log transformation was applied to the predictor before fitting linear models. Circular statistics, Rayleigh, and z statistics were computed using the Circular Statistics Toolbox. *P* values, *n*, and specific tests are described in each figure legend. The alpha threshold for significance was set at 0.05. For multiple comparisons, *p*-values were adjusted with Bonferroni correction, unless stated otherwise (for example, in Supplementary Fig. 6d–g, *p*-values were not corrected, due to the exploratory nature of the analysis). Data within the “Results” section are reported as mean ± standard deviation and in figures as mean ± standard error, unless stated otherwise.

### Reporting summary

Further information on research design is available in the Nature Portfolio Reporting Summary linked to this article.

## Supplementary information

Supplementary Information

Reporting Summary

Transparent Peer Review file

## Source data

Source Data

## Data Availability

Preprocessed raw data are available at: https://github.com/BurgalossiPublic/HP-BehModulation. Source data are provided with this paper.

## Code availability

Custom code associated with this study is available at: https://github.com/BurgalossiPublic/HP-BehModulation.
