# Supplementary material for: Place and behavioral modulation of hippocampal neurons during immobility
Source: Nat Commun. 2026 Jul 24;17:7261. doi: 10.1038/s41467-026-75492-w (PMC13400744; doi:10.1038/s41467-026-75492-w)
Supplement: Supplementary file 1 — Supplementary Information [file 41467_2026_75492_MOESM1_ESM.pdf]

Supplementary Information

Place and behavioral modulation of hippocampal neurons during immobility

Supplementary Figures

Supplementary Fig. 1

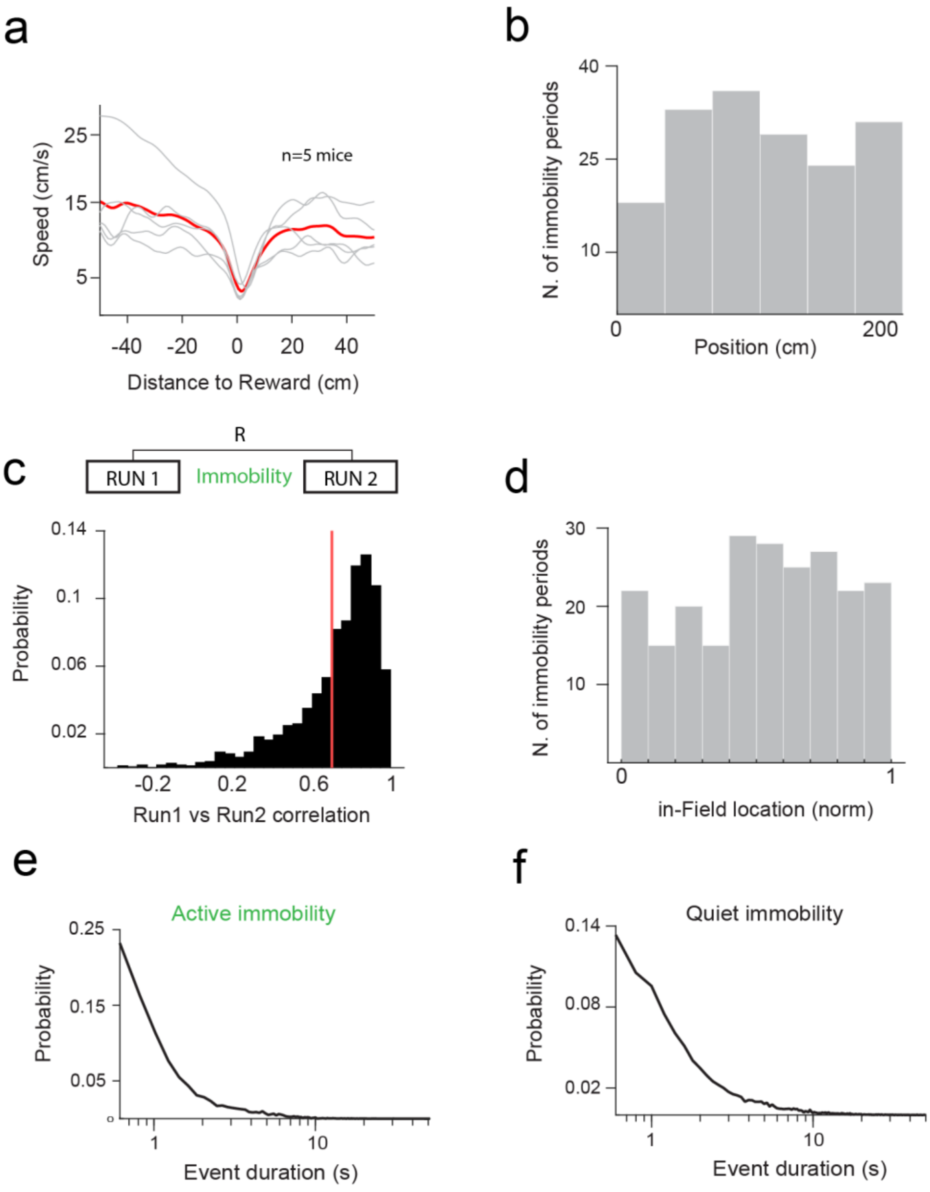

Supplementary Fig. 1. Behavioral data and place cell stability.
(a) Average speed profiles for each animal (gray) aligned to the reward position on the treadmill (n=5 mice). The average speed profile across animals is shown in red.
(b) Distribution of the position of all immobility periods along the treadmill belt (n=171 immobility periods coming from n=34 sessions).
(c) Ratemap Pearson's correlations (R) for all adjacent running periods (RUN1 and RUN2) separated by an immobility period. The red line indicates the stability threshold (R=0.7; see Methods). n=340 place cells resulting in n=1974 immobility periods, n=1143 stable immobility periods.
(d) Distribution of all stable immobility periods occurring within the field (n=226 immobility periods).
(e) Distribution of duration for all active immobility periods (n=6459 events).
(f) Distribution of duration for all quiet immobility periods (n=9135 events). Source data for this figure are provided as a Source Data file.

Supplementary Fig. 2

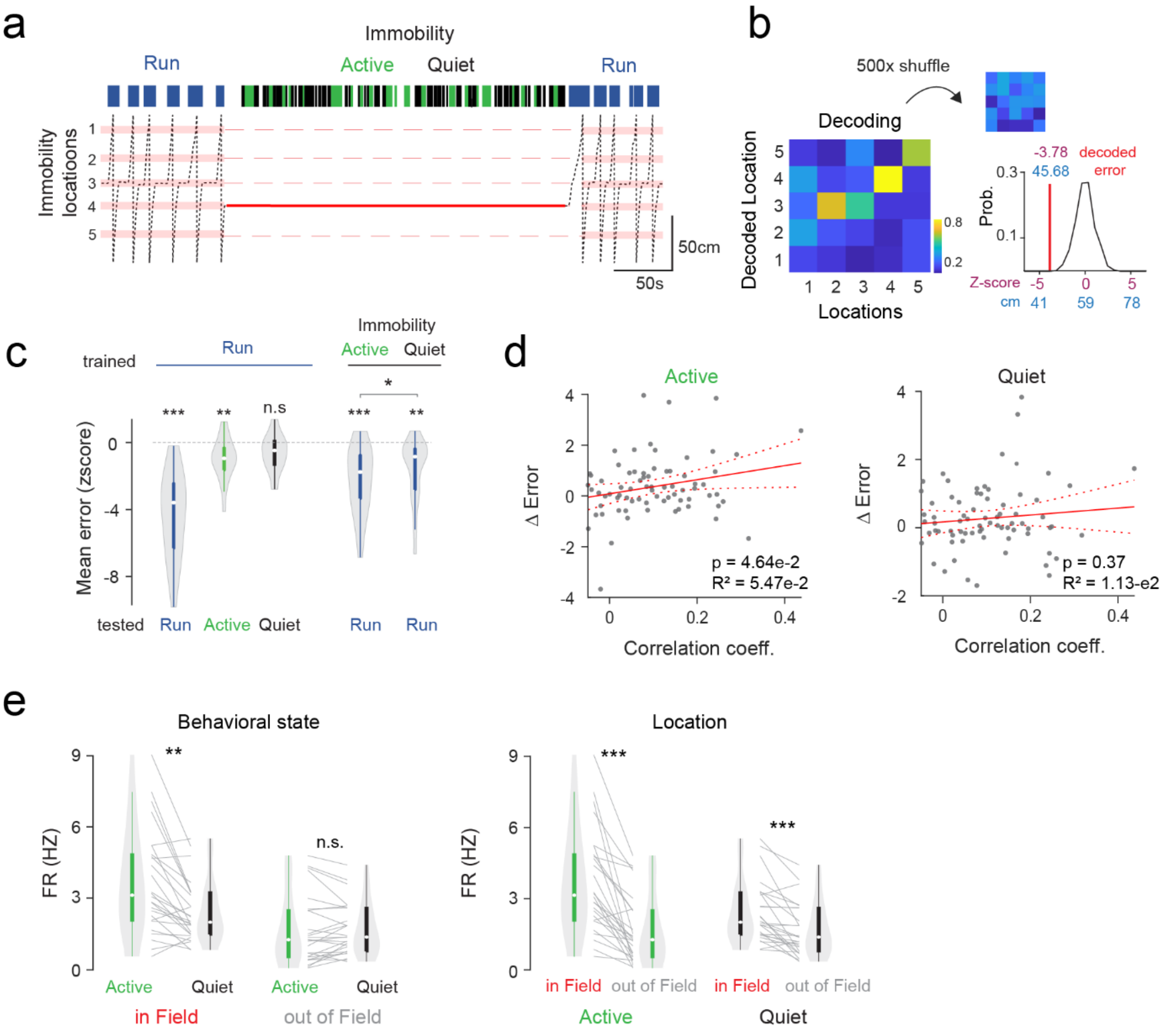

Supplementary Fig. 2. Bayesian decoding during awake immobility.
(a) Decoding analysis in a representative session. Top: behavioral segmentation into running (blue), active immobility (green), and quiet immobility (black) epochs used in the analysis. Bottom: position trace (dashed black) with one immobility period highlighted by a solid red line; the other four immobility periods recorded in this session are indicated by red dashed lines. Training and testing during running were restricted to these five immobility locations, highlighted by red shaded rectangles (see details in Methods, “Bayesian decoding analysis”). Various training-testing combinations across active immobility, quiet immobility, and running were conducted, as illustrated in (c) and in Fig. 2g.
(b) Left, confusion matrix showing decoding accuracies for the representative session shown in (a). In this example, training was conducted on active immobility and testing on running periods. Right, errors (in cm) were z-scored with reference to a null distribution computed from 500 shuffled confusion matrices (an example is shown on top). The error normalization shown is at session-level, decoded error and z-score value are shown (blue, cm; purple, z-score); an analogous normalization procedure was conducted for immobility periods (for details, see ‘Bayesian decoding analysis’ in Methods).
(c) Session-level Bayesian decoding errors during awake immobility (related to Fig. 2g) using different combinations of training-testing behavioral windows (n=22 sessions). Labels indicate the behavioral periods used for training (top) and testing (bottom). Errors are z-scored with respect to a null distribution (see Methods). Trained run, tested run, p = 2.15e-5; trained run, tested active immobility, p = 1.02e-3; trained run, tested quiet immobility, p = 1.06e-2; trained active immobility, tested run, p = 4.89e-05;

trained quiet immobility, tested run, p = 2.01e-4; Bonferroni-corrected α=0.01, one-tailed Wilcoxon signed-rank test. Active vs quiet immobility encoding models, p = 1.36e-2, one-tailed Wilcoxon signed-rank test. * p<0.05, ** p<0.01, *** p<0.001

(d) 'Neuron dropping' procedure for active (left) and quiet immobility (right) encoding models, relating the behavioral modulation strength (peak of cross-correlation between instantaneous firing rate and whisker-pad motion) to the increase in the decoding error (y axis, Δ Error) caused by the removal of a single cell from the training set (for details, see 'Bayesian decoding analysis' in Methods). Active immobility, R^2 = 5.47e-2, $F_{(73,71)}$ = 4.11, p = 4.64e-2; quiet immobility, R^2 = 1.13e-2, $F_{(73,71)}$ = 0.81, p = 0.37.

(e) Session-level quantification of place-cell firing upon the effect of behavioral state (left) and of location (right) (related to Fig. 2f). Left, firing rates during active and quiet immobility 'in field' (p = 2.68e-4) and 'out of field' (p = 0.62). Right, 'in field' and 'out of field' firing rates during active (p = 1.33e-5) and quiet immobility (p = 7.84e-5). Bonferroni-corrected α=0.0125, n = 26 sessions, two-tailed Wilcoxon signed-rank test. ** p<0.01, *** p<0.001. Source data for this figure are provided as a Source Data file.

Supplementary Fig. 3

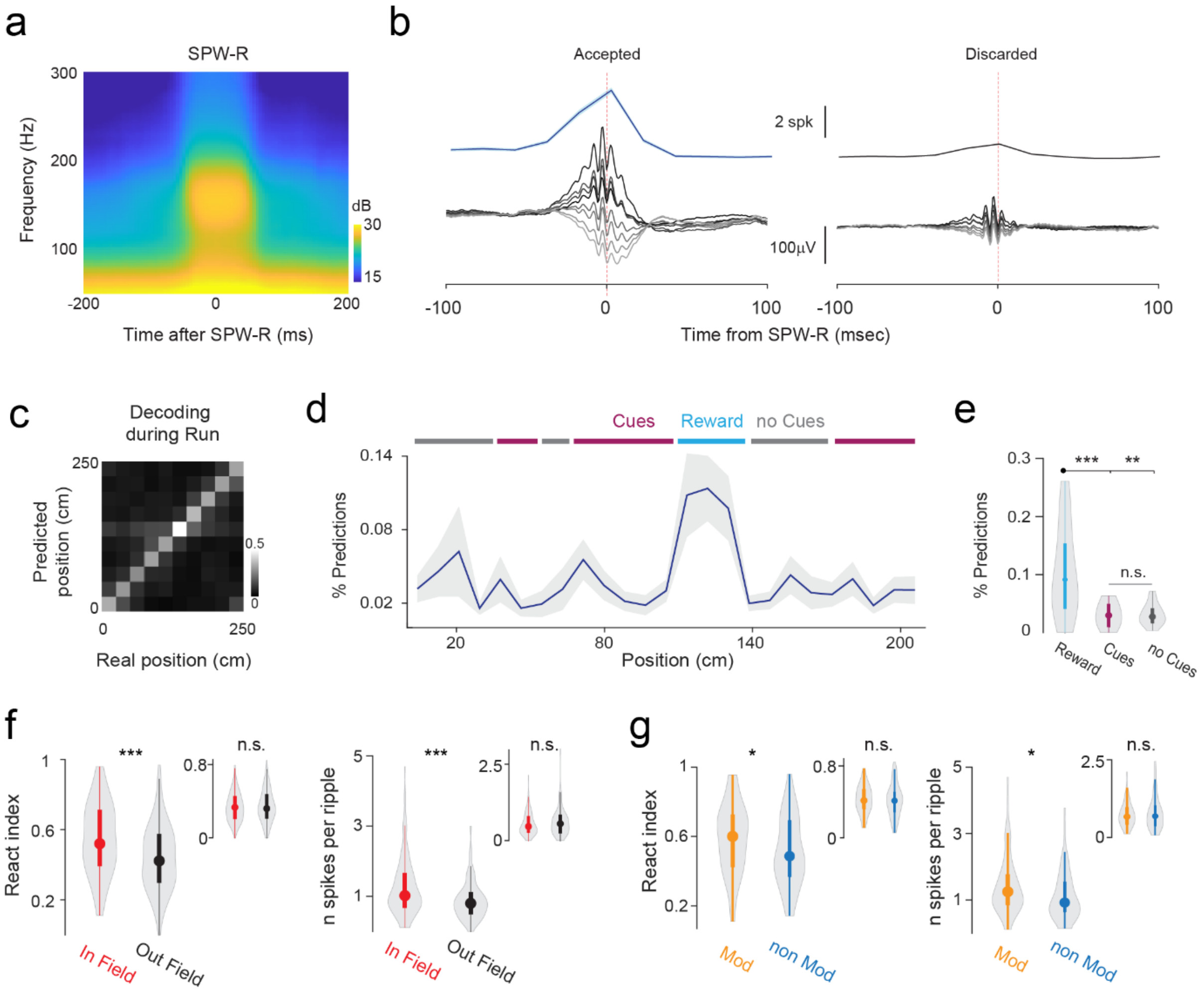

Supplementary Fig. 3. Sharp-wave ripple detection and neuronal reactivations.

(a) Average spectrogram for detected sharp-wave ripples in a representative session (SPW-R; n=978). **(b)** Representative perievent averages of accepted and discarded SPW-R from a representative session (see Methods). Top, average spike counts around accepted (blue, n=302) and discarded (black, n=1000) SPW-R. Data are shown as mean ± SEM. Bottom, average LFP traces across single recording sites for accepted (left) and discarded (right) SPW-R. Color shading indicates the depth across the CA1 (black, stratum oriens; light gray, stratum radiatum).

(c) Average confusion matrix showing the decoding accuracy during running epochs (n=19 sessions). The same decoders were used to reconstruct the offline location during SPW-R related reactivations (shown in d) (see 'Bayesian decoding analysis' in Methods).

(d) Average distribution of decoded locations during SPW-R-coupled reactivations (n=19 sessions). Top panel indicates different sections of the belt (blue, reward; purple, cues; grey, no cues). Error bars indicate SEM.

(e) Percentage of decoded locations quantified in different sections of the belt (same color code as in (d), n=19 sessions). p=2.00e-4, Kruskal-Wallis test; ** $p<0.01$, *** $p<0.001$, post-hoc comparisons.

(f) Reactivation index (left, see Methods) and the average number of spikes per SPW-R (right) during quiet immobility periods in field (n=139 cells, red) and out of field (n=139 cells, black). *** $p<0.001$, two-tailed Wilcoxon rank-sum test. Insets show the same analysis after spike down-sampling, where the firing in the field is matched to the firing out of the field. No Bonferroni correction applied.

(g) Reactivation index (left, see Methods) and the average number of spikes per SPW-R (right) for modulated cells (orange, n=69 cells) and non-modulated cells (blue, n=70 cells). * $p<0.05$, two-sided Wilcoxon rank-sum test. Insets show the same analysis after spike down-sampling, where the in-field firing of behaviorally-modulated cells is matched to the in-field firing of non-modulated cells. No Bonferroni correction applied. Source data for this figure are provided as a Source Data file.

Supplementary Fig. 4

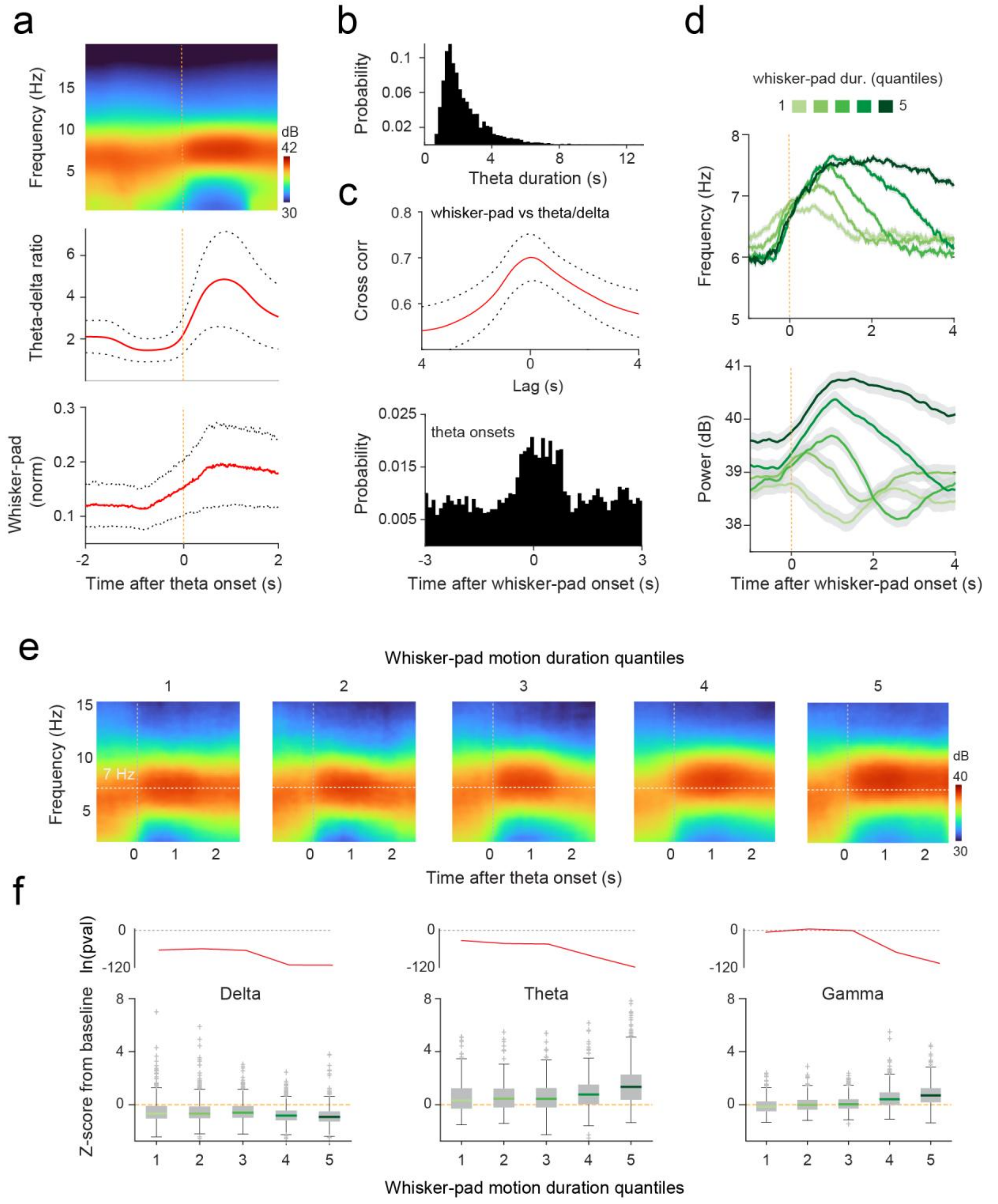

Supplementary Fig. 4. Theta oscillations detection and modulation by whisker-pad motion duration.

(a) Validation of theta segmentation. Top, session-average triggered spectrogram aligned to detected theta onsets (n=32 sessions); middle, average theta/delta ratio; bottom, average whisker-pad motion. Red lines indicate means, error bars indicate SD.

(b) Distribution of the duration of theta epochs during awake immobility (n=32 sessions, n=4288 theta epochs).

(c) Temporal relationship between theta and whisker-pad motion during awake immobility. Top, average cross-correlation between theta/delta ratio and whisker-pad motion (n=32 sessions). Bottom, peri-event distribution of theta onsets around whisker-pad motion onsets (n=4288 theta onsets, n=5886 whisker-pad motion onsets; n=32 sessions). Lines indicate means, error bars indicate SD.

(d) Time-resolved changes in frequency (top) and power (bottom) of theta oscillations during awake immobility aligned at the onset of whisker-pad motion. Data is grouped in five quantiles based on whisker-pad motion duration. Only whisker-pad motion events associated with theta are included (for details, see Methods, n=2277, events). Lines indicate means, shadows indicate SEM.

(e) Average triggered spectrogram aligned to theta onsets for different whisker-pad motion duration quantiles (n=2177 events, see Methods). White dotted line at 7Hz is indicated for visual reference.

(f) Quantification of delta (left, 0.5-4Hz), theta (middle, 6-10 Hz) and gamma (right, 70-90 Hz) bands in spectrograms presented in (e). Top, ln p-values across whisker-pad motion duration quantiles (Wilcoxon sign-rank tests); Bonferroni-corrected α=0.01 is shown as a dotted line. Bottom, boxplots of changes in power from baseline (1s before onset) across quantiles for delta, theta and gamma frequency bands (n=2177 events, see Methods). Color code as in (d). Source data for this figure are provided as a Source Data file.

Supplementary Fig. 5

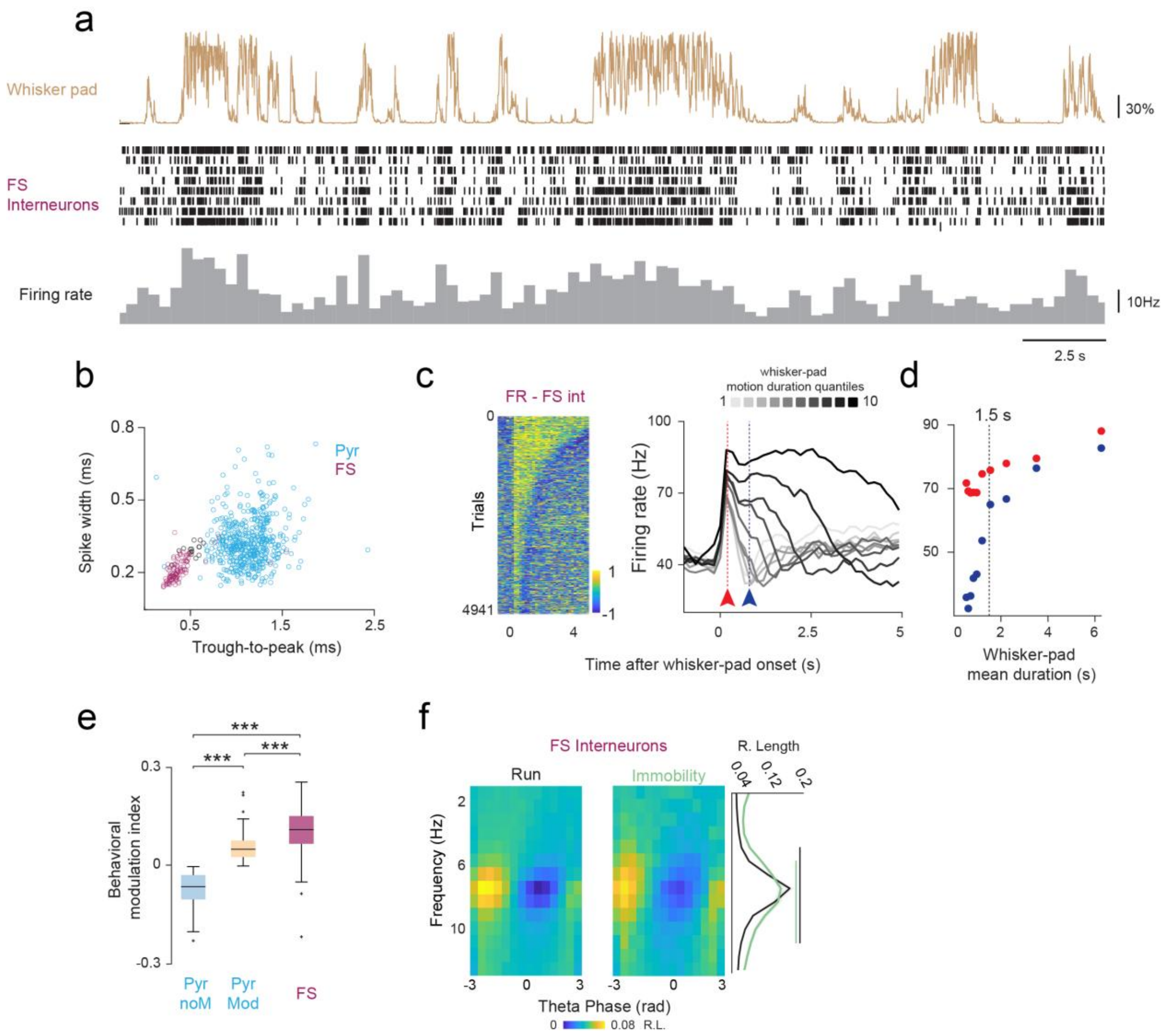

Supplementary Fig. 5. Behavioral modulation of putative FS interneurons during immobility.
(a) Example epoch during immobility showing interneuron firing (FS interneurons). From top to bottom: Whisker-pad motion (light brown); raster plot of putative FS interneurons recorded during the session; population firing rate (gray).
(b) Unit classification. Scatter plot showing the trough-to-peak distance versus the spike-width for all single isolated units (see ‘Spike sorting and unit classification’ section of Methods). Clusters separation was implemented by fitting a 2D Gaussian mixture model (see Methods). Putative interneurons are shown in purple, putative pyramidal neurons in light blue, and unclassified units in black.
(c) Left, peri-stimulus time histogram of the firing of putative FS interneurons aligned to the whisker-pad motion onset. Events are sorted by whisker-pad motion duration and z-scored with reference to pre-whisker-pad motion period. Right, average PSTHs of the same data divided in 10 quantiles of whisker-pad motion duration. Dotted lines and arrowheads mark the time points used for data quantification shown in (d).
(d) Quantile group averages at the two time points indicated in panel (c). Note that FS interneurons exhibited a “phasic” component (red), which remained relatively invariant across whisker-pad motion durations, followed by a second component (blue) that increased as a function of whisker-pad motion duration (a transition from a steeper to a more gradual increase is apparent at ~1.5 s, indicated by the dotted line, after which the firing-rate increase becomes more gradual).

(e) Behavioral Modulation Indices for non-modulated pyramidal cells (‘Pyr noM’, blue, n=85 cells), modulated pyramidal cells (‘Pyr Mod’, orange, n=81 cells) and putative interneurons (‘FS’, purple, n=105 cells) (see Methods and Fig. 3f-h). p=2.61e-39, Kruskal-Wallis test; post-hoc test: *** p<0.001.
(f) Theta-phase analysis of interneuronal spikes during running and immobility. Left panel, color-coded distribution of population phase locking as a function of theta frequency for run periods (color range: from 0 to 1, see Methods); middle panel, same analysis for immobility periods; right panel, quantification of theta-frequency modulation (Raleigh vector length, R.L.) for run (black) and immobility (green). n=27 sessions. Color bars indicate significant regions shared across 75% of the sessions (p<0.001, Rayleigh test, Bonferroni corrected). Source data for this figure are provided as a Source Data file.

Supplementary Fig. 6

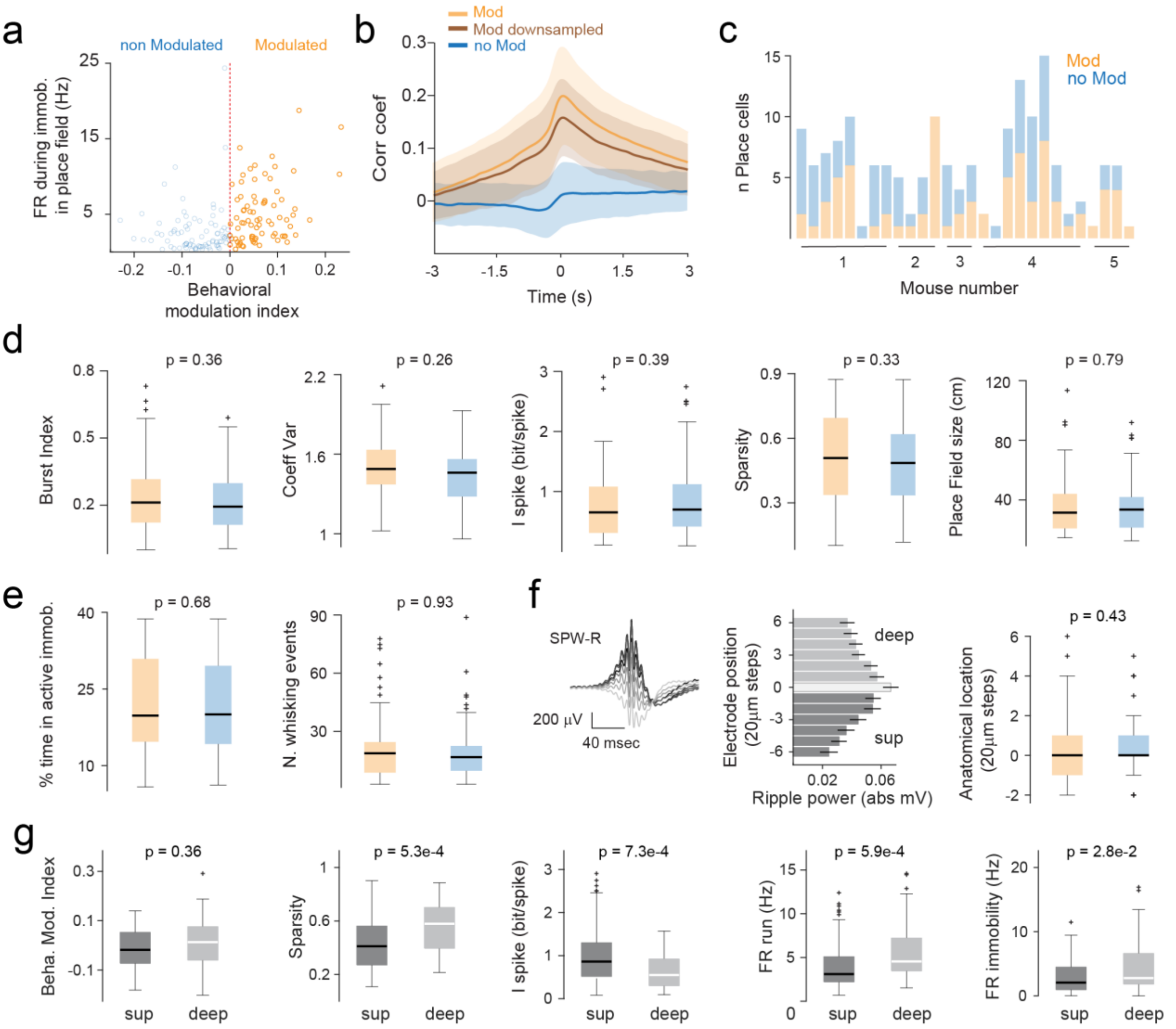

Supplementary Fig. 6. Electrophysiological properties of behaviorally-modulated and non-modulated place cells.

(a) Distribution of the Behavioral Modulation Index (see Methods) versus firing rates during immobility periods in field, for behaviorally-modulated (blue, n=81) and non-modulated cells (orange, n=85).

(b) Average cross-correlograms (instantaneous firing rate versus whisker-pad motion, see Methods and Fig. 5e) for behaviorally-modulated cells (blue), modulated cells with downsampled spikes (brown, see Methods) and non-modulated cells (orange). Lines indicate means, shadows indicate SD. n as in (a).

(c) Distribution of behaviorally-modulated and non-modulated place cells across individual sessions and subjects. n=28 sessions, color code as in (a).

(d) Electrophysiological properties of behaviorally-modulated and non-modulated cells. From left to right: burst index, coefficient of variation, spatial information (I_{spike}), sparsity and place field size (see Methods). n and color code as in (a). p values are indicated (two-sided Wilcoxon rank-sum test). P-values were not corrected for multiple comparisons.

(e) Relative time spent in active immobility (left) and number of whisker-pad motion events during awake immobility (right) for behaviorally-modulated and non-modulated cells. n and color code as in (a). p values are indicated (two-sided Wilcoxon rank-sum test).

(f) Anatomical localization analysis. Left, average SPW-R across all sessions (n=30 sessions), color coded by the depth of recording sites (more dorsal, blacker). Middle, average distribution of ripple

power across recording sites. The channel with largest ripple power was taken to be the closest to the pyramidal layer and used as a reference to group units along the radial axis into deep (light gray) and superficial (dark gray) classes. Mean ± SEM are indicated. Right, relative anatomical location of behaviorally-modulated and non-modulated cells. Zero indicates the highest ripple power along the recording sites; positive means deeper (i.e. towards the stratum radiatum). n=57 modulated; n=60 non-modulated cells. Color code as in (a). P value is indicated (two-sided Wilcoxon rank-sum test).
(g) Electrophysiological properties of deep and superficial place cells. From left to right: Behavioral Modulation Index, sparsity, spatial information (I_{spike}), firing rate during running, firing rate during immobility. n and color code as in (f). P values are indicated (not corrected for multiple comparisons, two-sided Wilcoxon rank-sum test).
