# Supplementary material for: Place and behavioral modulation of hippocampal neurons during immobility
Source: Nat Commun. 2026 Jul 24;17:7261. doi: 10.1038/s41467-026-75492-w (PMC13400744; doi:10.1038/s41467-026-75492-w)
Supplement: Supplementary file 3 — Transparent Peer Review file [file 41467_2026_75492_MOESM3_ESM.pdf]

nature portfolio

Peer Review File

Place and behavioral modulation of hippocampal neurons during immobility

Corresponding Author: Professor Andrea Burgalossi

This file contains all reviewer reports in order by version, followed by all author rebuttals in order by version.

Attachments originally included by the reviewers as part of their assessment can be found at the end of this file.

Version 0:

Reviewer comments:

Reviewer #1

(Remarks to the Author)
This study provides insights into the modulation of activity in place cells and accompanying oscillatory dynamics in the dorsal ca1 region of the hippocampus. The authors simultaneously captured facial expressions and online brain activity, while mice were engaged in space clamped vs. non-clamped experiments on a belt. By aligning traces of facial movement, local field potential of the region and single unit analyses, authors provide evidence that place cell like activity and theta oscillations emerge in some cases in which the mouse is not in locomotion. Specifically - this activity is related to the arousal level of the animal, as measured by pupil dilation and engagement of the mouse in whisking behavior. The authors propose a layer of encoding mechanism that incorporates arousal level in a multiplexed model of episodic memory processing. This behavioral-state related modulation in the brain regions can potentially support curation of episodic memory content and has implications in understanding the nature of hippocampal outputs during encoding and consolidation of such memories.

While the study is of interest, we feel that additional work has to be done in order to incorporate the results into the known body of literature related to place cells and their function, and the relation between the place cells and the local field potential. Also, more effort should be done to explain what is new here relative to previous findings.

MAJOR ISSUES

1. The authors separate in the paper two populations of cells, one which is behaviorally modulated why whisking and arousal, and a second that is not. This a very strong claim. In order to make it, the authors should present some sort of a bi-modality. We suspect that their cells are part of a continuum, and thus it is hard to state that these are two populations. Furthermore, In the example in Figure 4b, it seems that the increase in firing of the non-modulated cell occurs at the offset of the whisking. Could this be the case in general for the non-modulated cells?
2. The second issue related to this is that cells in CA1 can remap, and thus the results should be interpreted in relation to remapping. It is more probable that the non-modulated group are actually cells belonging to a different map, and not an anatomically-distinct group, as the authors suggest in the discussion.
3. The fact that place cells continue to fire at high levels during immobility if the mouse is within the place field of the cells suggests that the cells are still mapping space. The authors should attempt to run a decoder analysis with respect to the spatial map during immobile states.
4. As the authors are dealing with a situation in which the mouse is undergoing different behavioral states, they should attempt to cluster those states and look at the behavior of the network, similar to Rubin, Sheintuch et al., 2019.
5. Phase precession is reported in place cells with respect to ongoing Theta. Authors segmented the experiment in running, inactive immobility and active immobility epochs. Presence of theta band in the LFP during running and active immobility calls for phase precession related analyses might aid in differentiating network status during these behaviorally different epochs.
6. The results in the paper may be related to the time cells literature (e.g. McDonald…Eichenbaum, 2011), and thus analysis for checking if there is phase coding relative to the onset and offset of the whisking behavior should be performed.
7. We are missing a systematic quantification and statistics of the abudance of theta and other oscillations in different behavioral states, and as a function of arousal level. What was the level of coherence between theta LFP and whisking

cycles?
8. Many of the points in the discussion are very speculative. Some examples of unfounded claims:
“indicating that the gain-modulation observed at the population level (Fig. 2b-d) is primarily driven by a subset of behaviorally-modulated neurons”
“The subset of behaviorally-modulated place cells might inherit modulation via direct (or indirect) inputs from the brainstem arousal centers, from the CA2 or from the upstream head-direction system”
“behaviorally-modulated and non-modulated place cells might correspond to different subtypes of pyramidal neurons”
“This evidence suggests that behavioral state fluctuations set different theta oscillatory regimes in the hippocampus, and these effects are likely mediated by local FS interneuronal networks”
“we speculate that rearing might correspond 300 to a higher-arousal state than locomotion”.
Please compress the discussion leaving most of these speculations outside.

MINOR ISSUES

9. Authors mention the ethological significance of intermittent pauses during naturalistic behavior in their discussions, but random pauses in their experiment, seem to be experimentally controlled (Sup Fig1b) to achieve pauses across multiple place fields. Can they add a comment, if an attempt was made to distinguish active pauses with failed attempts to move?

10. Can authors add a line, about the rationale of switching illumination status of the experimental setup between active and immobile periods?

11. Can authors add a line, explaining the rationale of the auditory pulse, preceding juxtacellular stimulus to induce a place field? What was the reason to believe that place cell was due to injected current but not due to behavior or sound stimulus ?
12. The division into short, medium and long events is a slightly arbitrary analysis – better to do regression.
13. Figure 4d, assumes only immobility, please seprate into active vs. inactive immobility.
14. Language issue: footnote for sup 3c has an English problem.
15. Please add units for X axis in Figure 4e (Hz ?).
16. What was the relation between the cells activity and the reward? Could it have affected the results?

Reviewer #2

(Remarks to the Author)
Comments to the authors: This study from Sartorato et al. shows that place fields in dorsal CA1 are somewhat intact (reduced peak firing rates) during periods of imposed immobility. The immobility place fields are more pronounced in about 50% of the CA1 place cells during Active immobility states, as defined by whisker pad movement and pupil dilation. Longer durations of Active immobility accelerate theta rhythms, increase relative theta power, and enhance entrainment of FS putative interneurons to theta. While these findings are both interesting and novel, the analysis is rough, at times incomplete, and at other times, regrettably, incorrect. There are major revisions necessary to publish this manuscript.

My major concerns are:

1.) "Whisking" in mice is a sampling behavior where the angular position of the entire whisker array oscillates at 10–20 Hz, creating rhythmic protraction/retraction around a slowly varying set point. The author's claim that mice are "whisking" (line 78 and many other places in the manuscript) is not valid because it is simply based on a facial motion energy measure. Whisker pad motion during running could follow other, slower, rhythms (like breathing) or it could be uncoordinated and unique to locomotion. To show that the mice are "whisking" while they run, the authors need to perform a more standard analysis looking at the frequencies and correlations of whisker-pad-wide movements. I am not sure that this is possible in their recording configuration (video of face and not top view of whiskers). That being said, I think they could also just not use the word "whisking", but rather a behavioral state comprised of immobility and high whisker pad motion. The whisker motion during running is not crucial because it is not variable and they do not use it to define any behavior states.

2.) The statistics are not correctly done and some controls are lacking. It is never clear how many co-recorded neurons are being presented (how many cells per mouse per recording etc.). Co-recorded cells are not independent and thus should not be tested as independent samples (N should not be number of neurons, but rather number of recordings except for the juxtacellular case where 1 neuron was recorded at a time). In Figure 1 there are no stats, in Figure 2 stats are on Ncells, in Figure 3 stats are done on arbitrary "duration" groups with different window sizes and counts (see below for more precise comments), and in Figure 4, the modulation index is incomplete, the firing rate analysis is statistically circular (see below) and the claims about ripples are not statistically grounded. It is therefore difficult to judge if the results of the paper will stand up to more rigorous statistical assessment. I find it strange that 2 specific claims in the abstract (in brackets below, see further below for detailed explanations) are only featured in supplemental figures, and are based on very shaky statistical or experimental evidence: "The hippocampal spatial code was preferentially contributed by a subset of behaviorally-modulated place-cells, which increased their firing upon transitions to active immobility and [were preferentially recruited during awake ripple oscillations]. [Single cell stimulation was sufficient for the induction of place fields], indicating that plasticity mechanisms can be engaged even in the absence of locomotion."

3.) The presentation of the data is incomplete and sometimes obscure. In Figure 2, the authors segregate immobility periods into active and quiet. In figure 3, they only compare active immobility and running, leaving the reader wondering what happens during quiet immobility for the same measures. Then in Figure 4, they start looking at general immobility (both

active and passive pooled, panels d and e), only to statistically quantify active vs quiet immobility (panel g). There are three behavioral states defined starting in Figure 2: Run, Active immobile, Quiet immobile… and I do not see why all behavioral states are not quantified together for all figures. It seems suspicious that they are not all treated in the same way for statistics. Even when normalizing to the peak of Run place fields, we should see the Run curve on the plot because the sharpness of the place fields might be different and interesting in and of itself.

4.) There is no population level analysis in the paper. What do these different populations of cells tell us during the different behavioral states? How good is the place decoding in these cell populations (behaviorally modulated and non-modulated) during Run, Active immobile, and Passive immobile ? How well can we predict whisker movement energy just by seeing the population activity ? The paper is thin on experiments and this axis could help to beef the analysis up.

Here are some more detailed comments on the Figures:

Figure 1:
There is no legend for the heatmap in panel c.

In the example shown in Figure 1b, there are obvious moments in the "Run" periods where the animal transiently halts locomotion, moves its whisker pad, constricts its pupils, and LFP power is more spread… so why are they included in "Run"? I suppose these events were too short to be called immobility but that does not mean they should automatically be called Run, as the animal is not running.

"During immobility, LFP activity was more heterogeneous, characterized by low-frequency activity as well as bouts of theta epochs (Fig. 1d)…"
Epochs or bouts are not evident in Figure 1d, which is a power spectrum so does not show "bouts" of anything. I do not see theta bouts either in Figure 1b, but maybe a zoom in on a particular bout could highlight what the authors refer to ? Also, I do not understand what heterogeneous LFP means. There are more pronounced peaks in the Run data for low freqs, theta, and beta/sigma, as well as higher gamma in general… whereas the power spectrum is smoother for immobility. I am not sure that "smoothness" is the same as heterogeneity.

Figure 2:
"…phase being characterized by immobility-related LFP theta activity (Fig. 2b). "
This graph is an inset in Figure 2a…

"On the other hand, sharp-wave ripples were exclusively observed during ‘quiet’ immobility (Fig. 2 and Extended Data Fig. 2), thus further supporting the binary brain state classification."
This is not supported by the data presented. In Figure 2 itself, there is a filtered LFP in the ripple band (150-250) Hz that shows a single SWR that occurred in the "Quiet" state, not sufficient evidence to support this claim. In the Extended data Fig. 2, the SWR rates in "Active immobility" state are not 0, and further, the time when the cells show the highest levels of spiking at the start of the "Active" behavioral state, SWRs are not measured due to artifacts. The authors need to either change this statement or show an analysis that supports their claim that ripples occur exclusively during "Quiet" immobility.

Panel 2b the heatmaps do not highlight the potential differences. The warmth scale is too broad and the differences are not easy to appreciate, also the frequency range is much broader than it needs to be (theta/delta seem to be the important ones). Consider altering the scale and/or creating a third "difference" heatmap. Theta/Delta score is never statistically quantified so we are left wondering if these differences are significant.

"To explore whether CA1 place coding is preserved during periods of immobility, we analyzed all immobility periods within and outside the cells’ place fields (n=166 place cells in field, n=164 place cells out of field). We found that on average, place cells fired at higher rates when animals were kept stationary within the cell’s place field, as compared to outside, irrespectively of the behavioral state (firing rates normalized to run periods; active immobility: in field, 0.38 ± 0.29, out of field, 0.18 ± 0.21, post-hoc comparison p=1.02e-16; quiet immobility: in field, 0.24 ± 0.17, out of field, 0.18 ± 0.16, post-hoc comparison p=8.67e-4; p=4.23e-19, Kruskal-Wallis test; Fig. 2c,d). Consistent with the behavioral modulation data (Fig. 2b), place cell activity was highest in the ‘active’ state (Fig. 2c,d)."
There were 340 place cells in Fig 1. What happened to them for this part where there are only 166? Panels c and d are not enough. The authors must show the full rate maps (like Fig 1c) for Run, Active immobile, and Quiet immobile, normalized to peak for all cells that this data exists for. Not sure if it is just misalignment in the figure, but the peaks of the immobility curves in Fig 2c are not aligned with the peak for the red curve above from the Run.

"In a subset of stimulated cells (8 out of 34), evoking spiking during active immobility was sufficient to create a de-novo place field at the stimulus location (Extended Data Fig. 3) "
This experiment lacks a control. The sound that is used to encourage active immobility is a cue that itself could induce plasticity or activity. The authors need to perform sound cue only controls without micro stimulation. This will answer 2 questions, does the sound evoke activity itself? Does the microstim drive the plasticity and is this plasticity more than we see just with the sound? (8/34 is infrequent and maybe this can be explained simply by an unfamiliar sound cue at a particular location)

Figure 3:
"We found that, compared to running, individual theta bouts during immobility showed a larger variability in frequency and reduced power "

It is not clear why the authors are calling these "theta" bouts. They are 1-second periods after the onset of Active whisker pad movements during immobility. That is not a "theta" bout per se, as it is defined by behavior that may or may not be associated with high or low theta (not properly quantified either in Fig. 2b). To be a "theta" bout, it would be a period of high theta activity that is detected based on theta power… The authors specify that running "theta" bouts were sampled uniformly from mobility periods, and this could be incorrect. The authors need to verify that Active immobility is uniformly distributed within immobility periods, otherwise their sampling should match whatever distribution this yields. Active immobility periods might be clustered together or they might be biased towards onset or offset of immobility. The standard approach to this is to match inter-event-intervals (maintain the IEIs but shifted during the mobility periods) instead of choosing times from a uniform distribution.

For panels c-h, the authors divide the dataset into 3 arbitrary groups of Active whisker movement durations of unequal sizes. In general, arbitrary divisions as such can be useful for visualization but not for statistics. They should not base their statistics on these groups but rather on the correlations between duration and the various measures. They should also standardize these measures so they can be applied to all durations in the same way (variable window sizes is not good). They make two claims that need clarification and better analyses:

1.) "We found that the duration of whisker-pad motion events correlated with the magnitude of pupil dilations (Fig. 3c) and was hence predictive of the arousal level of the animal ".
Here, the shorter Active whisker movement events are perfectly aligned with the longer events in terms of pupil dilation (see 3c bottom, before 1 second), and the dilation only separates once the shorter events have finished. So, pupil dilation follows the same trends in all three duration groups. For longer durations, there is more time to dilate … I would conclude that arousal as measured by pupil dilation builds with time spent actively immobile, with active immobility defined by whisker movements. I find the authors' claim stated above imprecise… it does not specify that this effect builds over time, as their text only refers to magnitude without considering the extended time window associated with longer active immobility events.

2.) "Interestingly, the frequency and relative power of theta oscillations also correlated with these behavioral metrics, with theta oscillations becoming progressively faster as a function of the duration of spontaneous whisking events "
Here, they are comparing LFP power spectra from time windows of different sizes. They should redo this analysis at the spectrogram level to try and understand the dynamics of the theta power across Active immobility periods. If the theta frequencies are already faster in the early parts of the event and predict a longer active period, that is different (and perhaps more powerful) than if the theta frequencies accelerate as active whisker movements perpetuate. They could look at the first 0.5 seconds and the last 0.5 seconds of the spectrograms to see this, or only compare the last 0.5 seconds across all events. They could also quantify how long it takes the pupil to stabilize… something they refer to as "steady state" of 1.5 seconds in the methods but never explain or justify.

Figure 4:
The "modulation" index is not correctly defined. Modulation can be either positive or negative. The authors only use the upper boundary of the confidence interval which suggests they only looked for cells that are positively correlated with Active whisker pad movement. In Fig. 4e, it is shown that some neurons have negative correlations at peak, so for any "modulation" index, the statistical test should be extended to see if these cells are significantly anti-correlated with the whisker movements. This will also fix the problem in Figure 4c that z scores are only positive… which is because they ignore the negatively modulated cells (however they seem to be fewer and less strongly modulated).

" However, during immobility, modulated neurons tended to fire at higher rates (average firing rates in field during awake immobility; modulated, 4.96 ± 3.88 Hz; non-modulated, 2.73 ± 3.34 Hz; p=1.04e-06, Wilcoxon rank-sum test; Fig. 4d) and to be preferentially recruited during awake sharp-wave ripple oscillations (Extended Data Fig. 2)"
The modulated neurons were defined as having a higher than chance cross-correlation (a measure that is sensitive to firing rate) with whisker movements during Active immobility… then they are re-compared with non-modulated neurons that did not have significant modulation during Active immobility… but this time the comparison is during general immobility… this is circular statistics so not a valid test. The authors could compare the firing rates during quiet immobility because whisker movements (which were already tested for) cannot bias the results. For SWRs, the authors have to do the above test properly. If the modulated neurons do have higher firing rates during quiet immobility as well, we would expect them to be slightly more active during quiet immobility ripples as well and this should be boiled into the test (which was not done) to see if they are indeed more recruited during ripples beyond what we would expect based on higher firing rates in general.

"Notably, during active immobility, the hippocampal spatial representation was almost exclusively contributed by the subset of behaviorally-modulated place cells (Fig. 4f,g), indicating that the animal’s location is primarily represented by the ensemble of behaviorally-modulated place cells."
This is not shown. There is a larger bump for the "modulated" cells on the left in Fig 4f… but the place fields are still visible for quiet immobility and also for active/quiet immobility for non-modulated cells. Also, the between group comparisons in 4g just show that modulated neurons have higher firing rates during active immobility than all other cells/conditions. Again, not showing that the spatial representation "almost exclusively" is comprised of these cells. To show this, the authors have to use population approaches on spatial decoding and drop out the different populations (modulate/non-modulated). The spatial information could still be very well intact in all conditions.

Minor corrections:

There are many grammatical errors and not all are listed here …

Some early suggestions:
Abstract: This sentence has a grammatical error (parenthesis) and repetition problems (bracket): "The hippocampal spatial code was [preferentially] (contributed by) a subset of behaviorally-modulated place-cells, which increased their firing upon transitions to active immobility and were [preferentially] recruited during awake ripple oscillations."

contributed by is used again in the main text of the manuscript and this does not work in the context it is used. Maybe use "carried by" ? (if the analysis that was suggested indicates this is true)

Line 5: … the phenomenology … has been …
Line 20 : … in correspondence with …
Line 49 : … to hippocampal dynamics …
Line 51 : … place cell activity…

I stopped here and would suggest the authors get the manuscript proofread before resubmission.

Reviewer #3

(Remarks to the Author)
Summary
Sartorato et al. capitalize on an experimental setup which allows "space-clamping" in order to study how hippocampal representations are modulated by behavioral state when the mouse is immobile. This question has remained quite understudied in the field, and is important in our understanding of hippocampal coding during immobility. Here, the authors use camera footage of the mouse's face to classify periods of behavioral arousal while the mouse is immobilized (ie. periods of active whisking). The authors find that place cells increase their firing rate during active whisking, an effect which is also accompanied by changes in theta power and frequency, suggesting that behavioral state drives distinct neural activity states in dCA1. Finally, the authors show that the increased cell activity during immobility emerges from roughly half of the recorded place cells whose firing rates are entrained to the whisking of the mice, indicating a distinct subpopulation of place cells that are behaviorally modulated. While the experimental question and approach are timely and interesting, the analysis presented here does not support the author's claims that "during awake immobility, the cognitive representation of location is active and dynamically modulated," as it focuses primarily on gross firing rate changes, which do not necessarily indicate a change in the cognitive representation of location. Furthermore, the results of the juxtacellular recordings described seem underwhelming and out of place, without further controls it is unclear what they tell us about the relationship between behavioral state and place coding. Therefore, the impact of this paper is unclear without additional analysis and experiments, which I outline below:

Major Concerns:
1. The primary claim of the paper is that behavioral state modulates the cognitive representation of location during immobility. I do not think the analyses presented here entirely support this claim. The authors show two main results to support this conclusion: 1) place cell firing rates increase with active whisking during immobility, and 2) this firing rate increase was only present in about half of the recorded place cells. The observation that firing rate increases does not tell us much about the cognitive representation of location. If anything, many of the results of this paper indicate that place cells should maintain a fairly stable representation of location during immobility, regardless of behavioral state (for instance, the tuning curve in Fig 2C is only slightly changed, and standard place-coding properties are not changed between modulated and unmodulated cells such as place field size, bits/spike, and field sparsity in Extended Data Fig 6f,g; that said, there is clear gain modulation of place tuning in Fig. 4f). An analysis of the pseudo-place fields generated using the space clamping technique (Fig. 4f) would provide much more insight into the nature of spatial tuning during immobility. For instance, how the size, spatial information, and sparsity of the pseudo-place field are affected by behavioral state. Furthermore, to claim that the cognitive representation is modulated, I would be more convinced by Bayesian population decoding during the immobile state: Would decoding accuracy increase when the mouse is actively whisking while immobilized (as might be predicted by the tuning curve in Fig. 4f)? If so, what is causing the change in accuracy? Is the posterior of the decoder sharper or are there fewer non-local events that are common during immobilization (as suggested by Extended Data Fig. 2c). In the current draft, very few analyses seem to directly quantify the representation of location (namely, how precise is the place code during immobility, and how is it affected by behavioral state).
2. The authors frequently mention gain modulation of place cell firing, but never actually quantify the gain changes within the tuning curves (at least beyond gross firing rate changes, which imply an additive effect that is not necessarily related to "gain" which I typically associate with a multiplicative effect – I bring up this point because an additive shift of the place field would not change the spatial information in the place field, only a multiplicative "sharpening" of the field would increase spatial information). To do so would be relatively easy: for instance, for Figure 2c, per cell, plot each point of the quiet curve against each point of the active curve and fit with a line: the slope would be the gain value and the intercept would be the additive firing rate shift. A similar analysis would likely reveal a large multiplicative gain change in modulated cells in Fig. 4f.
3. Generally, the authors emphasize the SWR results, but it is unclear why they are relegated to a supplement. These results are interesting, and further exploration of decoded position during SWRs would provide further support that the cognitive representation of location is modulated by behavioral state. While authors observed a decrease in ripple rate during active immobility, they also saw that the behaviorally modulated place cells were more active and more likely to be reactivated during ripples (Extended Data Fig. 2g). These effects are interesting, and while this may be an artifact of having higher firing rates, they raise a lot of questions: Are there more non-local sequence events (see work from David Foster and Loren Frank) during active versus quiet immobile states (ie. forward/reverse replay)? Are the non-local events more likely to contain the

modulated ensemble of cells? If so, are the non-local events preferentially representing particular locations, or particular types of replay (say, forwards versus backwards replay, or sequences extending ahead of or behind the mouse, which might suggest a role of behavioral state in consolidation versus prediction).
4. The result of an increase in theta power does not quite line up with the spectrograms in Fig. 2b. By eye, it looks like there is more low theta power in the quiet state than in the active state (dark red band near 5Hz). While I understand the quantification is using the theta/delta ratio, the delta ranges seem to include some of the typical theta range (in the discussion, Type-2 theta is defined from 4-7Hz, while the delta range used for the ratio overlaps with this – from 1-5Hz in the methods; previous studies also use delta ranges closer to 2-4Hz, see Malezieux et al., 2020; Grosmark et al., 2012; Wikenheiser and Redish, 2014). Given that the main effect appears so close to 5Hz, do these results hold using typically defined ranges for theta and delta?
5. The juxtacellular recording results are somewhat underwhelming and difficult to interpret without further control experiments. While they do demonstrate that it is possible to induce a place field during active immobility, without further context this does not say anything about behavioral state modulation of the hippocampal code. For instance, is it equally likely to cause place field formation during quiet immobility (the same experiment without the auditory tone)? Is stimulation more effective in behaviorally modulated cells during active immobility (as far as I can tell, it would be feasible to perform the cross-correlation analysis used in Figure 4 to determine whether the stimulated cell is behaviorally modulated)? The place field induction rate here is quite low (8/34) compared to the same experiment during running (14/15 cells in Bittner et al., 2015), which would suggest that, if anything, an immobile but active state reduces plasticity. As presented here, it is hard to understand what this result implies without some of the controls mentioned (ie. replicating Bittner et al during running, or comparing to induction success during the quiet immobile state – the latter seems like the ideal control in this case).
6. It is generally unclear how the space-clamp is implemented without digging into the methods. Because this is the key experimental manipulation in this paper, it is important to make this technique entirely clear. It should be explicitly explained in the main text that the mice are forcibly immobilized by applying a brake to the treadmill. I would also suggest a graphic that describes this in Fig. 1a.
7. The authors should show histological traces of probes in hippocampus to verify the recording sites in dCA1.

Minor Concerns:
1. Possible grammar issue in the abstract. The sentence “the hippocampal spatial code was preferentially contributed by a subset…” sounds incorrect.
2. In Figure 1d, middle, why is the peak of the running trace shifted off of zero if the data is z-scored to the running trace? Shouldn’t that peak fall exactly on zero?
Line 100: n=166 place cells, but it is unclear how these cells were selected from the 340 place cells?
3. Fig 2c: n=131 cells, how were these selected from 166 cells referenced in the section of text (which says n=166 or n=164, then cites Fig. 2c at line 112)? Are the error bars computed over cells or immobility periods?
4. Line 282: In the discussion, the authors say that theta oscillations occurred in short bouts, citing Figure 2a and Extended Data Figure 4, but “bout length” is never quantified or analyzed. This statement should be backed up by an analysis or reworded as a qualitative observation of the data.
5. Extended Data Figure 2b, in the caption the discarded traces are said to be orange but they are black.

Reviewer #4

(Remarks to the Author)
I co-reviewed this manuscript with one of the reviewers who provided the listed reports. This is part of the Nature Communications initiative to facilitate training in peer review and to provide appropriate recognition for Early Career Researchers who co-review manuscripts.

Version 1:

Reviewer comments:

Reviewer #1

(Remarks to the Author)
I have read the response to reviewer 1 and the common response to all reviewers and have also browsed through the paper and the response to all reviewers, and think the paper has substantially improved. I appreciate the well thought of and well written rebuttal, which has taken seriously all the comments written by the reviewers.

I have some minor comments left, that may be fixed towards publication by refining the Discussion:

Point number 2 - The fact that the second map was not revealed in your experiment does not mean it does not exist. Specifically, maps could flip between whisking and non-whisking states, such that the hippocampus toggles between two different maps belonging to two different contexts. The pre-post correlation does not answer this because it could be that there was one map before the stop, similar to the map after the stop, with a different map in-between. Maps could actually even flip quickly at a speed of one map per theta cycle (Jezek, Karel, Espen J. Henriksen, Alessandro Treves, Edvard I. Moser, and May-Britt Moser. "Theta-paced flickering between place-cell maps in the hippocampus." Nature 478, no. 7368 (2011): 246-249). The main point is that I do not expect the non-modulated group to be anatomically distinct from the modulated group. Things in CA1 are more dynamic than this.

Point number 5 - Reviewer figure 3 - Precession could be clearly seen also during immobility (although panel b is noisier). All you have to do is spatially smooth panel b and some precession will emerge.

These points are not essential and do not preclude publication, as the paper is really nice and all essential points have been answered.

Reviewer #2

(Remarks to the Author)
First, I would like to commend the authors. They put in a lot of work to improve their manuscript and it is much more polished now. The revisions have clarified which results are robust and which do not stand up to more rigorous statistical assessment. I have a few remaining issues, and pending the resolution of these issues, I support publication of the paper.

1) The authors argue in their rebuttal:
"The Reviewer raised a concern related to the computation of statistics using cells as individual units. While we acknowledge that this is, in principle, a valid concern, it is standard practice in hippocampal research to treat place cells as independent units (see, e.g., 3, 21–24). This assumption is supported by extensive evidence showing that individual cells map space independently of one another (see e.g. remapping, and the uncoordinated behavior of cell-pairs relative to one another; for a review, see 25,26). Moreover, the standard Bayesian decoders widely used in the hippocampal literature also rely on the assumption of independence of place cell firing given the animal's position 27–29."
Here, I don't think their reasoning is good, but only have a small requested change.
First, I agree with the authors that in the hippocampal literature, treating co-recorded place cells as independent samples is common. So, I can understand why they do it. Unfortunately, common practice doesn't always mean best practice, especially in different and new experimental conditions. I encourage the authors to inform themselves about some of the common pseudo-replication errors throughout neuroscience-related research fields and their implications (see for example "Better statistical reporting does not lead to statistical rigour: lessons from two decades of pseudo-replication in mouse-model studies of neurological disorders. Eleftheriou et al. 2025).
Second, they should be careful about what exactly is independent and whether that independence is the right kind of independence for their statistical tests. Even if "…individual cells map space independently of one another…", place cells are of course not only coding space (as the authors show in their manuscript, they encode behavioral state variables too). Moreover, network interactions explain a substantial amount of population activity in CA1 (see Collective Behavior of Place and Non-place Neurons in the Hippocampal Network, Meshulam et al. Neuron 2017), meaning even if place fields are independent, the general spiking activity of CA1 neurons is not. Many of the statistical tests in the manuscript are not about spatial coding alone, but rather they pertain to behavioral-modulation of spatial coding during immobility (Active vs Quiet). About half the cells are behaviorally modulated in a place-specific manner, but that does not mean that behavioral modulation is also independent in co-recorded cells (even if it is only visible at uncorrelated place field centers). Their results suggest that inputs that drive behavioral modulation target some cells more than others (not all cells display it), and depending on the session, we might have more or less cells more strongly targeted by these inputs. It is also possible that the inputs driving behavioral modulation themselves are more or less active in different sessions. Both of these possibilities mean that we might have within session correlations of behavioral modulation that are seen only at place field centers. Within session correlations would imply a lack of independence. Treating cells individually masks this co-variability, which could potentially lead to false positive ridden statistics (see again Eleftheriou et al. 2025 Fig. 3B-D).
For these two reasons, 1) the common practice cannot be blindly applied in new situations, and 2) when looking at behavioral modulations of spatial coding (Active vs Quiet, not spatial coding alone), independence is not established. I reiterate the need for session-based statistics even if it does not change the conclusions (great news if it does not). The authors do not need to move anything, and they can leave the figures, just refer the reader to session-based stats (when available and applicable, even if they are in supp figs) explicitly in the main text.

2) The authors use the concept of "excitability" 4 times in the manuscript (lines 133, 218, 220, and 298, but also many times in the rebuttal letter) to explain why firing rates of certain neurons are higher than other neurons. For me, changes in excitability is a borrowed concept from intracellular physiology that cannot be concluded here. In intracellular physiology, cells that are more "excitable" fire more readily for a fixed level of direct current injection. Here, in extracellular conditions where we only record spikes of large neuronal populations with no control over their synaptic input, we cannot know if the cells are receiving the same input levels to then conclude that one is more excitable than another. Some cells might receive inputs that other cells do not, so it might not be excitability, it could just be different signals that are integrated. I suggest they reword these 4 sentences to avoid using misleading or speculative language.

3) I still think some of the stats exhibited in Fig. 5h are circular, despite down-sampling or shuffling that was performed in the rebuttal. The authors separate cells into behaviorally modulated and non-modulated using a correlation-based (cross-corr peak between spiking activity and whisker motion during immobility) modulation index statistical test. This identifies cells that fire more during Active immobility. Then, they perform a second round of statistical tests in Fig. 5h on these cells to assess whether modulated vs non-modulated cells have different firing rates between active and quiet immobility. Stats between modulated and non-modulated populations are contrived by the classification they make in the first modulation index statistical test. Why this is (see atatached drawing for graphical description) … If I had any distribution across cells

(firing rates or correlations with whisker movements here will be very similarly distributed), separated its left tail and right tail based on a first statistical assessment (see Supp Fig. 6a), then performed a second statistical test on the left tail vs right tail, it passes this second statistical test simply because I classified the cells based on the first one. The authors should remove stats that echo prior tests (any stat bars that compare modulated vs non-modulated in this case). If they still want to show and quantify the firing rate modulations WITHIN the modulated or non-modulated groups, that helps to understand the depth of the active vs passive modulation within the cell classes (only 2 stat comparison bars, a significant one for the modulated side and a non-significant one for the non-modulated), but comparisons between the groups are contrived. I think they should also show here that run firing rates at field center are indistinguishable between modulated and non-modulated cells to be more in line with their rebuttal argument ("… specifically, modulated and non-modulated cells exhibit similar excitability during running (i.e., comparable firing rates), despite the different rates during immobility "). They show something about this in 5f but only for corr coeff, so it would be simpler to see just run firing rates at field centers for non-modulated vs modulated, which they suggest are similar but I do not see it anywhere in the figures (or supp figures, apologies if I missed it).

4) Many heatmap figures have no color bar (3c at the right, 3d left, 4b, 4c, etc). Please add color bars to all heatmaps and carefully review figures for other missing labels and details.

Reviewer #3

(Remarks to the Author)
The authors provided a highly detailed and comprehensive rebuttal to the comments I and the other reviewers provided: I was impressed by the scope and depth of the new analysis and experiments presented in the rebuttal. Specifically, the authors strengthened 1) analysis of LFP signals (specifically theta power and frequency) during arousal states, 2) the cognitive representation of location during active immobility, by implementing bayesian decoding, and 3) a sound-only control during the place field induction experiments. I think these changes have made the story much more comprehensive, and strengthened the claims that behavioral modulation of hippocampal activity sharpens spatial representations, even when the mouse is stationary. In light of these improvements, I have some minor comments. Should these be addressed I would be happy to see this work published:

Minor Comments:

1. Figure 2b,c
- What level were statistical tests over? n=32/34 sessions according to the legend of b, but the p-values/error bars seem quite small given that n. Are the tests calculated over all whisking events or average zscore of the change in LFP power per session?
- Line 90: “sharp-wave ripples predominantly occurred during ‘quiet’ immobility.” This statement suggests that ripples did not occur during ‘active’ immobility, but is unclear what the true ripple rate is during the active state because it is a z-score (ie. despite decreasing relative to the quiet state, the ripple rate could be quite high for all we know). It would be more transparent to show the raw ripple rates or to change the interpretation accordingly (“there is a decrease in the ripple rate after a transition to ‘active’ immobility”). If there truly are no ripples in the active immobile state, this is a very interesting finding and should be reported clearly.

2. Figure 2g
I appreciate the new decoding analysis, however, the presentation of decoding error as a z-score relative to shuffled error is somewhat confusing. I am curious what the true error of the decoder is (traditionally reported as average error in cm), which, as far as I can tell, is not plotted or reported anywhere. Maybe a more transparent way to present this would be to show the confusion matrix and shuffle distribution Supp Fig 2b in main figure 2, and to instead label the x axis of the shuffle distribution as the true decoder error in cm. Then indicate the z-score of the true error relative to the shuffle. I think this would make the exact procedure/change in error plotted in 2g much more clear.

3. Figure 3
The sound-only control is a nice addition, but the general role of this finding in relation to the other results presented here is still unclear to me. While I agree with the author’s statement: “This evidence … indicates that place field plasticity mechanisms can be engaged even in the absence of locomotion”, this finding would be much more noteworthy in comparison to a similar induction experiment during “quiet” immobility (ie. in a closed-loop fashion when whisking did not occur). To me this result only demonstrates that the place field induction protocol is effective: I am not surprised that it works whether the mouse is running (as in Bittner 2015) or awake and immobile (here). Given unlimited experimental time and a closed loop system, if the authors were to use the same induction protocol during running, “active” immobility, and “inactive” immobility and found it equally likely to induce a place field in all three conditions, it would simply demonstrate the induction protocol works regardless of behavioral state, and the contribution of this finding becomes somewhat moot given the story of the paper. Without these other conditions, it is impossible to say whether the induction protocol “just works” or if there is something special about active arousal states. I don’t think there is anything more to address here if the authors cannot do the same experiment during “quiet” immobility, but this result doesn’t say anything about how arousal affects place cell formation and therefore feels out of place.

4. Figure 4a
- Line 159: “theta oscillations during immobility showed a larger variability in frequency”. Based on the data presented this is not clear to me, the frequency distributions on the top of 4a seem quite similar to me… if anything there looks like a

frequency shift rather than change in variability.
- In several places in the text here, “theta/delta ratio” is referred to as “theta power” (line 160, 175). I am not sure if this is common convention, but seems somewhat misleading?

5. Figure 5g
- Line 234: “Spatial modulation was predominantly preserved in behaviorally-modulated place cells during active immobility.” I’m not sure what this sentence means, both modulated and non-modulated cells appear to have spatial “modulation” (ie. are spatially tuned) during active or quiet immobility. Did you mean to say something like “spatial representations were primarily modulated during ‘active’ immobility” or "modulation of spatial tuning was predominantly observed in behaviorally-modulated cells during 'active' immobility"?

6. Typos:
Line 77: “while during locomotion” isn’t grammatically correct
Line 223: “downsamling”
Line 313: grammar issue “heterogeneity at the … properties”, maybe you meant “level”?

Reviewer #4

(Remarks to the Author)
I co-reviewed this manuscript with one of the reviewers who provided the listed reports. This is part of the Nature Communications initiative to facilitate training in peer review and to provide appropriate recognition for Early Career Researchers who co-review manuscripts.

Version 2:

Reviewer comments:

Reviewer #2

(Remarks to the Author)
I thank the authors for the last revisions. I still have a lingering problem, but I think despite that, the paper is ready for publication because all I am asking them to do is remove two stats tests.

However, I will still try one more time to explain because I don’t think the authors have accepted or addressed one comment. Down-sampling is not fixing the problem, nor is firing rate adjustment.
It is a logical problem. So I will try one last time to make it clear why they should remove the stats test bars between the 1st and 3rd row and the 2nd and 4th row in Fig 5h. The comparison between rows 1 and 2 is valid (modulated cells FR ratio in active vs passive state), as is the comparison between rows 3 and 4 (non-modulated cells FR ratio in active vs passive). These quantify the firing rate modulation depth that is depicted also in Fig 5g.

Now, to see what I have a problem with, they should make a scatter plot, at first of all cells with Behavioral Modulation index (BMI) on the x-axis and the firing rate during "active" immobility divided by the firing rate during running (FRactiveimmob / FRrunning) on the y-axis (this is what they quantify on the x-axis in Figure 5h). Modulated cells (determined by their BMI stats test) will be skewed towards the upper right quadrant of this plot. They have higher modulation indexes (pushing them to the right on the x-axis), and higher FRactiveimmob / FRrunning ratios (pushing them up on the y-axis). If this is wrong, I apologize for dwelling on it too long, but I don’t see how it can be different.

They can now color the modulated cells orange.

The remaining non-modulated cells should not be skewed and be more centered on this plot. They can leave them blue. So, there should be two reasonably separable clusters of dots on the plot in orange and blue (maybe some small overlap because some cells are noisier than others), with orange dots skewed up and to the right. This separation should be apparent on both axes, x- and y-, which indicates these values are correlated. This correlation between BMI and FRratio is a problem because it is definitional, I don’t see how (based on their definitions and FR normalization to running) a cell that has relatively higher firing rates during whisking (normalized to running) would not also have a high whisking cross-correlation peak compared to shuffle. For me, the two measures are definitionally related to each other. This is why we cannot classify cells into two groups based on the x-axis stats and then perform a second stats test on the two group averages based on y-axis stats. A neuron that has higher cross-correlations with whisking, will also have higher Firing rates during whisking normalized to run. I cannot do better in explaining this, and I urge the authors to not perform redundant statistics. Removing these tests changing nothing regarding their conclusions, it simply doesn’t flood the reader with unnecessary tests.

The rest is great for me and I thank the authors for their patience and perseverance.

Reviewer #3

(Remarks to the Author)
The authors have addressed my concerns and I would be happy to see this work published in Nature Communications.

Open Access This Peer Review File is licensed under a Creative Commons Attribution 4.0 International License, which permits use, sharing, adaptation, distribution and reproduction in any medium or format, as long as you give appropriate credit to the original author(s) and the source, provide a link to the Creative Commons license, and indicate if changes were made.
In cases where reviewers are anonymous, credit should be given to 'Anonymous Referee' and the source.
The images or other third party material in this Peer Review File are included in the article's Creative Commons license, unless indicated otherwise in a credit line to the material. If material is not included in the article's Creative Commons license and your intended use is not permitted by statutory regulation or exceeds the permitted use, you will need to obtain permission directly from the copyright holder.
To view a copy of this license, visit https://creativecommons.org/licenses/by/4.0/

Dear Editor, dear Reviewers,

We thank the Reviewers for their helpful and constructive feedback. As you will see from the Point-By-Point Reply below and the corresponding changes made to the manuscript, we have done a major revision. Specifically, we have performed additional experiments, as well as analysis, which have resulted in an extensive revision of the **main Figures** and of **Supplementary Figures**. In this reply, we additionally present **13 Reviewer_Figures** and an **Addendum**, which answers issues raised by multiple reviewers.

In essence, we implemented the following changes:

1. *Bayesian decoding analysis.* To confirm the presence of spatial coding during immobility, we reconstructed the online location of the animal during this time. With this analysis we showed that the place representation is best preserved during active immobility, and that behavioral modulation is a unique feature of spatial coding during immobility (see section '**A3. Bayesian decoding analysis**' of **Addendum** at the end of this document).
2. *LFP quantification upon behavioral state transitions.* We quantified the spectral power of the LFP in different bands (theta, delta, gamma) upon behavioral state transitions, which confirmed our former qualitative observations (details in section '**A2. Improved visualization and quantification of theta oscillations**' of **Addendum** at the end of this document).
3. *Reanalysis of results related to theta oscillations.* We conducted a thorough reanalysis of the results related to theta oscillations. First, we implemented a theta segmentation based on time-resolved spectrograms, using standard criteria on theta/delta ratios. This set the basis for an improved quantification of theta oscillations during immobility. Second, we confirmed the findings related to the behavioral modulation of theta oscillations with linear regressions. This analysis confirmed all points shown in the former manuscript and strengthened its conclusions (see section '**A1. Revised theta oscillations analysis**' of **Addendum** at the end of this document).
4. *Additional single-cell stimulation experiments,* including sound-only controls, in which cells were not electrically stimulated. With this control, we confirmed that the juxtacellular stimulation itself, and not the sound, was responsible for the emergence of *de novo* place fields at the stimulation location. Furthermore, we have also reanalyzed and improved the display of our data, which are now shown as main Figure 3 (details in section '**A4. Sound stimulation controls**' of **Addendum** at the end of this document).
5. *Improved the clarity of the text.* Following the Reviewers' suggestions, we have improved the clarity, precision and flow of the text. We have also added a 'Dataset overview' section in the Methods, which clarifies inclusion criteria for each analysis (see also section '**A5. Dataset overview**' of **Addendum** at the end of this document).
6. Following the Reviewers' suggestions, we have performed a more solid statistical analysis of our data, including a per-session reanalysis of the main results. Details are provided in response to the individual comments.
7. We have revised the manuscript text to fully address the Reviewers comments. For ease of reading, new and significantly modified text in the revised manuscript is indicated in red.

Altogether, the above changes resulted in a more rigorous and greatly improved manuscript, which we hope will now be acceptable for publication in *Nature Communications*.

Andrea Burgalossi
(on behalf of all authors)

NOTE: For clarity, similar reviewer comments have been addressed collectively in a dedicated **Addendum** at the end of this document, which we refer to when responding to the relevant comments below.

Point-by-point Reply (Reviewers' comments in bold)

Reviewer #1:

This study provides insights into the modulation of activity in place cells and accompanying oscillatory dynamics in the dorsal ca1 region of the hippocampus. The authors simultaneously captured facial expressions and online brain activity, while mice were engaged in space clamped vs. non-clamped experiments on a belt. By aligning traces of facial movement, local field potential of the region and single unit analyses, authors provide evidence that place cell like activity and theta oscillations emerge in some cases in which the mouse is not in locomotion. Specifically - this activity is related to the arousal level of the animal, as measured by pupil dilation and engagement of the mouse in whisking behavior. The authors propose a layer of encoding mechanism that incorporates arousal level in a multiplexed model of episodic memory processing. This behavioral-state related modulation in the brain regions can potentially support curation of episodic memory content and has implications in understanding the nature of hippocampal outputs during encoding and consolidation of such memories.
While the study is of interest, we feel that additional work has to be done in order to incorporate the results into the known body of literature related to place cells and their function, and the relation between the place cells and the local field potential. Also, more effort should be done to explain what is new here relative to previous findings.
COMMENT: We are thankful for the positive assessment of our work, and for the constructive feedback. As the reviewer will see from the detailed responses below, we have performed additional experiments and analysis for rigorously addressing the points raised by the reviewer. This major revision effort has allowed us to strengthen the conclusions of our work, making the study more robust and coherent. We believe that, taken together, our results provide several key and novel contributions, which we summarize below for clarity:

1. **Place coding**. CA1 place cells, classically studied during locomotion, also encode spatial information during immobility.
2. **Gating**. The CA1 spatial code is gated by arousal, such that a reliable place code emerges in a high-arousal state (see also new decoding analysis).
3. **Heterogeneous behavioral modulation of place cells**. During immobility, CA1 place cells differ in their degree of behavioral modulation, excitability, and in their contribution to spatial representations. These differences (which are not observed during run) imply that cell recruitment (allocation rules) in a memory engram may differ between immobility and locomotion.
4. **Behavioral modulation of theta oscillations**. During immobility, theta oscillations are modulated by arousal levels, rather than reflecting a simple dichotomy (i.e. Theta "Type-1" versus "Type-2").
5. **Place cell plasticity**. Place field induction, previously demonstrated only during locomotion, can also occur in the absence of locomotion.

__**MAJOR ISSUES**__
1. The authors separate in the paper two populations of cells, one which is behaviorally modulated why whisking and arousal, and a second that is not. This a very strong claim. In order to make it, the

authors should present some sort of a bi-modality. We suspect that their cells are part of a continuum, and thus it is hard to state that these are two populations.

COMMENT: We thank the Reviewer for this comment and apologize for not having sufficiently clarified this point in the previous version of the manuscript. Indeed, behavioral modulation of place cells is best captured by a continuum (acknowledged now in the Results, page 8 line 207 and in the Discussion, page 10 line 280), since we did not observe evidence of bimodality in the distribution of modulation indices (see e.g. **Supplementary Fig. 6a**), nor any anatomical distinction between cells along this dimension (see **Supplementary Fig. 6f,g**). In the previous version of the manuscript, we statistically partitioned this continuum into two subgroups – one showing significant positive modulation and one not – and referred to these as two distinct "populations". In the revised version, we now clarify this interpretation explicitly and have modified the display in revised **Fig. 5c** to better convey that behavioral modulation should be viewed as continuous rather than categorical.

We note that, also in line with comment 17 from Reviewer #2, we have further refined our cell classification criteria and tested for significantly negatively modulated cells in our dataset. We found that only a minority of place cells (n=4) passed the significance test (see details in response to comment 17 from Reviewer #2).

CHANGE: To address the Reviewer's comment, we have changed the organization of **Fig. 5 c-e** to better reflect a continuum. In the revised results, (page 8 line 207) and discussion (page 10 line 280) we also refer to this point. Specifically, we state "*the Behavioral Modulation Index (…) did not show a clear bimodal distribution, but rather appeared to reflect a continuum*" (results, page 8 line 207) and "(..) *we found that – along a behavioral modulation continuum* (**Fig. 5c-e**) (…) (discussion, page 10 line 280)". We have added additional panels to **Supplementary Fig. 6f,g** to validate the anatomical location of units across along the probe shank (as in [1,2]).

Furthermore, in the example in Figure 4b, it seems that the increase in firing of the non-modulated cell occurs at the offset of the whisking. Could this be the case in general for the non-modulated cells?

COMMENT: We agree with the Reviewer that this representative instance might give the impression of whisking-offset associated firing. However, we systematically examined this possibility across all recorded cells and found no evidence of increased firing at the offset of whisker-pad motion in non-modulated (as well as modulated) cells (**Reviewer_Figure 1**).

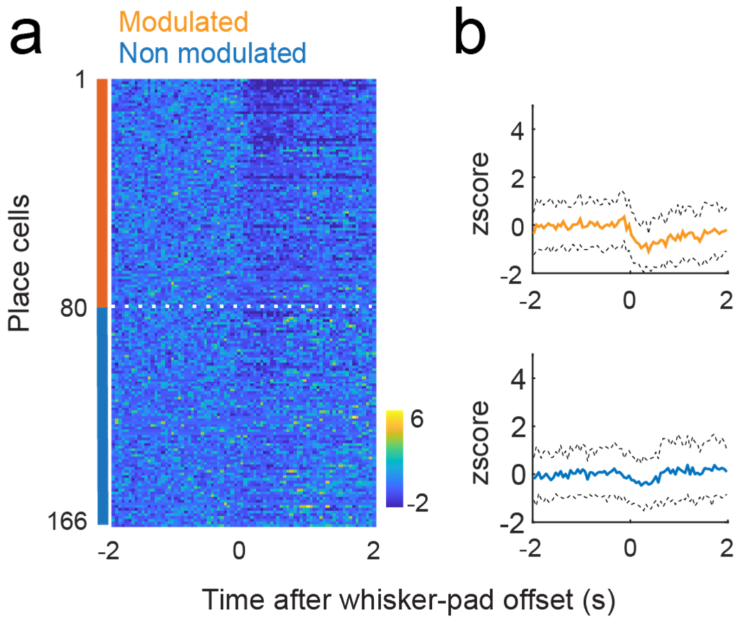

Reviewer_Figure 1. PSTHs upon transitions from active to quiet immobility.
(a) Peristimulus-time histograms (PSTH) for for modulated (orange) and non-modulated (blue) cells (n=166 place cells) upon transitions from active to quiet immobility (z-scored with respect to baseline i.e., active immobility).
(b) PSTHs as in (a) for modulated (orange) and non-modulated (blue) cells. Error bars indicate SEM.

CHANGE: none.

2. The second issue related to this is that cells in CA1 can remap, and thus the results should be interpreted in relation to remapping. It is more probable that the non-modulated group are actually cells belonging to a different map, and not an anatomically-distinct group, as the authors suggest in the discussion.

COMMENT: We appreciate the Reviewer's concern. However, we would like to note that in our data, Pearson's correlations between place maps computed before and after immobility were, on average, high (0.72 ± 0.22; **Supplementary Fig. 1c**), indicating that no systematic remapping occurred during the experiment. Furthermore, by design, we included in the analysis only recording segments with high pre–post ratemap correlations (> 0.7; see section 'Place cell analysis' in Methods). Importantly, pre–post correlations did not differ between modulated and non-modulated cells (p = 0.58, Wilcoxon rank sum test; see **Reviewer_Fig. 2c**), indicating that both groups exhibited comparable spatial stability. Together, this evidence rules out the possibility that differences in behavioral modulation arose from systematic remapping effects. We now refer to this point in the revised manuscript.

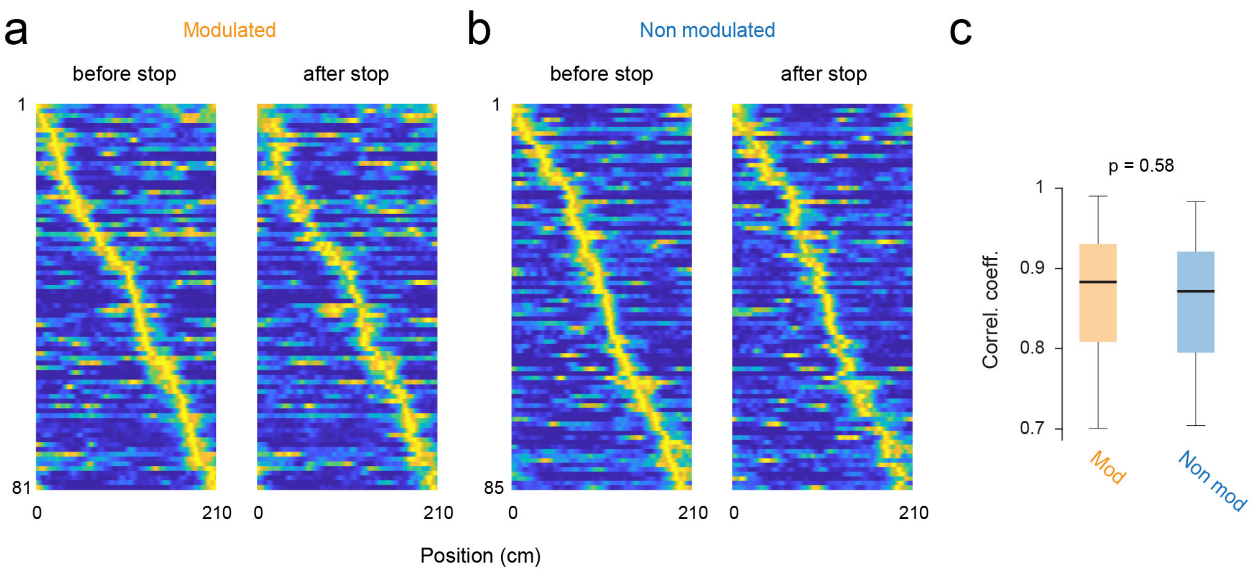

Reviewer_Figure 2. Stability of spatial code in modulated and non-modulated cells.
(a,b) Normalized ratemaps before and after immobility for modulated (orange, n=81) and non-modulated (blue, n=85) cells.
(c) Pearson's correlations for the ratemaps before and after immobility for modulated (orange, n=81) and non-modulated (blue, n=85) cells. p=0.58, Wilcoxon signed-rank test.

CHANGE: In the revised supplementary information (page 3 lines 133-135) we now state that "*behaviorally-modulated and non-modulated cells (**Fig. 5**) did not differ in spatial stability (pre-post immobility ratemap correlations, p = 0.58, Wilcoxon rank sum test)*".

3. The fact that place cells continue to fire at high levels during immobility if the mouse is within the place field of the cells suggests that the cells are still mapping space. The authors should attempt to run a decoder analysis with respect to the spatial map during immobile states.

COMMENT: Many thanks for this great suggestion (point also related to comments 4, 19 of Reviewer #2 and 1 of Reviewer #3). In the revised manuscript, we included a Bayesian decoding analysis during immobility, comprehensively described in the **Addendum** (see section '**<u>A3. Bayesian decoding analysis</u>**' at the end of this document).

We thank the Reviewer for this excellent suggestion, which allowed us to strengthen an important aspect of the work, namely: (i) that place cells retain spatial coding during immobility, (ii) that the spatial code is better preserved during active immobility, and (iii) behaviorally modulated cells selectively contribute to spatial coding during immobility.
CHANGE: The decoding analysis is now presented in the new **Fig. 2g, Fig. 5j** and **Supplementary Fig. 2**, and referred to in the revised Results (page 5 lines 119-129, page 9 line 243-253).

4. As the authors are dealing with a situation in which the mouse is undergoing different behavioral states, they should attempt to cluster those states and look at the behavior of the network, similar to Rubin, Sheintuch et al., 2019.
COMMENT: We agree with the Reviewer that while clustering behavioral states as in Rubin, Sheintuch et al., 2019 is indeed a powerful approach, we feel it goes beyond the scope of the present study. In the present manuscript, our goal was to partition behavioral states in a simple binary manner based on clear changes in a single variable (whisker-pad motion), which we found was sufficient (within the scope of the study) to reveal meaningful differences and to characterize hippocampal dynamics across distinct arousal states. We feel a more detailed clustering of subtle awake-state variations would require a dedicated study on its own, since virtually nothing is known about how these fine-scale dynamics impact hippocampal representations and processing.
CHANGE: none.

5. Phase precession is reported in place cells with respect to ongoing Theta. Authors segmented the experiment in running, inactive immobility and active immobility epochs. Presence of theta band in the LFP during running and active immobility calls for phase precession related analyses might aid in differentiating network status during these behaviorally different epochs.
COMMENT: We thank the Reviewer for the great suggestion. Theta phase precession analyses in the spatial domain require uniform spatial coverage, which holds true for our running condition. Unfortunately, this was not the case for the immobility condition because, as also noted in response to Reviewer #2 (comment 11) and Reviewer #3 (comment 1), we do not have systematic sampling across all belt locations during immobility. Instead, each session contained only a limited number of immobility periods (ranging from 1 to 9; details now provided in the revised Methods), resulting in sparse spatial coverage. For this reason, we did not attempt to perform a "standard" theta phase-precession analysis during awake immobility in the previous version of the manuscript.
However, we agree that this is a conceptually important aspect, as it can provide insight into the temporal (and not only rate-based) components of the hippocampal code. Therefore, despite the above limitations, we have now explored two complementary analyses – one in the temporal domain and one in the spatial domain – to assess whether place cells exhibit fine spike-phase organization during immobility periods.
First, as a necessary pre-validation, we performed phase precession analysis during running. Indeed, in our experimental preparation, we do observe reliable phase precession (see **Reviewer_Fig. 3a**).
Next, we explored if we could observe time-precession[3] during periods of immobility. For this, we extracted epochs of theta oscillations occurring during whisker-pad motion, and checked if aligning spikes to the peak of theta power (or at the onset of theta oscillations) would reveal any spike-theta-phase organization. However, this analysis did not reveal an obvious systematic relationship (see **Reviewer_Figure 3b**).

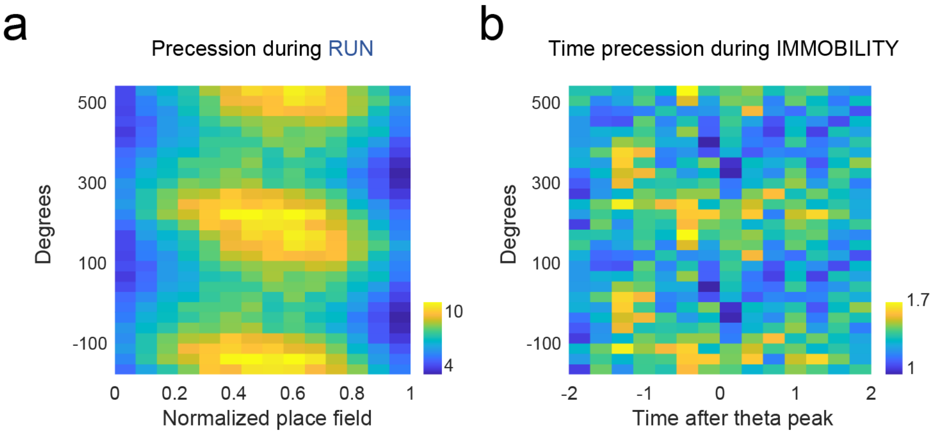

Reviewer_Figure 3. Absence of time precession during immobility.
(a) Heatmap showing the distribution of spikes of place cell units as a function of location within the place field and phase (in degrees) of the underlying theta oscillations (y axis). Data from "run".
(b) Heatmap showing the accumulation of spikes of place cell units during immobility, aligned to the peak time of theta epochs during immobility (x axis) and in function of phase (in degrees) of the underlying theta oscillations (y axis).

Second, during locomotion, the theta phase of spikes varies systematically as animals traverse a place field: spikes tend to occur near the peak of theta upon entering the field and shift progressively toward the trough as the animal exits. To test whether a similar spike–phase organization occurs during immobility, we selected all immobility events that took place within any identified place field. Using the fitted relationship between phase and position, we computed the expected theta phase corresponding to each immobility position along the place field. These expected values were then used to align and sort all phase distributions across cells recorded at stop locations during both running and immobility periods. While a clear population-level trend was evident during running, no such organization was observed during immobility (see **Reviewer_Fig. 4**).

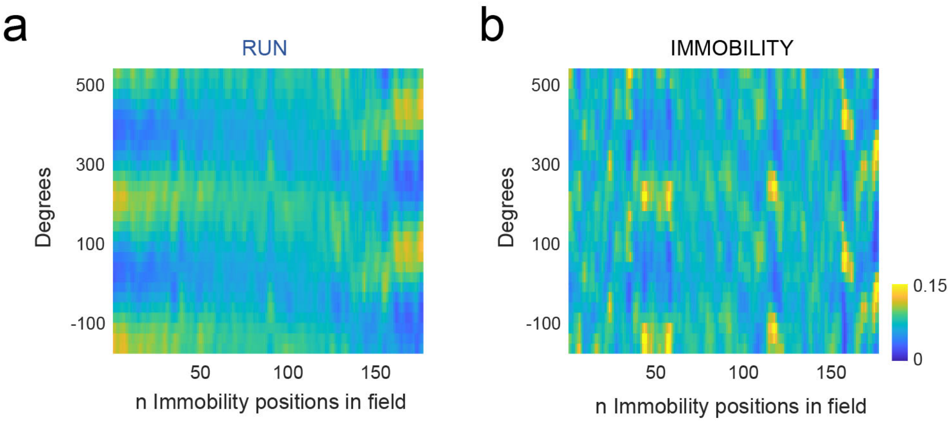

Reviewer_Figure 4. Absence of theta phase precession during immobility.
(a) Heatmap showing the population spike-phase distribution during running. Each column represents a place cell (spikes within ±10 cm of the stop location). The x-axis is sorted by the expected phase-precession slope at that location. Color code shows probability, same scale as in (b).
(b) Same analysis shown in (a) but from immobility periods, same sorting as in (a). Color code shows probability.

Based on the above observations, we conclude that the fine spike–phase organization characteristic of theta phase precession appears to be lost during immobility in our task (see **Reviewer_Fig. 3b**). However, we acknowledge that it is difficult to draw definitive conclusions from these data due to several limitations – namely, the variability of phase precession across cells, the sparse sampling of immobility locations along the belt, and the lower firing rates during immobility. Together, these factors limit our ability to make a stronger statement regarding the presence or absence of spike–phase organization in this behavioral state. Given the strong conceptual importance of phase precession for hippocampal computations, we decided not to include this preliminary analysis (rather inconclusive at this stage) in the revised manuscript. We hope the Reviewers agree with our assessment.
CHANGE: none.

6. The results in the paper may be related to the time cells literature (e.g. McDonald…Eichenbaum, 2011), and thus analysis for checking if there is phase coding relative to the onset and offset of the whisking behavior should be performed.
COMMENT: We thank the Reviewer for the comment. We note that time cells typically encode temporal information over short intervals, ranging from a few to tens of seconds, particularly when there is an 'explicit' demand for memory retention[4]. In our paradigm, however, immobility periods lasted approximately 5 minutes – considerably longer than the delays used in previous studies [4–6] and exceeding the capacity for short-term (hippocampal) memory retention. In line with this interpretation, analysis of place cell firing relative to the onset of whisker-pad motion did not show any temporally precise pattern across trials, nor across cells (see **Reviewer_Fig. 5**), which generally showed a sharp increase in firing upon behavioral state transitions (as in **Fig. 2d**). These observations indicate that temporal sequence coding is unlikely to have played a significant role in our experimental setting.

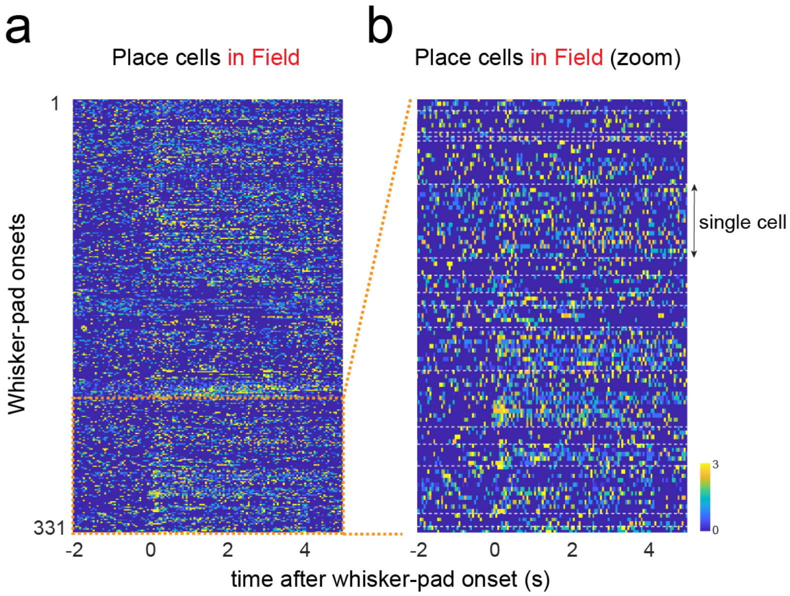

Reviewer_Figure 5. Absence of time-phase coding in place cells.
(a) PSTH showing the in-field firing of place cells (both behaviorally-modulated and non-modulated) upon transitions to active immobility, on a trial-by-trial basis (n=331 events, considering whisker-pad motion events of similar long durations i.e. > 5s, thereby providing an extended temporal window for analysis).
(b) Zoom of a portion of the PSTH in (a). Different horizontal white dotted lines separate different cells.

CHANGE: none.

7. We are missing a systematic quantification and statistics of the abundance of theta and other oscillations in different behavioral states, and as a function of arousal level. What was the level of coherence between theta LFP and whisking cycles?

COMMENT: We thank the Reviewer for the suggestion. In the new version of the manuscript, we quantified changes in relevant frequency bands during transitions from quiet to active immobility (theta, delta and gamma bands) which are now included in **revised Fig. 2.** For more information regarding improvements in **Fig. 2b**, please refer to the **Addendum** (see section '**A2. Improved visualization and quantification of theta oscillations**'). We have also added a quantification of changes in these bands as a function of arousal level (i.e., whisker-pad motion durations) which is now included in the new **Supplementary Fig. 4f**.

Regarding the coherence between LFP and the whisking cycle, our camera setup - imaging the animal's face from the side - does not allow for precise identification of individual whiskers or accurate extraction of whisking cycles, which is essential for such analyses. Accurate estimation of whisking cycles typically requires a high-speed top-down camera view[7,8]. We now specifically refer to this point in the revised methods.

CHANGE: This new analysis is included in **Fig. 2b,c** and **Supplementary Fig. 4d-f,** and referred to in the revised Results section (page 4 lines 88-91).

8. Many of the points in the discussion are very speculative. Some examples of unfounded claims:

1. ***"indicating that the gain-modulation observed at the population level (Fig. 2b-d) is primarily driven by a subset of behaviorally-modulated neurons"***

   COMMENT: We thank the reviewer for the comment. We acknowledge that in the previous version of the manuscript this claim was only indirectly supported by firing rate increases. In the revised version, we performed additional analysis, and we observed a multiplicative gain only in behaviorally modulated cells (see **Fig. 5i**; linear model slope $\beta \neq 1$, $\beta = 1.56 \pm 8.12 \times 10^{-2}$ SE, $t(79) = 6.87$, $p = 1.34 \times 10^{-9}$) and not observed in non-modulated cells ($\beta = 1.05 \pm 3.29 \times 10^{-2}$ SE, $t(83) = 1.41$, $p = 0.16$), further confirming our previous hypothesis (see also comment 2 of Reviewer #3). This analysis is shown in revised **Fig. 5i** and referred to in the revised Results (page 9 lines 239-242).

2. ***"The subset of behaviorally-modulated place cells might inherit modulation via direct (or indirect) inputs from the brainstem arousal centers, from the CA2 or from the upstream head-direction system"***

   COMMENT: We thank the reviewer for the comment. Following the reviewer's comment, we revised this part of the discussion (page 11, lines 301-312) and included additional text and citations to support our hypotheses.

3. ***"behaviorally-modulated and non-modulated place cells might correspond to different subtypes of pyramidal neurons"***

   CHANGE: Whether and how CA1 in-vivo activity maps onto structural and morphological heterogeneity has remained largely unresolved. In the revised manuscript, we have (i) elaborated on this point more explicitly (see Discussion, page 11 lines 313-319) and (ii) refined our analysis of anatomical location (see revised **Supplementary Fig. 6f,g**). Although the latter analysis suggests no obvious segregation along the radial deep–superficial axis (within the resolution limit of extracellular recordings), it remains to be determined whether finer morphological organization exists – for example, at the level of dendritic architecture[9,10], axonal initial segments[11], or molecular expression patterns[12].

We believe it is therefore useful to include such a statement in the discussion to encourage future studies to address this question.

"This evidence suggests that behavioral state fluctuations set different theta oscillatory regimes in the hippocampus, and these effects are likely mediated by local FS interneuronal networks"
COMMENT: we have reworded the corresponding statement (see Discussion, page 12 lines 346-349)

4. ***"we speculate that rearing might correspond to a higher-arousal state than locomotion".***
COMMENT: We have removed this sentence from the revised manuscript.

MINOR ISSUES

9. Authors mention the ethological significance of intermittent pauses during naturalistic behavior in their discussions, but random pauses in their experiment, seem to be experimentally controlled (Sup Fig1b) to achieve pauses across multiple place fields. Can they add a comment, if an attempt was made to distinguish active pauses with failed attempts to move?
COMMENT: The reviewer is correct that the "immobility periods" in our experiment are experimentally imposed (we now refer more specifically to the experimental design in the revised Results, section 'Hippocampal place cell recordings and behavioral analysis'). Unfortunately, our setup did not allow measurement of failed attempts to move; however, we expect their contribution to be minimal, since animals were gradually habituated to remain at fixed locations on the treadmill over several days, typically becoming accustomed to imposed immobility within ca. one week.
CHANGE: In the revised manuscript, we have explicitly stated in the Results (page 4, lines 61-62) and Methods (page 1, line 21) that immobility was experimentally controlled. Additionally, we updated the schematic in **Fig. 1a** to include a depiction of a "brake".

10. Can authors add a line, about the rationale of switching illumination status of the experimental setup between active and immobile periods?
COMMENT: A single LED (positioned at the end of the treadmill and oriented towards the wall) was used to signal when the animal was allowed to run (i.e. brake released). This single LED did not change the luminosity of the setup, but was used as a cue to facilitate training. We have clarified this in the revised Methods.
CHANGE: we refer to the point above in the revised methods (page 1 lines 30-31).

11.1. Can authors add a line, explaining the rationale of the auditory pulse, preceding juxtacellular stimulus to induce a place field?
COMMENT: Auditory pulses, such as click sounds, are commonly used to evoke states of high arousal[13–15]. In particular, recent work from our lab has shown that click sounds presented to awake, head-fixed mice reliably induce transitions to active immobility, characterized by stimulus-locked whisker-pad motion and pupil dilations[15]. In our experimental design, the click sound was used to consistently evoke an active immobility state prior to single-cell stimulation. Our rationale was to specifically deliver the single-cell stimulation during active immobility, as this state is associated with higher in-field excitability (**Fig. 2e,f**) and better-preserved spatial coding during immobility (**Fig. 2g**). Therefore, active immobility was the primary candidate condition for testing place field plasticity mechanisms, with the auditory stimulus serving as a tool to ensure that juxtacellular stimulation occurred during an active behavioral state.
CHANGE: We have clarified the rationale in the revised Results (page 6 lines 142-144).

11.2. What was the reason to believe that place cell was due to injected current but not due to behavior or sound stimulus?
COMMENT: We thank the reviewer for this suggestion, which is also related to comment 2, 12 of Reviewer #2 and comment 5 of Reviewer #3. To address the reviewer's concern, we conducted additional sound stimulation control experiment, which are comprehensively descripted in the **Addendum** (see section '**A4. Sound stimulation controls'** at the end of this document).
CHANGE: The results of the new experiments analysis outlined in the **Addendum** (section section '**A4. Sound stimulation controls'**) is now shown in the new **Fig. 3** and referred to in the Results (page 6 lines 138-150).

12. The division into short, medium and long events is a slightly arbitrary analysis – better to do regression.
COMMENT: We thank the reviewer for this great suggestion, also related to comments 2 and 14 of Reviewer #2. To address the Reviewer's concern, we reanalyzed theta oscillation related results using regressions and quantile groups. For details, please refer to the **Addendum** (see section '**A1. Revised theta oscillations analysis**' at the end of this document).
CHANGE: Regression analysis and quantile groups are shown in the revised **Fig. 4e,f** for theta oscillations and in **Fig. 4g,h** for interneuronal firing.

13. Figure 4d, assumes only immobility, please separate into active vs. inactive immobility.
COMMENT: We thank the Reviewer for the suggestion. We show the data separated into active vs inactive immobility, as suggested, instead of the "immobility" only.
CHANGE: We present the separation suggested by the Reviewer in **Fig. 5h**.

14. Language issue: footnote for sup 3c has an English problem.
CHANGE: We have fixed this mistake.

15. Please add units for X axis in Figure 4e (Hz ?).
CHANGE: We have fixed this mistake.

16. What was the relation between the cells activity and the reward? Could it have affected the results?
COMMENT: We thank the reviewer for the question. First, we note that epochs associated with reward consumption (and immobility at the reward location) were excluded, as only spikes occurring during locomotion (>1 cm/s) were included in the analysis (see Methods for details). Second, behaviorally modulated and non-modulated place cells did not differ in the distribution of place fields around the reward (behaviorally modulated: 16/81 cells; non-modulated: 20/85 cells; p=0.58, Fisher's exact test; see **Reviewer_Fig. 6**). This suggests that reward cells (as e.g. in [16]), if present in our data, were equally represented in both groups and are therefore unlikely to have biased our results. We also note that reward-modulated cells, as reported by [16], were preferentially located near the distal CA1/subiculum border, whereas our recordings targeted more intermediate CA1 regions (see representative histological image in **Reviewer_Fig. 9**).

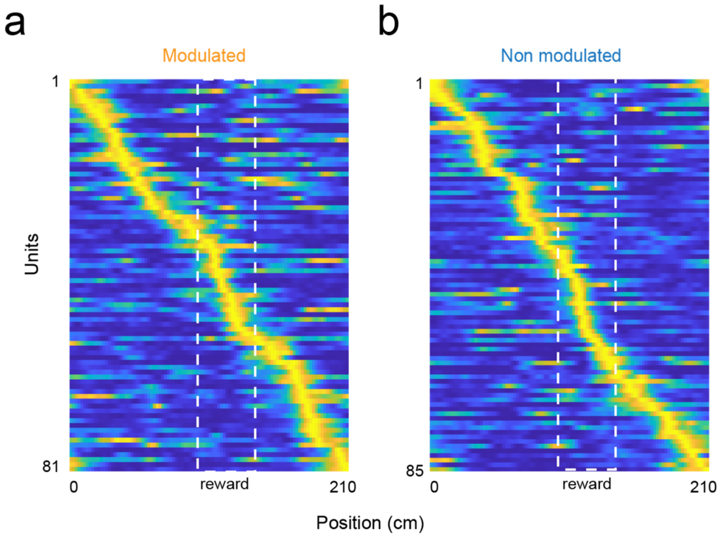

Reviewer_Figure 6. Modulated and non-modulated cells do not differ in the place field distribution around the reward zone.
(a) Normalized ratemaps for modulated cells (orange, n=81 cells). Reward zone location is indicated with white dashed lines.
(b) As in (a) but for non-modulated cells (blue, n=85 cells).

CHANGE: we refer to the above analysis in the revised Methods (page 3 lines 125-128) where we state "*No difference in the distribution of place fields around the reward zone (between 126 and 158 cm, centered on the reward location) was observed for modulated and non-modulated cells (modulated: 16/81 cells; non-modulated: 20/85 cells; p=0.58, Fisher's exact test).*".

Reviewer #2:

This study from Sartorato et al. shows that place fields in dorsal CA1 are somewhat intact (reduced peak firing rates) during periods of imposed immobility. The immobility place fields are more pronounced in about 50% of the CA1 place cells during Active immobility states, as defined by whisker pad movement and pupil dilation. Longer durations of Active immobility accelerate theta rhythms, increase relative theta power, and enhance entrainment of FS putative interneurons to theta. While these findings are both interesting and novel, the analysis is rough, at times incomplete, and at other times, regrettably, incorrect. There are major revisions necessary to publish this manuscript.

COMMENT: We are glad the Reviewer believes the results are interesting and novel. We thank the Reviewer for the constructive feedback. We have done our best to address all points raised by the Reviewer. Specifically, to address the comment about the analysis, we have undertaken a major revision effort, which is briefly summarized here:

Spatial coding: We have extended our analysis of spatial coding by including a decoding analysis (see more details in **Addendum, '<u>A3. Bayesian decoding analysis</u>'** at the end of this document; related to comment 3 of Reviewer #1, comments 4.1 and 19 of Reviewer #2 and comment 1 of Reviewer #3). The results are shown in **Fig. 2g, Fig. 5j** and **Supplementary Fig. 2c.**

LFP analysis: We have included a quantification within different LFP bands (e.g., theta, gamma, delta, ripple), and refined and extended our theta and ripple analyses (see more details **Addendum**, '**<u>A2. Improved visualization and quantification of theta oscillations</u>**' at the end of this document; related to comment 10 of Reviewer #2 and comment 4 of Reviewer #3). The results are shown in **Figure 2b,c.**

Gain modulation: We further investigated the nature of gain modulation between modulated and non-modulated cells (see more details in response to comment 8.1 of Reviewer #1 and comment 2 of Reviewer #3). The results are shown in **Fig. 5i.**

Theta analysis: This analysis has been improved in multiple ways, providing a more comprehensive assessment of theta dynamics (see section **Addendum**, '**<u>A1. Revised theta oscillations analysis</u>**' at the end of this document; related to comment 13 of Reviewer #2 and comment 4, 12 of Reviewer #3). We support these findings with additional analysis on FS interneuron dynamics (see comment 16 of Reviewer #2). The results are shown in **Fig. 4**.

Anatomical localization: We refined our anatomical localization of single units, following the procedures of [1,2] (see more details in response to comment 1 of Reviewer #1). The results are shown in **Supplementary Fig. 6f,g.**

Reactivation analysis: We have looked at neuronal reactivations during ripples with spatial decoding analysis, and observed preferential reactivation of reward locations (see more details in response to comment 9,18 of Reviewer #2 and comment 3 of Reviewer #3). The results are shown in **Supplementary Fig. 3.**

Single-cell stimulation: We performed additional control experiments and analysis (see **Addendum** section '**<u>A4. Sound stimulation controls</u>**' at the end of this document; related to comment 11.2 of Reviewer #1, comment 12 of Reviewer #2 and comment 5 of Reviewer #3). The results are shown in **Fig. 3.**

Per-session analysis: We have reanalysed our data on a per-recording session basis (see more details in response to comment 2 of Reviewer #2). The results are shown in **Supplementary Fig. 2.**

We believe that altogether, these revisions – including additional analyses and experiments – have substantially strengthened the manuscript, providing a more robust and solid presentation of the data.

Details are provided in response to the individual comments here below.

<u>**My major concerns are:**</u>
1. "Whisking" in mice is a sampling behavior where the angular position of the entire whisker array oscillates at 10–20 Hz, creating rhythmic protraction/retraction around a slowly varying set point. The author's claim that mice are "whisking" (line 78 and many other places in the manuscript) is not valid because it is simply based on a facial motion energy measure. Whisker pad motion during running could follow other, slower, rhythms (like breathing) or it could be uncoordinated and unique to locomotion. To show that the mice are "whisking" while they run, the authors need to perform a more standard analysis looking at the frequencies and correlations of whisker-pad-wide movements. I am not sure that this is possible in their recording configuration (video of face and not top view of whiskers). That being said, I think they could also just not use the word "whisking", but rather a behavioral state comprised of immobility and high whisker pad motion. The whisker motion during running is not crucial because it is not variable and they do not use it to define any behavior states.
COMMENT: The Reviewer is correct. We refer to whisker-pad motion as "whisking," based on evidence that mice display prominent whisking during locomotion[17,18] and on previous reports describing spontaneous whisking and associated facial movements in similar awake, head-fixed preparations[19] (there are also studies that refer to whisker-pad-motion as "whisking", [20]). While we are confident that whisker-pad motion in our study reflects whisking behavior, we acknowledge that we cannot rigorously and quantitatively support this claim, since our camera setup (side-view imaging of the face) were not optimal for tracking individual whisker movements and retraction-protraction whisking cycles (see also response to Reviewer #1, comment 7).
CHANGE: Following the Reviewer's suggestion, we have revised the text to avoid directly referencing "whisking" and now refer to this measure consistently as "whisker-pad motion".

2. The statistics are not correctly done and some controls are lacking. It is never clear how many co-recorded neurons are being presented (how many cells per mouse per recording etc.). Co-recorded cells are not independent and thus should not be tested as independent samples (N should not be number of neurons, but rather number of recordings except for the juxtacellular case where 1 neuron was recorded at a time). In Figure 1 there are no stats, in Figure 2 stats are on Ncells, in Figure 3 stats are done on arbitrary "duration" groups with different window sizes and counts (see below for more precise comments), and in Figure 4, the modulation index is incomplete, the firing rate analysis is statistically circular (see below) and the claims about ripples are not statistically grounded. It is therefore difficult to judge if the results of the paper will stand up to more rigorous statistical assessment. I find it strange that 2 specific claims in the abstract (in brackets below, see further below for detailed explanations) are only featured in supplemental figures, and are based on very shaky statistical or experimental evidence: *"The hippocampal spatial code was preferentially contributed by a subset of behaviorally-modulated place-cells, which increased their firing upon transitions to active immobility and [were preferentially recruited during awake ripple oscillations]. [Single cell stimulation was sufficient for the induction of place fields], indicating that plasticity mechanisms can be engaged even in the absence of locomotion."*
COMMENT: We thank the Reviewer for highlighting important points regarding our data presentation and statistical analyses. For clarity, we address these issues in separate sections below, beginning with the more general points.
The Reviewer raised a concern related to the computation of statistics using cells as individual units. While we acknowledge that this is, in principle, a valid concern, it is standard practice in hippocampal research to treat place cells as independent units (see, e.g., [3,21–24]). This assumption is supported by extensive evidence

showing that individual cells map space independently of one another (see e.g. remapping, and the uncoordinated behavior of cell-pairs relative to one another; for a review, see [25,26]). Moreover, the standard Bayesian decoders widely used in the hippocampal literature also rely on the assumption of independence of place cell firing given the animal's position[27–29]. Thus, within this framework, we believe that the presentation of our results in this format is consistent with standard practice in the field.
In spite of the above consideration, in response to the Reviewer's concern that "*N should not be number of neurons, but rather number of recordings*," we recomputed statistics on a per-recording-session basis. These results are now presented in the new **Supplementary Fig. 2c,e.** Importantly, the conclusions remain consistent with our previous analysis, further strengthening and rigorously supporting our observations.
CHANGE: in the revised manuscript, we now present session-level analysis for the main results of our study in **Fig. 1d, Fig. 2b, Fig. 2c, Fig. 2d, Supplementary Fig. 2e, Supplementary Fig. 2c, Supplementary Fig. 3c-e, Supplementary Fig. 4.**

The Reviewer also raised potential issues with specific analyses, which we address individually below or in response to following related comments:

(i) … It is never clear how many co-recorded neurons are being presented (how many cells per mouse per recording etc.) …
CHANGE: We have included this information in an additional panel in **Supplementary Fig. 6c**, where the number of recordings per-mouse is shown.

(ii) … In Figure 1 there are no stats …
CHANGE: In the **revised Fig. 1**, statistics have been added to the figure legend.

(iii) … in Figure 3 stats are done on arbitrary "duration" groups with different window sizes and counts (see below for more precise comments) …
CHANGE: This aspect is addressed in response to comment 10.

(iv) … Figure 4, the modulation index is incomplete …
CHANGE: This aspect is addressed in response to comment 17.

(v) … the firing rate analysis is statistically circular …
CHANGE: This aspect is addressed in response to comment 18

(vi) … the claims about ripples are not statistically grounded …
CHANGE: This aspect is addressed in response to comments 9 and 18.

3. The presentation of the data is incomplete and sometimes obscure. In Figure 2, the authors segregate immobility periods into active and quiet. In figure 3, they only compare active immobility and running, leaving the reader wondering what happens during quiet immobility for the same measures. Then in Figure 4, they start looking at general immobility (both active and passive pooled, panels d and e), only to statistically quantify active vs quiet immobility (panel g). There are three behavioral states defined starting in Figure 2: Run, Active immobile, Quiet immobility… and I do not see why all behavioral states are not quantified together for all figures. It seems suspicious that they are not all treated in the same way for statistics. Even when normalizing to the peak of Run place fields, we should see the Run curve on the plot because the sharpness of the place fields might be different and interesting in and of itself.

COMMENT: We thank the Reviewer for this comment. We agree that our presentation should have been improved. To address this comment, we have made our best efforts to (i) clarify the rationale for the data presentation, (ii) to better interconnect the findings and create a more coherent and logical storyline, and (iii) present the data and statistical analyses as transparently as possible.
Regarding the data presentation. The reviewer correctly states that "*There are three behavioral states defined starting in Figure 2: Run, Active immobile, Quiet immobility… and I do not see why all behavioral states are not quantified together for all figures. It seems suspicious that they are not all treated in the same way for statistics"***.** We apologize for not having clarified this issue. In fact, the three conditions cannot be treated the same systematically across all analysis, because a limitation of our experimental design is that we do not have systematic sampling across all belt locations during immobility. Each session contained only a limited number of immobility periods, resulting in sparse spatial coverage. Consequently, it is not possible for example to reconstruct immobility tuning curves for individual cells (this point is also further clarified in response to the comment 1 of Reviewer #3).

Regarding the logic of the data presentation, the overall storyline is currently as follows:
Figure 1 illustrates the experimental approach.
Figure 2 presents the behavioral segmentation (*active vs. quiet immobility*) and spatial coding (population level analysis).
Figure 3 addresses plasticity and single-cell stimulation.
Figure 4 focuses on theta oscillations, with analyses centered on *active immobility*, where theta activity is observed.
Figure 5 provides a deeper view into the population-level findings presented in **Fig. 2** by examining spatial coding across *active and quiet states*, highlighting cellular heterogeneity.

We have explored alternative ways of organizing the figures, but we believe that the current storyline best represents the data and guides the reader through the findings from a general overview to more specific insights. We have streamlined and improved transitions in the text and incorporated new data and analyses, and we hope that the Reviewer finds the revised logic and flow appropriate.
Moreover, to better link the data (and figures) into a cohesive story, we have also performed additional analysis. For example, we show that behavioral modulation of place cells is gated by spatial location (**Fig. 2d**), thus linking more directly spatial coding to behavioral modulation. We have performed additional analysis on theta oscillations (for details, see response to comment 13 of Reviewer #2 and comment 4, 12 of Reviewer #3) and provide new analysis on the behavioral correlates (thus linking more directly theta to behavioral modulation). In addition, we performed decoder analysis (now shown in **Fig. 2g, Fig. 5j** and **Supplementary Fig. 2**) thus more directly linking spatial coding to behavioral state as well as to cellular heterogeneity (**Fig. 5j, Supplementary Fig. 2**).
As for the run curve, as requested by the Reviewer, it is now shown in **Fig. 2e**, on top. Indeed, as anticipated by the Reviewer, the shape of the immobility place field differs from that observed during running; for example, the immobility field appears slightly forward-shifted relative to the peak of the run field, which may indicate potential "prospective coding" (we have conducted additional analyses along these lines, examining reactivations during ripples, see comment 3 of Reviewer #3). However, due to the limitations discussed above – specifically, that we cannot systematically sample all immobility locations along the belt for every cell – this curve can only be constructed as a population average (for details, refer to the revised section 'Analysis of place cell firing during awake immobility' of Methods). Therefore, we consider the conclusions that can be drawn from this comparison limited and have opted for not referring to these differences in the revised manuscript.

CHANGE: To address the Reviewer's comment, we have improved the flow of the text and strengthened the connections between figures. We have more clearly specified the limitations of our work, including the inability to compute immobility tuning curves on a cell-by-cell basis (now noted in the revised Results, page 5, lines 106-107 and Methods, page 4, lines 161-162). We have complemented our analysis with a Bayesian decoding analysis (for a comprehensive description, please refer to section '**A3. Bayesian decoding analysis**' of the **Addendum**). The run curve is shown in **Fig. 2e**.

4.1. There is no population level analysis in the paper. What do these different populations of cells tell us during the different behavioral states? How good is the place decoding in these cell populations (behaviorally modulated and non-modulated) during Run, Active immobile, and Quiet immobility?
COMMENT: We thank the reviewer for this great suggestion (see also related comments, comment 3 of Reviewer #1 and comment 1 of Reviewer #3). In the revised manuscript, we decoded the spatial location during immobility with a Bayesian population decoding algorithm (for a comprehensive description, please refer to section '**A3. Bayesian decoding analysis**' of the **Addendum**). We thank the Reviewer for this excellent suggestion, which allowed us to strengthen an important aspect of the work, namely: (i) that place cells retain spatial coding during immobility, (ii) that the spatial code is better preserved during active immobility, and (iii) behaviorally modulated cells selectively contribute to spatial coding during immobility.
CHANGE: The decoding analysis is now presented in the new **Fig. 2g, Fig. 5j** and **Supplementary Fig. 2**, and referred to in the revised Results (page 5 lines 119-129, page 9 line 243-253).

4.2. How well can we predict whisker movement energy just by seeing the population activity? The paper is thin on experiments and this axis could help to beef the analysis up.
COMMENT: We thank the Reviewer for this suggestion. We would like to note that, in the present manuscript, our goal was to partition behavioral states in a simple binary manner based on changes in a single variable (whisker-pad motion). In other words, we did not use motion energy as a continuous variable for the current analyses, but rather to classify our data into two discrete behavioral states. We believe this represents a necessary first step toward revealing meaningful differences and characterizing hippocampal dynamics across distinct arousal states. The approach suggested by the Reviewer would require a more comprehensive analysis framework in which whisker motion energy is used as a continuous predictor (or predicted variable). While we fully agree that such an approach would be highly informative, it would substantially expand the scope of the present work and therefore we feel it would be better suited for a dedicated follow-up study. We hope the Reviewer agrees with our assessment.
CHANGES: none.

Here are some more detailed comments on the Figures:
Figure 1
5. There is no legend for the heatmap in panel c.
CHANGE: We added the color range in the corresponding figure legend.

6. In the example shown in Figure 1b, there are obvious moments in the "Run" periods where the animal transiently halts locomotion, moves its whisker pad, constricts its pupils, and LFP power is more spread… so why are they included in "Run"? I suppose these events were too short to be called immobility but that does not mean they should automatically be called Run, as the animal is not running.
COMMENT: We thank the Reviewer for the comment and apologize for the misleading display. Running epochs included only periods during which the animal was moving (speed > 1 cm/s); occasional pauses in

locomotion were therefore excluded from the "run" analysis. To avoid any misunderstandings, we have corrected the annotation in the figure, clarified this explicitly in the corresponding figure legend.
CHANGE: We have changed the annotation in **Fig. 1b**, showing now only running epochs.

7. *"During immobility, LFP activity was more heterogeneous, characterized by low-frequency activity as well as bouts of theta epochs (Fig. 1d)…"*. Epochs or bouts are not evident in Figure 1d, which is a power spectrum so does not show "bouts" of anything. I do not see theta bouts either in Figure 1b, but maybe a zoom in on a particular bout could highlight what the authors refer to ? Also, I do not understand what heterogeneous LFP means. There are more pronounced peaks in the Run data for low freqs, theta, and beta/sigma, as well as higher gamma in general… whereas the power spectrum is smoother for immobility. I am not sure that "smoothness" is the same as heterogeneity.
COMMENT: We thank the Reviewer for this comment. We acknowledge that the term "heterogeneous" is vague and not rigorously appropriate when referring to LFP. What we intended to convey is that LFP activity exhibits a broader distribution of power across frequencies during immobility, in contrast to the more theta-dominated power increase observed during running periods (**Fig. 1d**). We have therefore removed "heterogeneous" from the text.
To address this Reviewer's comment, we have also quantified LFP oscillations in different frequency bands (e.g. theta, gamma, delta, ripple) and we show now these quantifications (with statistical comparisons) in **revised Fig. 2c**. We note that for theta oscillations and ripple oscillations, we have further refined our analysis (following the Reviewers' comments) and performed additional analysis; for details, we refer the Reviewer to sections '**A1. Revised theta oscillations analysis**' and '**A2. Improved visualization and quantification of theta oscillations**' of the **Addendum** at the end of this document.
We thank the Reviewer for this comment, which gave us the opportunity to perform additional LFP and theta-related analysis, which in our view substantially strengthens our study.
CHANGE: We have reworded "heterogeneous" in the revised results (page 4 lines 75-76) and we have added LFP quantifications in revised **Fig. 2c**. For more details, see details in **Addendum A1 and A2.**

Figure 2
8. *"…phase being characterized by immobility-related LFP theta activity (Fig. 2b). "*. This graph is an inset in Figure 2a…
COMMENT: We thank the Reviewer for the comment. We have now revised the corresponding figure (for details, see section '**A2. Improved visualization and quantification of theta oscillations**' of the **Addendum** at the end of this document), and immobility-related LFP changes are now shown in the new **Fig. 2b**.

9. *"On the other hand, sharp-wave ripples were exclusively observed during 'quiet' immobility (Fig. 2 and Extended Data Fig. 2), thus further supporting the binary brain state classification."*. This is not supported by the data presented. In Figure 2 itself, there is a filtered LFP in the ripple band (150-250) Hz that shows a single SWR that occurred in the "Quiet" state, not sufficient evidence to support this claim. In the Extended data Fig. 2, the SWR rates in "Active immobility" state are not 0, and further, the time when the cells show the highest levels of spiking at the start of the "Active" behavioral state, SWRs are not measured due to artifacts. The authors need to either change this statement or show an analysis that supports their claim that ripples occur exclusively during "Quiet" immobility.
COMMENT: The Reviewer is right and raised an important point, which prompted us to perform additional analysis on ripples. Ripples are notoriously difficult to detect, and multiple detection algorithms and methods have been proposed in the literature[30]. A critical challenge is differentiating *bona fide* ripples

from spontaneous "artifacts" (typically induced by muscle twitches or alike[30]) that contaminate the corresponding frequency bands and can appear ripple-like upon qualitative examination. To address this limitation, we adopted a rigorous approach by defining ripples based on concomitant co-activation of principle neurons. In the previous version of the manuscript, we had (erroneously) applied these criteria only to putative ripples in the active state; in the revised manuscript, we have applied these criteria to all putative ripples, regardless of the behavioral state in which they occurred. This correction improved the detection of ripple rates around the onset of whisker-pad motion. As the Reviewer can see in the **new Fig. 2b** – and consistent with our previous observations – ripple rates decrease following the onset of whisker-pad motion, as expected since theta and ripple states are non-overlapping hippocampal network states[31]. Since ripple occurrence is not reduced to absolute zero (contribution of biological and technical variability is difficult to assess), we have refrained from categorical statements and removed the term "exclusively," as correctly suggested by the Reviewer. We also note that this refinement of the ripple detection algorithm did not affect the ripple-associated entrainment reported in the previous version of the manuscript. These data are now presented in **Supplementary Fig. 3f,g**, along with a more rigorous interpretation based on downsampling analysis (for details, see response to comment 18 of Reviewer #2).
Last, we would also like to note that we have performed a decoding analysis on ripples-related reactivations. This analysis is now shown in **Supplementary Fig. 3d,e** (for more details, the Reviewer is referred to the response to comment 3 of Reviewer #3).
CHANGE: the results of this analysis are now shown in revised **Fig. 2b,c** and removed the term "exclusively", changing the sentence to "*Sharp-wave ripples predominantly occurred during 'quiet' immobility (Fig. 2b,c), further supporting the binary brain state classification.*" (page 5 lines 90).

10. Panel 2b the heatmaps do not highlight the potential differences. The warmth scale is too broad and the differences are not easy to appreciate, also the frequency range is much broader than it needs to be (theta/delta seem to be the important ones). Consider altering the scale and/or creating a third "difference" heatmap. Theta/Delta score is never statistically quantified so we are left wondering if these differences are significant.
COMMENT: We thank the Reviewer for suggesting this refinement. In the revised manuscript, we have improved the display and added the quantification to the triggered spectrogram (we refer the Reviewer to section '**<u>A2. Improved visualization and quantification of theta oscillations'</u>** of **Addendum** at the end of this document).
CHANGE: we have refined the analysis and improved the display of these data in the revised **Fig. 2b.**

11. ***"To explore whether CA1 place coding is preserved during periods of immobility, we analyzed all immobility periods within and outside the cells' place fields (n=166 place cells in field, n=164 place cells out of field). We found that on average, place cells fired at higher rates when animals were kept stationary within the cell's place field, as compared to outside, irrespectively of the behavioral state (firing rates normalized to run periods; active immobility: in field, 0.38 ± 0.29, out of field, 0.18 ± 0.21, post-hoc comparison p=1.02e-16; quiet immobility: in field, 0.24 ± 0.17, out of field, 0.18 ± 0.16, post-hoc comparison p=8.67e-4; p=4.23e-19, Kruskal-Wallis test; Fig. 2c,d). Consistent with the behavioral modulation data (Fig. 2b), place cell activity was highest in the 'active' state (Fig. 2c,d)."*** **There were 340 place cells in Fig 1. What happened to them for this part where there are only 166? Panels c and d are not enough. The authors must show the full rate maps (like Fig 1c) for Run, Active immobile, and Quiet immobile, normalized to peak for all cells that this data exists for. Not sure if it is just misalignment in the figure, but the peaks of the immobility curves in Fig 2c are not aligned with the peak for the red curve above from the Run.**

COMMENT: We thank the Reviewer for giving us the chance to clarify this point (see also response to related comment 3 of Reviewer #2, comment 1 of Reviewer #3). The reviewer can find an overview of our dataset in the **Addendum** (see section '**A5. Dataset overview'** at the end of this document), and a concise version is now reported in the revised Methods (see 'Dataset overview' of Methods). We would like to note that an inherent limitation of our experimental design is that we do not have systematic sampling across all belt locations during immobility. Each session contained only a limited number of immobility periods (ranging from 1 to 9; see revised Methods), resulting in sparse spatial coverage. Consequently, it is not possible to reconstruct immobility tuning curves for individual cells. Therefore, spatial tuning during immobility can only be represented as a population average (for details, refer to the revised section 'Analysis of place cell firing during awake immobility' of Methods). This is what is shown in **Fig. 2e** and **Fig. 5g**, which was intended for display purposes, as all quantitative analyses were performed independently of this population tuning curve.
As for the apparent "misalignment" of the peaks in run and immobility (see also our response to related comment 3 above): indeed, the immobility field appears slightly forward-shifted relative to the peak of the run field, which may indicate potential "prospective coding" (we have conducted additional analyses along these lines, examining reactivations during ripples, see comment 3 of Reviewer #2). However, due to the limitations discussed above – specifically, that we cannot systematically sample all immobility locations along the belt for every cell – this curve can only be constructed as a population average by combining all recordings. Therefore, we consider the conclusions that can be drawn from this comparison limited and have opted not referring to these differences in the revised manuscript.
CHANGE: We have added a "Dataset overview" paragraph in the revised methods (page 9 lines 386). More details in the **Addendum** (see section '**A5. Dataset overview'** at the end of this document). The point referred by the Reviewer is referred to in the revised Results (page 5 lines 104-115) and Methods (page 4 lines 156-189) where we better clarify our data and how immobility population tuning curves are computed.

12. ***"In a subset of stimulated cells (8 out of 34), evoking spiking during active immobility was sufficient to create a de-novo place field at the stimulus location (Extended Data Fig. 3) "*. This experiment lacks a control. The sound that is used to encourage active immobility is a cue that itself could induce plasticity or activity. The authors need to perform sound cue only controls without micro stimulation. This will answer 2 questions, does the sound evoke activity itself? Does the microstim drive the plasticity and is this plasticity more than we see just with the sound? (8/34 is infrequent and maybe this can be explained simply by an unfamiliar sound cue at a particular location)**
COMMENT: We thank the reviewer for suggesting this control experiment (see also related to comment 11.2 of Reviewer #1, comment 2 of Reviewer #2 and comment 5 of Reviewer #3). To address the Reviewer's concern, we conducted additional sound-only control experiments, which are comprehensively descripted in the **Addendum** (see section '**A4. Sound stimulation controls'** at the end of this document). We thank the Reviewer for this comment, as it allowed us to strengthen this aspect of the study. Following this and the suggestion of the reviewer (see comment 2 of Reviewer #2), we have included these data in the main manuscript as **Fig. 3.**
CHANGE: The results of the new experiments analysis outlined in the **Addendum** (section section '**A4. Sound stimulation controls'**) is now shown in the new **Fig. 3** and referred to in the Results (page 6 lines 138-150).

Figure 3

13. *"We found that, compared to running, individual theta bouts during immobility showed a larger variability in frequency and reduced power "*. It is not clear why the authors are calling these "theta" bouts. They are 1-second periods after the onset of Active whisker pad movements during immobility. That is not a "theta" bout per se, as it is defined by behavior that may or may not be associated with high or low theta (not properly quantified either in Fig. 2b). To be a "theta" bout, it would be a period of high theta activity that is detected based on theta power… The authors specify that running "theta" bouts were sampled uniformly from mobility periods, and this could be incorrect. The authors need to verify that Active immobility is uniformly distributed within immobility periods, otherwise their sampling should match whatever distribution this yields. Active immobility periods might be clustered together or they might be biased towards onset or offset of immobility. The standard approach to this is to match inter-event-intervals (maintain the IEIs but shifted during the mobility periods) instead of choosing times from a uniform distribution.

COMMENT: We thank the Reviewer for the suggestion. To address this comment, we have implemented a theta segmentation analysis during immobility and reanalyzed findings related to theta oscillations (now presented in **Fig. 4**). For details on the revised analysis of theta oscillations, please refer to section '**A1. Revised theta oscillations analysis**' of the **Addendum** at the end of this document. This more rigorous re-analysis is fully consistent with our previous findings, further strengthening the conclusions and enhancing the rigor of the study. We thank the Reviewer for this comment.

Regarding the reviewer's suggestion to match inter-event intervals between active immobility and running epochs, we note that there is no systematic temporal relationship between these periods; however, we followed the Reviewer's suggestion and implemented this in the analysis (as described in section 'Theta oscillations segmentation and analysis' of the revised Methods), and it did not affect the results.

CHANGES: the results of the above analysis are now shown in the revised **Fig. 4a**. We updated the Methods referencing the changes in the analysis suggested by the reviewer (page 6 lines 242-246). See details in section '**A1. Revised theta oscillations analysis**' of the **Addendum** at the end of this document.

14. For panels c-h, the authors divide the dataset into 3 arbitrary groups of Active whisker movement durations of unequal sizes. In general, arbitrary divisions as such can be useful for visualization but not for statistics. They should not base their statistics on these groups but rather on the correlations between duration and the various measures. They should also standardize these measures so they can be applied to all durations in the same way (variable window sizes is not good). They make two claims that need clarification and better analyses:

COMMENT: We thank the Reviewer for the suggestion. We have (i) re-analyzed our data using regression models (**Fig. 4f,h**) and (ii) for visualization purposes, the data were grouped and displayed in quantiles (**Fig. 4d,e,g**). For details on these two points, please refer to section '**A1. Revised theta oscillations analysis**' of the **Addendum** at the end of this document. Altogether, this improved regression analysis confirmed our previous observations; namely, that whisker-pad motion duration correlated with both the frequency and the power of theta oscillations during active immobility, as well as with interneuronal firing rates and rhythmicity.

We also would like to point out that our previous LFP analyses (as well as current analyses) were not using a variable window size, but rather a fixed window to try to have a more comparable quantification between epochs. Nevertheless, we agree fully that current display in quantiles is more 'objective' and appropriate.

CHANGE: we use regression analysis (and for display, quantile groups) in the revised **Fig. 4f,h.** See details in section '**A1. Revised theta oscillations analysis**' of the **Addendum** at the end of this document

15. ***"We found that the duration of whisker-pad motion events correlated with the magnitude of pupil dilations (Fig. 3c) and was hence predictive of the arousal level of the animal ".*** **Here, the shorter Active whisker movement events are perfectly aligned with the longer events in terms of pupil dilation (see 3c bottom, before 1 second), and the dilation only separates once the shorter events have finished. So, pupil dilation follows the same trends in all three duration groups. For longer durations, there is more time to dilate … I would conclude that arousal as measured by pupil dilation builds with time spent actively immobile, with active immobility defined by whisker movements. I find the authors' claim stated above imprecise… it does not specify that this effect builds over time, as their text only refers to magnitude without considering the extended time window associated with longer active immobility events.**
COMMENT: We agree with the Reviewer's point that including the influence of time results in a better description of the result. We have thus rephrased the text accordingly.
CHANGE: To address this Reviewer's comment, we now state in the revised Results (page 7 lines 168-171): "*We found that the duration of whisker-pad motion events correlated with the magnitude of pupil dilations (Fig. 4c-d); hence, in comparison to the slower pupil dynamics and their gradual buildup over time (Fig. 4d), whisker-pad motion provided a more rapid and temporally precise readout of arousal.*"

16. ***"Interestingly, the frequency and relative power of theta oscillations also correlated with these behavioral metrics, with theta oscillations becoming progressively faster as a function of the duration of spontaneous whisking events ".*** **Here, they are comparing LFP power spectra from time windows of different sizes. They should redo this analysis at the spectrogram level to try and understand the dynamics of the theta power across Active immobility periods. If the theta frequencies are already faster in the early parts of the event and predict a longer active period, that is different (and perhaps more powerful) than if the theta frequencies accelerate as active whisker movements perpetuate. They could look at the first 0.5 seconds and the last 0.5 seconds of the spectrograms to see this, or only compare the last 0.5 seconds across all events. They could also quantify how long it takes the pupil to stabilize… something they refer to as "steady state" of 1.5 seconds in the methods but never explain or justify.**
COMMENT: We thank the Reviewer for the great suggestion. To address this point, we performed an additional analysis examining the temporal dynamics of LFP theta frequency across different quantile groups of whisker-pad motion durations. Consistent with the Reviewer's intuition, we found that the average theta frequency progressively increased after the onset of whisker-pad motion events of varying durations (see new **Supplementary Fig. 4d**). Notably, these temporal dynamics of LFP theta oscillations were paralleled by similar patterns in the activity of FS interneurons (as shown in **Supplementary Fig. 5c,d**). Interestingly, FS interneurons exhibited a phasic activation at the transition to active immobility, which remained relatively invariant across whisking durations, followed by a more sustained component that gradually increased as a function of whisking duration – thus mirroring the dynamics of LFP theta oscillations (**Supplementary Fig. 4d, Supplementary Fig. 5c,d**). Altogether, the LFP analysis demonstrates that theta frequency increases gradually over the course of a whisker-pad motion event, a finding further supported by the concomitant and sustained increase in FS spiking rates.
Additionally, we also improved the spectrogram showed in former **Supplementary Fig. 4** by dividing whisker-pad durations in quantiles, and added a quantification of the power changes in different frequency bands (theta, gamma, delta; see **Supplementary Fig. 4e,f**).
We also apologize for not having explained the rationale behind our choice of 1.5 s as the minimum duration defining "steady-state" active immobility. Following the rationale of previous studies[32–34], to ensure the analysis focused on stable network dynamics, we restricted it to steady-state periods of active immobility, defined as events longer than 1.5 s. This threshold was derived from the firing dynamics of putative fast-

spiking interneurons, which more closely track whisker-pad motion. As shown in the revised **Supplementary Fig. 5c,d**, interneuron firing rates exhibited an inflection ("kink") around 1.5 s after motion onset, beyond which the firing rates increased more gradually (the corresponding threshold is now indicated by a dotted line in the figure).
CHANGE: the results of the above analysis are now shown in the new **Supplementary Fig. 5c,d** and **Supplementary Fig. 4d.**

<u>**Figure 4**</u>
17. The "modulation" index is not correctly defined. Modulation can be either positive or negative. The authors only use the upper boundary of the confidence interval which suggests they only looked for cells that are positively correlated with Active whisker pad movement. In Fig. 4e, it is shown that some neurons have negative correlations at peak, so for any "modulation" index, the statistical test should be extended to see if these cells are significantly anti-correlated with the whisker movements. This will also fix the problem in Figure 4c that z scores are only positive… which is because they ignore the negatively modulated cells (however they seem to be fewer and less strongly modulated).
COMMENT: We thank the Reviewer for the suggestion. The Reviewer is right in that our classification criteria primarily focuses on the detection of positively modulated cells. Specifically, place cells were defined as *positively* modulated if the cross-correlogram peak crossed the upper boundary of the 99% confidence interval based on the null distribution (n = 81 out of 166), while the remaining cells were classified as *non-positively* modulated (n = 85 out of 166). This is because only a very small subset of cells (n=4) within the non-positively modulated group met the criteria for negative modulation (i.e., a cross-correlogram trough crossing the lower boundary of the confidence interval). Because this subset is minimal, we focused our analysis on the main effect (which is positive modulation) but we refer to this point (and refined classification criteria) in the revised manuscript**.**
CHANGE: the above point is referred to in the revised Results (page 8 lines 211-214) and Methods (page 5 lines 206-210).

18. ***" However, during immobility, modulated neurons tended to fire at higher rates (average firing rates in field during awake immobility; modulated, 4.96 ± 3.88 Hz; non-modulated, 2.73 ± 3.34 Hz; p=1.04e-06, Wilcoxon rank-sum test; Fig. 4d) and to be preferentially recruited during awake sharp-wave ripple oscillations (Extended Data Fig. 2)".*** **The modulated neurons were defined as having a higher than chance cross-correlation (a measure that is sensitive to firing rate) with whisker movements during Active immobility… then they are re-compared with non-modulated neurons that did not have significant modulation during Active immobility… but this time the comparison is during general immobility… this is circular statistics so not a valid test. The authors could compare the firing rates during quiet immobility because whisker movements (which were already tested for) cannot bias the results.**
COMMENT: We apologize for not having sufficiently clarified our cell classification procedures. The cross-correlation was computed over the entire period of immobility for both modulated and non-modulated cells (see section 'Behavioral modulation analysis' of Methods). As the Reviewer correctly points out, it was important to control for firing rate differences between these groups, and we have addressed this issue in two ways. First, we applied a shuffling procedure to estimate when cross-correlation peaks exceeded chance levels, which formed the basis for our distinction between modulated and non-modulated cells. Second, to verify that differences in firing rate alone could not account for the cross-correlation peak, we performed spike down-sampling to match firing rates between the modulated and non-modulated groups. After this procedure, the peak in the cross-correlation was still preserved in the down-sampled modulated group (see **Supplementary Fig. 6b**). Importantly, only after this classification we observed a clear

distinction in firing rate changes at the onset of whisker-pad motion in the modulated cells. Thus, while we agree with the Reviewer that cross-correlograms are sensitive to firing rates, our shuffling and spike down-sampling analyses confirm that positive modulation is not mere consequence of rate differences.
In line with the Reviewer's comment, we also show comparisons of firing rates during active versus quiet immobility (**Fig. 5h**). We agree with the Reviewer that, to some extent, the firing difference during immobility may not be a "finding" per se, but might be a correlate of our cell classification procedure. For this reason, we removed the panel related to this firing rate difference during immobility, and we clarified the rational for this comparison to Run; specifically, modulated and non-modulated cells exhibit *similar* excitability during running (i.e., comparable firing rates), despite the *different* rates during immobility. This observation is paralleled by our new decoding analysis, which shows that the degree of behavioral modulation does not affect decoding during running, but is positively correlated with decoding accuracy during immobility (**Fig. 5j**). Thus, we believe that, despite the potential for a circular argument – which we addressed using downsampling and shuffling procedures – a comparison to the running condition remains meaningful. That is, examining running alone would not reveal any differences between the cell classes; the differences become apparent only during immobility (see lines 215-221 of revised Results), and in particular during active immobility (**Supplementary Fig. 2d**). We have reorganized **Fig. 5** and the corresponding text along these lines.
CHANGE: the above point is referred to in the results (page 8 lines 215-221) and the analysis referred to above is shown in the revised **Supplementary Fig. 6b.**

For SWRs, the authors have to do the above test properly. If the modulated neurons do have higher firing rates during quiet immobility as well, we would expect them to be slightly more active during quiet immobility ripples as well and this should be boiled into the test (which was not done) to see if they are indeed more recruited during ripples beyond what we would expect based on higher firing rates in general.
COMMENT: We thank the Reviewer for the suggestion to conduct spike down-sampling to verify the entrainment of the modulated and non-modulated groups by ripples (as also suggested by Reviewer #3 in comment 3). We had indeed speculated in the discussion that the higher recruitment of behaviorally-modulated cells by ripples might be due to their higher excitability during immobility. A spike-downsampling approach allowed us to directly test this assumption. Indeed, following downsampling, we found there was no difference in ripple entrainment between the two groups (see **Supplementary Fig. 3g**), indicating that the differential recruitment is a byproduct of differences in excitability. Based on these observations, we have reframed our descriptions accordingly in the Abstract, Results, and Discussion.
We thank the Reviewer for this comment, as it allowed us to gain a deeper insight into our observations.
CHANGE: the above analysis is now shown in the revised **Supplementary Fig. 3g** and referred to in the revised manuscript (results, page 8 lines 222-223).

19. ***"Notably, during active immobility, the hippocampal spatial representation was almost exclusively contributed by the subset of behaviorally-modulated place cells (Fig. 4f,g), indicating that the animal's location is primarily represented by the ensemble of behaviorally-modulated place cells.".*** **This is not shown. There is a larger bump for the "modulated" cells on the left in Fig 4f… but the place fields are still visible for quiet immobility and also for active/quiet immobility for non-modulated cells. Also, the between group comparisons in 4g just show that modulated neurons have higher firing rates during active immobility than all other cells/conditions. Again, not showing that the spatial representation "almost exclusively" is comprised of these cells. To show this, the authors have to use population approaches on spatial decoding and drop out the different populations (modulate/non-modulated). The spatial information could still be very well intact in all conditions.**

COMMENT: We thank the reviewer for the comment. To address this point, we have performed a decoding analysis, which is summarized in the **Addendum** (see section '**A3. Bayesian decoding analysis**' at the end of the document). In essence, we show that: (i) using a prior from both quiet and active immobility allows decoding of the animal's location during running, (ii) a prior based on active immobility results in better decoding, and (iii) there is a linear relationship between the degree of behavioral modulation and decoding performance.
For details, we refer the Reviewer to the response to the above comment 4.1.
CHANGE: The decoding analysis is now presented in the new **Fig. 2g, Fig. 5j** and **Supplementary Fig. 2**, and referred to in the revised Results (page 5 lines 119-129, page 9 line 243-253). See details in the **Addendum** (see section '**A3. Bayesian decoding analysis**' at the end of the document).

Minor corrections
20. There are many grammatical errors and not all are listed here … Some early suggestions:

1. **Abstract: This sentence has a grammatical error (parenthesis) and repetition problems (bracket):** ***"The hippocampal spatial code was [preferentially] (contributed by) a subset of behaviorally-modulated place-cells, which increased their firing upon transitions to active immobility and were [preferentially] recruited during awake ripple oscillations.".*** **Contributed by is used again in the main text of the manuscript and this does not work in the context it is used. Maybe use "carried by" ? (if the analysis that was suggested indicates this is true)**
2. **Line 5: … the phenomenology … has been …**
3. **Line 20 : … in correspondence with …**
4. **Line 49 : … to hippocampal dynamics …**
5. **Line 51 : … place cell activity…**

I stopped here and would suggest the authors get the manuscript proofread before resubmission.
CHANGE: We thank the Reviewer for spotting these mistakes. We have fixed them.

Reviewer #3:

Summary
Sartorato et al. capitalize on an experimental setup which allows "space-clamping" in order to study how hippocampal representations are modulated by behavioral state when the mouse is immobile. This question has remained quite understudied in the field, and is important in our understanding of hippocampal coding during immobility. Here, the authors use camera footage of the mouse's face to classify periods of behavioral arousal while the mouse is immobilized (ie. periods of active whisking). The authors find that place cells increase their firing rate during active whisking, an effect which is also accompanied by changes in theta power and frequency, suggesting that behavioral state drives distinct neural activity states in dCA1. Finally, the authors show that the increased cell activity during immobility emerges from roughly half of the recorded place cells whose firing rates are entrained to the whisking of the mice, indicating a distinct subpopulation of place cells that are behaviorally modulated. While the experimental question and approach are timely and interesting, the analysis presented here does not support the author's claims that "during awake immobility, the cognitive representation of location is active and dynamically modulated," as it focuses primarily on gross firing rate changes, which do not necessarily indicate a change in the cognitive representation of location. Furthermore, the results of the juxtacellular recordings described seem underwhelming and out of place, without further controls it is unclear what they tell us about the relationship between behavioral state and place coding. Therefore, the impact of this paper is unclear without additional analysis and experiments, which I outline below:
COMMENT: We are thankful for the positive assessment of our work, and for the constructive feedback. As the reviewer will see from the detailed responses below, we have performed additional experiments and analysis for rigorously addressing the points raised by the reviewer. This major revision effort has allowed us to strengthen the conclusions of our work, making the study more robust and coherent.

Major Concerns
1. The primary claim of the paper is that behavioral state modulates the cognitive representation of location during immobility. I do not think the analyses presented here entirely support this claim. The authors show two main results to support this conclusion: 1) place cell firing rates increase with active whisking during immobility, and 2) this firing rate increase was only present in about half of the recorded place cells. The observation that firing rate increases does not tell us much about the cognitive representation of location. If anything, many of the results of this paper indicate that place cells should maintain a fairly stable representation of location during immobility, regardless of behavioral state (for instance, the tuning curve in Fig 2C is only slightly changed, and standard place-coding properties are not changed between modulated and unmodulated cells such as place field size, bits/spike, and field sparsity in Extended Data Fig 6f,g; that said, there is clear gain modulation of place tuning in Fig. 4f). An analysis of the pseudo-place fields generated using the space clamping technique (Fig. 4f) would provide much more insight into the nature of spatial tuning during immobility. For instance, how the size, spatial information, and sparsity of the pseudo-place field are affected by behavioral state. Furthermore, to claim that the cognitive representation is modulated, I would be more convinced by Bayesian population decoding during the immobile state: Would decoding accuracy increase when the mouse is actively whisking while immobilized (as might be predicted by the tuning curve in Fig. 4f)? If so, what is causing the change in accuracy? Is the posterior of the decoder sharper or are there fewer non-local events that are common during immobilization (as suggested by Extended Data Fig. 2c). In the current draft, very few analyses seem

to directly quantify the representation of location (namely, how precise is the place code during immobility, and how is it affected by behavioral state).

COMMENT: We thank the reviewer for their constructive feedback. We also apologize for the lack of clarity regarding how tuning curves during immobility were computed (**Fig. 2e and Fig. 5g**, see also comment 3, 11 of Reviewer #2). An inherent limitation of our experimental design is that we do not have systematic sampling across all belt locations during immobility. Each session contained only a limited number of immobility periods (ranging from 1 to 9; page 1 line 32 of revised Methods), resulting in sparse spatial coverage. Consequently, it is not possible to reconstruct immobility tuning curves for individual cells. Therefore, spatial tuning during immobility can only be represented as a population average (for details, refer to the revised section 'Analysis of place cell firing during awake immobility' of Methods). This is what is shown in **Fig. 2e**, which was intended for display purposes, as all quantitative analyses were performed independently of this population tuning curve. Thus, estimation of spatial information, sparsity or size of the place field during immobility was not possible, as these metrics require uniform sampling of the environment[35,36].

To rigorously address this comment, we implemented a decoding analysis, which is described in the **Addendum** (see section '**A3. Bayesian decoding analysis'** at the end of this document). We thank the Reviewer for this excellent suggestion, which allowed us to strengthen an important aspect of the work, namely: (i) that place cells retain spatial coding during immobility, (ii) that the spatial code is better preserved during active immobility, and (iii) behaviorally modulated cells selectively contribute to spatial coding during immobility.

CHANGE: The decoding analysis is now presented in the new **Fig. 2g, Fig. 5j** and **Supplementary Fig. 2**, and referred to in the revised Results (page 5 lines 119-129, page 9 line 243-253). See details in the **Addendum** (section '**A3. Bayesian decoding analysis'** at the end of the document). The limitation of the experimental design is acknowledged the revised Methods (page 1 line 32).

2. The authors frequently mention gain modulation of place cell firing, but never actually quantify the gain changes within the tuning curves (at least beyond gross firing rate changes, which imply an additive effect that is not necessarily related to "gain" which I typically associate with a multiplicative effect – I bring up this point because an additive shift of the place field would not change the spatial information in the place field, only a multiplicative "sharpening" of the field would increase spatial information). To do so would be relatively easy: for instance, for Figure 2c, per cell, plot each point of the quiet curve against each point of the active curve and fit with a line: the slope would be the gain value and the intercept would be the additive firing rate shift. A similar analysis would likely reveal a large multiplicative gain change in modulated cells in Fig. 4f.

COMMENT: We thank the Reviewer for this comment. As suggested, we conducted a regression analysis comparing firing rates during quiet and active immobility for behaviorally modulated and non-modulated cells. We observed a multiplicative gain only in behaviorally modulated cells (i.e., linear model slope $\beta \neq 1$, $\beta = 1.56 \pm 8.12 \times 10^{-2}$ SE, $t(79) = 6.87$, $p = 1.34 \times 10^{-9}$) and not observed in non-modulated cells ($\beta = 1.05 \pm 3.29 \times 10^{-2}$ SE, $t(83) = 1.41$, $p = 0.16$). This analysis has been included in the revised **Fig. 5i** (see also **Reviewer Fig. 7**). We are grateful to the Reviewer, as this comment allowed us to add a new insight regarding the type of gain modulation observed in our data.

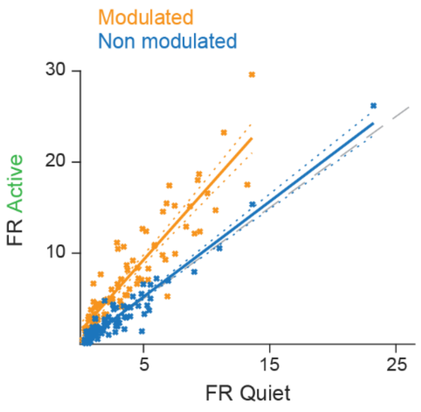

Reviewer_Figure 7. Multiplicative gain modulation in behaviorally-modulated cells.
Firing rate comparison between quiet and active immobility for behaviorally-modulated cells (top, test of slope $\beta \neq 1$, $\beta = 1.56 \pm 8.12e\text{-}2$ SE, $t(79) = 6.87$, $p = 1.34e\text{-}9$; linear model, $R2=8.23e\text{-}1$, $F(81,79)=3.68e2$, $p=1.84e\text{-}31$) and non-modulated cells (bottom, test of slope $\beta \neq 1$, $\beta = 1.05 \pm 3.29e\text{-}2$ SE, $t(83) = 1.41$, $p = 0.16$; linear model, $R2=9.24e\text{-}1$, $F(85,83)=1.01e3$, $p=2.71e\text{-}48$). Data shown in **Fig. 5i**.

CHANGE: The above analysis is included in revised **Fig. 5i**, and referred to in the revised results (page 9 lines 238-242).

3. Generally, the authors emphasize the SWR results, but it is unclear why they are relegated to a supplement. These results are interesting, and further exploration of decoded position during SWRs would provide further support that the cognitive representation of location is modulated by behavioral state. While authors observed a decrease in ripple rate during active immobility, they also saw that the behaviorally modulated place cells were more active and more likely to be reactivated during ripples (Extended Data Fig. 2g). These effects are interesting, and while this may be an artifact of having higher firing rates, they raise a lot of questions: Are there more non-local sequence events (see work from David Foster and Loren Frank) during active versus quiet immobile states (ie. forward/reverse replay)? Are the non-local events more likely to contain the modulated ensemble of cells? If so, are the non-local events preferentially representing particular locations, or particular types of replay (say, forwards versus backwards replay, or sequences extending ahead of or behind the mouse, which might suggest a role of behavioral state in consolidation versus prediction).

COMMENT: We thank the Reviewer for the suggestions. In line with comment 18 from Reviewer 2, conducted spike down-sampling to verify the entrainment of the modulated and non-modulated groups by ripples. We had indeed speculated in the discussion that the higher recruitment of behaviorally-modulated cells by ripples might be due to their higher excitability during immobility. A spike-downsampling approach allowed us to directly test this assumption. Indeed, following downsampling, we found there was no difference in ripple entrainment between the two groups (see **Supplementary Fig. 3g**), indicating that the differential recruitment is a byproduct of differences in excitability. Based on these observations, we have reframed our descriptions accordingly in the Abstract, Results, and Discussion.

Regarding the suggestions related to the analysis of replay sequences during ripples: unfortunately, differences in ripple reactivation between modulated and non-modulated cells could not be assessed directly. Such an analysis would require separate encoding models for these two populations, but the low number of co-recorded cells per session made this unfeasible. These limitations also prevented a systematic analysis of different types of replay and whether they are associated with a particular cell group. These replay-associated metrics are notoriously ‘challenging’, as they heavily rely on robust statistical assessment and large number of co-recorded cells[37,38].

To address the Reviewer’s comment in an alternative way, we reconstructed spatial location during ripple reactivations using a Bayesian decoding algorithm (see section ‘Bayesian decoding analysis’ of the revised Methods). Consistent with previous observations[39,40], we found that decoded locations during ripples tended to accumulate around the reward location (**Reviewer_Fig. 8b,c**, also reported in **Supplementary Fig. 3d,e**).

Furthermore, there was no systematic bias in the relative decoding position (ahead or behind the animal), indicating that ripple reactivations were not preferentially skewed toward positions in front of or behind the animal (see **Reviewer_Fig. 8d,e**).

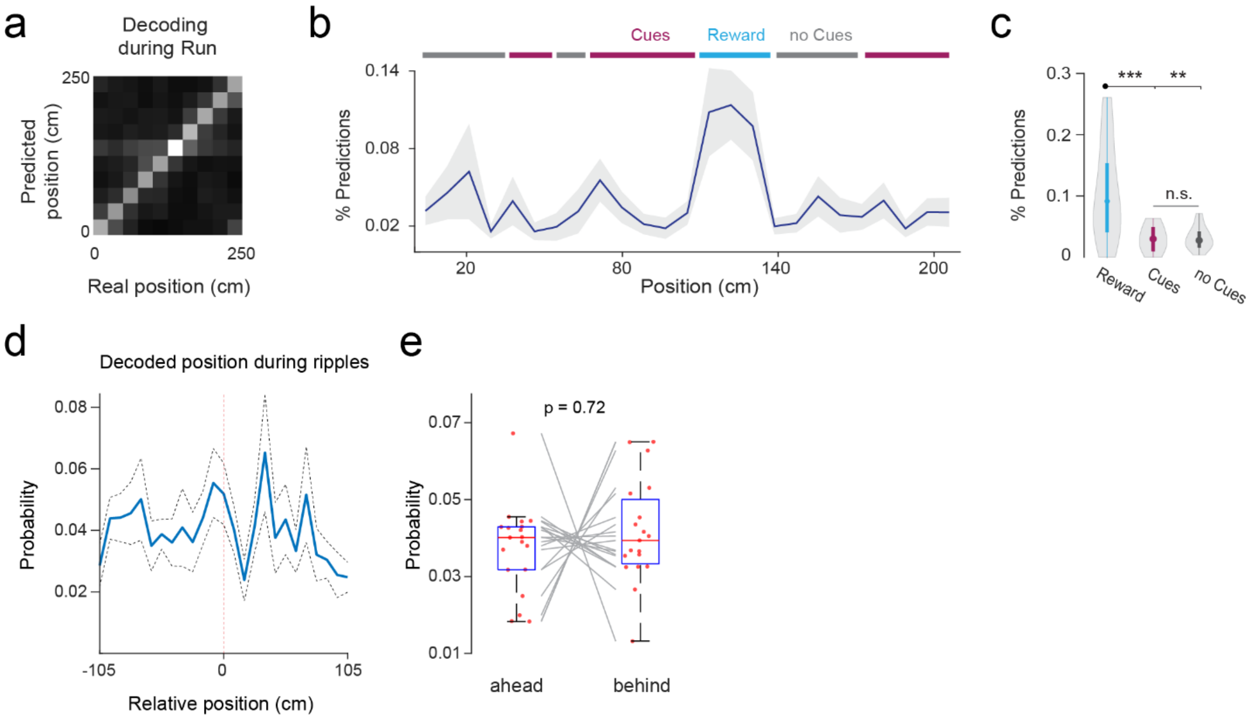

Reviewer_Figure 8. Reward location is selectively reactivated during sharp-wave ripples.
(a) Average confusion matrix showing the decoding accuracy during running epochs (n=19 sessions). The same decoders were used to reconstruct the offline location during SPW-R related reactivations (shown in (b,c)) (see 'Bayesian decoding analysis' in Methods). Also shown in **Supplementary Fig. 3c.**
(b) Average distribution of decoded locations during SPW-R-coupled reactivations (n=19 sessions). Top panel indicates different sections of the belt (blue, reward; purple, cues; grey, no cues). Error bars indicate SEM. Also shown in **Supplementary Fig. 3d.**
(c) Percentage of decoded locations quantified in different sections of the belt (same color code as in (d), n=19 sessions). p=2.00e-4, Kruskal-Wallis test; ** p<0.01, *** p<0.001, post-hoc comparisons. Also shown in **Supplementary Fig. 3e.**
(d) Same as (b) but as a function of the animal's position (positive values indicate positions ahead of the animal).
(e) Average percentage of decoded locations ahead and behind the animal's position (n=19 sessions), p=0.72, Wilcoxon signed-rank test.

In summary, while this approach is less direct than a full analysis of ripple sequences – which was not feasible given the available data – it indicates that reward locations are preferentially reactivated, without a systematic bias in relative position. These results are now included in **Supplementary Fig. 3** and referred to in the revised manuscript.
CHANGE: The above analysis is included in the new **Supplementary Fig. 3c-e** for the reward-related reactivation and in **Supplementary Fig. 2f,g** for the SPW-R down-sampling, and referred in the revised results (page 6 lines 131-135).

4. The result of an increase in theta power does not quite line up with the spectrograms in Fig. 2b. By eye, it looks like there is more low theta power in the quiet state than in the active state (dark red band near 5Hz). While I understand the quantification is using the theta/delta ratio, the delta ranges seem to include some of the typical theta range (in the discussion, Type-2 theta is defined from 4-7Hz, while the delta range used for the ratio overlaps with this – from 1-5Hz in the methods; previous

studies also use delta ranges closer to 2-4Hz, see Malezieux et al., 2020; Grosmark et al., 2012; Wikenheiser and Redish, 2014). Given that the main effect appears so close to 5Hz, do these results hold using typically defined ranges for theta and delta?
COMMENT: We thank the reviewer for the suggestion (see also related comment 10 of Reviewer #2). In the revised manuscript, we improved the display of the triggered spectrogram in **Fig. 2b** and added quantifications for delta, theta and gamma bands upon transition from quiet to active immobility (in **Fig. 2c**). To fully address the reviewer's concern, we used typically defined and non-overlapping ranges for theta and delta (theta: [6, 10] Hz; delta: [0.5 4] Hz). Details on the improvement of the triggered spectrogram can be found in the **Addendum** (see section '**A2. Improved visualization and quantification of theta oscillations**' at the end of this document).
CHANGE: To address this comment, we have improved the display of **Fig. 2b** and added a quantification in **Fig. 2c.** For more details, see **Addendum** (section '**A2. Improved visualization and quantification of theta oscillations**' at the end of this document

5. The juxtacellular recording results are somewhat underwhelming and difficult to interpret without further control experiments. While they do demonstrate that it is possible to induce a place field during active immobility, without further context this does not say anything about behavioral state modulation of the hippocampal code. For instance, is it equally likely to cause place field formation during quiet immobility (the same experiment without the auditory tone)? Is stimulation more effective in behaviorally modulated cells during active immobility (as far as I can tell, it would be feasible to perform the cross-correlation analysis used in Figure 4 to determine whether the stimulated cell is behaviorally modulated)? The place field induction rate here is quite low (8/34) compared to the same experiment during running (14/15 cells in Bittner et al., 2015), which would suggest that, if anything, an immobile but active state reduces plasticity. As presented here, it is hard to understand what this result implies without some of the controls mentioned (ie. replicating Bittner et al during running, or comparing to induction success during the quiet immobile state – the latter seems like the ideal control in this case).
COMMENT: We thank the Reviewer for the comment. We agree with the Reviewer that the current juxtacellular stimulation dataset provides limited insight into the potential (i) cell-type specificity and (ii) behavioral state dependence of the observed plasticity. Specifically, we do not yet know whether behaviorally modulated and non-modulated cells differ in their plasticity, or whether these mechanisms are more effectively engaged during active versus quiet immobility. While addressing these questions would indeed represent a significant mechanistic advance, we believe they cannot be easily resolved within the scope of a revision due to the inherent methodological constraints of the juxtacellular recording/stimulation technique (see details below).
For instance, given the relatively small effect size of the juxtacelluar stimulation (i.e. observed in approx. 25% of the cells, see below), we anticipate a rigorous comparison between behaviorally modulated and non-modulated place cells would require a substantially larger dataset - likely more than double the current one - as it would be necessary to (i) record sufficient numbers of identified place cells, (ii) characterize their modulation during immobility within their place fields, and (iii) stimulate them under both behavioral conditions. Similarly, testing stimulation efficacy during quiet immobility – which we would predict to be lower than during active immobility due to the absence of theta oscillations and weaker spatial coding-would require real-time, closed-loop stimulation restricted to quiet immobility. This is particularly challenging for juxtacellular stimulation, which requires multiple current pulses to depolarize the cell[41], unlike intracellular stimulation methods that can induce single plateau potentials at precisely defined time points (as e.g. in Bittner et al., 2017 [23]).

In spite of the above limitations, we believe that – in agreement with the Reviewer's assessment - our current data demonstrate that place field induction can occur during immobility, and at comparable rates to previous reports during locomotion (e.g. [42]). We believe this is both novel and conceptually important, as current models typically consider locomotion a necessary component for engaging place-field plasticity.
Thus, to solidify our finding, we have conducted additional control experiments suggested by other Reviewers (see comment 11.2 of Reviewer #1 and comment 12 of Reviewer #2), increased our initial dataset, performed additional analysis, and improved the display of the data (see **revised Fig. 3**). Our current dataset now includes 44 juxtacellularly-stimulated cells (in 3 mice), as well as a control sound stimulation condition (80 cells from 2 mice). For more information about this control, please refer to the **Addendum** (section '**A4. Sound stimulation controls'** at the end of this document).
CHANGE: The above analysis is now presented in revised **Fig. 3**. For details, see the **Addendum** (section '**A4. Sound stimulation controls'** at the end of this document).

6. It is generally unclear how the space-clamp is implemented without digging into the methods. Because this is the key experimental manipulation in this paper, it is important to make this technique entirely clear. It should be explicitly explained in the main text that the mice are forcibly immobilized by applying a brake to the treadmill. I would also suggest a graphic that describes this in Fig. 1a.
COMMENT: We thank the Reviewer for the suggestion, and we apologize for the lack of clarity. We have now added the "brake" to the schematic in **Fig. 1a**, and we have better clarified our experimental procedures in the corresponding legend, in the results, as well as the methods section.
CHANGE: We included a description of space-clamping manipulation in the Results (page 4 lines 61-62) and Methods (page 1 lines 21-23) and added a "brake" to the schematic in **Fig. 1a**.

7. The authors should show histological traces of probes in hippocampus to verify the recording sites in dCA1.
COMMENT: Thanks for comment. We include below a representative histological image of a recording site labeled with a fluorescent dye (DiI, D12730, Invitrogen). We acknowledge that the post hoc images documenting the recording sites are of limited quality (as in the example below); however, the electrode track entering the CA1 pyramidal layer can be clearly identified. Since the available histological images are not of publication quality - and given the already extensive supplementary material - we would prefer not to include them in the revised manuscript.
We note that the location of the silicon probe across the CA1 pyramidal layer was also routinely verified according to electrophysiological criteria i.e. reversal of ripple oscillations (as shown in **Supplementary Fig. 3b**) as well as segmentation of units along the radial (deep/superficial) axis (and analysis that we have extended and refined in the revised manuscript; see **Supplementary Fig. 6f,g**).
CHANGE: none.

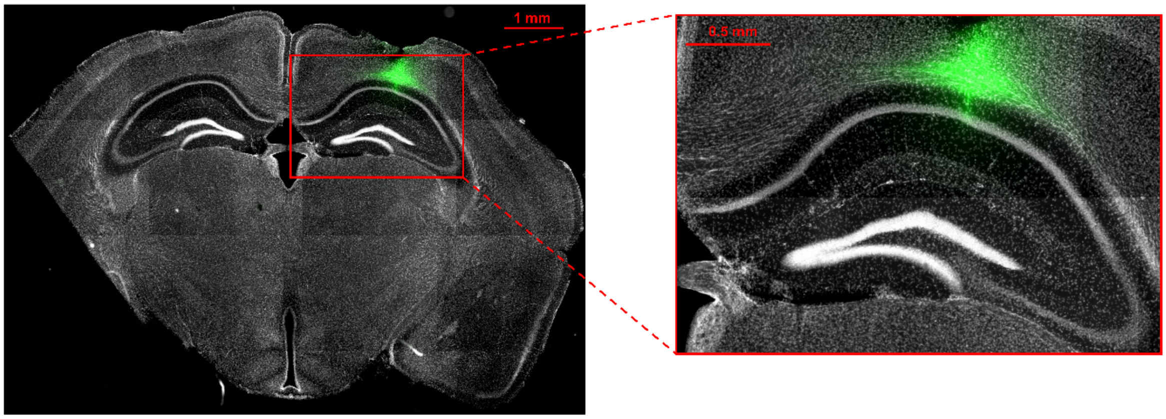

Reviewer_Figure 9. Histological trace in the CA1.
Left, coronal section of a mouse brain showing the probe trace in the CA1. Sections were stained with DAPI (white), and DiI is shown overimposed (green). Right, inset showing a higher magnification view of the histological trace in the CA1.

<u>Minor Concerns</u>
8. Possible grammar issue in the abstract. The sentence *"the hippocampal spatial code was preferentially contributed by a subset…"* sounds incorrect.
CHANGE: we have reworded the corresponding sentence to "*CA1 place cells continued to encode location during immobility, with the spatial code being primarily driven by a subset of behaviorally-modulated place cells (...)*".

9. In Figure 1d, middle, why is the peak of the running trace shifted off of zero if the data is z-scored to the running trace? Shouldn't that peak fall exactly on zero?
COMMENT & CHANGE: We thank the Reviewer for pointing this out. We fixed an alignment error in the code, and corrected the figure accordingly.

10. Line 100: n=166 place cells, but it is unclear how these cells were selected from the 340 place cells?
COMMENT: We apologize for not having sufficiently clarified our dataset (see also comment 11 from Reviewer #2). For a complete overview of our dataset, please refer to the **Addendum** (section '**<u>A5. Dataset overview</u>**' at the end of this document). Regarding the apparent discrepancy in "n": in total, we recorded 340 place cells. However, because not all belt locations could be systematically sampled during immobility (as noted above), only a subset of place cells had at least one immobility period within the place field (n = 166 out of 340 place cells), and only a subset had at least one immobility period outside the field (n = 164 out of 340 place cells). We have now clarified these aspects by also providing a better description of our dataset in the revised Methods (section '*<u>Dataset overview</u>*').
CHANGE: we have added a 'Dataset overview' paragraph in the revised methods (page 9 lines 386-405) and better clarified inclusion criteria in the revised Methods (page 4 lines 156-186) and in the Results (page 5 lines 103-113).

11. Fig 2c: n=131 cells, how were these selected from 166 cells referenced in the section of text (which says n=166 or n=164, then cites Fig. 2c at line 112)? Are the error bars computed over cells or immobility periods?
COMMENT: We apologize for not having sufficiently clarified our dataset and our inclusion criteria. For a complete overview of our dataset, please refer to the **Addendum** (section '**<u>A5. Dataset overview</u>**' at the end of this document).

CHANGE: In the revised Methods (page 4 lines 156-177) and Results (page 5 lines 104-108) and in the corresponding legend of **Fig. 2e** we better clarified the inclusion criteria for the calculation of population tuning curves during immobility. We have also added a 'Dataset overview' paragraph in the revised methods (page 9 lines 386-405).

12. Line 282: In the discussion, the authors say that theta oscillations occurred in short bouts, citing Figure 2a and Extended Data Figure 4, but "bout length" is never quantified or analyzed. This statement should be backed up by an analysis or reworded as a qualitative observation of the data.
COMMENT: We thank the Reviewer for this comment (see also comment 13 of Reviewer 2). We have performed a theta-epoch segmentation analysis and quantified several features, including the distribution of theta epochs length (now shown in **Supplementary Fig. 4b**). For a comprehensive description of the revised theta analysis, please refer to the **Addendum** (section '**A1. Revised theta oscillations analysis**' at the end of this document).
CHANGE: we have added the quantification of the duration of theta bouts in **Supplementary Fig. 4b.** For further details, please see the **Addendum** (section '**A1. Revised theta oscillations analysis'** at the end of this document).

13. Extended Data Figure 2b, in the caption the discarded traces are said to be orange but they are black.
CHANGE: Thanks for spotting this, we have fixed this mistake.

Addendum

Similar Reviewer comments are addressed collectively here below.

A1. Revised theta oscillations analysis

To address comments raised by Reviewers (comment 13 of Reviewer #2 and comments 4, 12 of Reviewer #3), we implemented theta segmentation during immobility, reanalyzed substantial portions of our data, and added new analyses. Specifically, we performed the following:

1. Theta-epoch identification and analysis.
2. Re-analysis of theta/whisker-pad motion relationship (**former Fig. 3d,e**, now **Fig. 4e,f**).
3. Re-analysis of putative FS interneuron activity (**former Fig. 3g,h**, now **Fig. 4g,h**).
4. Temporal dynamics of theta following the onset of whisker pad motion (**Supplementary Fig. 4d**).

These points are elaborated here below.

1) Theta-epoch identification & analysis.
To identify theta epochs during immobility, we first computed LFP spectrograms and derived the time-varying theta/delta power ratio (theta band: 6-10 Hz; delta band: 0.5-4 Hz). Theta epochs were defined as continuous epochs in which the theta/delta ratio exceeded defined thresholds (i.e. based on the distributions of theta/delta ratios during immobility and running periods), consistent with previously established criteria ([43–45]; see also Methods, section 'Theta oscillations segmentation and analysis'). For each identified epoch, we extracted basic properties including peak frequency, peak power, and duration, and recomputed all analyses presented in **Fig. 3** based on these segmented events.
To validate the accuracy of our theta segmentation, we conducted several additional analyses (**Supplementary Fig. 4a-c**). *First*, to confirm that the detected onsets corresponded to genuine increases in theta oscillatory activity, we computed trigger-averaged spectrograms across sessions aligned to epoch onset. As shown in **Supplementary Fig. 4a**, theta power markedly increased around epoch onset, which correlated with increased whisker-pad motion. *Second*, to further corroborate the temporal relationship between whisker-pad motion and theta activity, we computed cross-correlations between whisker motion amplitude and the theta/delta ratio during immobility. Across sessions, we observed a consistent peak near zero lag, indicating tight coordination between the two signals. *Finally*, to assess the fine temporal relationship between whisker movements and theta events, we computed peri-event histograms aligned to whisker-pad motion onsets (**Supplementary Fig. 4c**). Notably, theta onsets tended to cluster within ± 1 s of whisker-motion initiation (**Supplementary Fig. 4c**), indicating that their temporal relationship did not exhibit a consistent directionality (at least at the temporal resolution our video recordings and the spectrogram window). Thus, the coordination between theta oscillations and whisker-pad motion may reflect co-modulation by a shared underlying process - fluctuations in arousal - rather than a causal coupling. Together, these results validate our segmentation approach and further characterize the dynamic coupling between hippocampal theta activity and whisker-pad motion.

2) Re-analyses of theta/whisker-pad motion relationship (former Fig. 3d,e).
We followed the reviewers' recommendations and reanalyzed all data previously presented in **Fig. 3**, while preserving the overall structure of the original figure. The revised analyses incorporate two major methodological improvements.
First, in the updated **Fig. 4d, e**, and **g**, we replaced the previously used arbitrary duration bins of whisker-pad motion with quantile-based groupings, each containing an equal number of events. This data-driven

approach removes potential biases associated with pre-defined group boundaries, as correctly noted by the reviewers. The quantile representation more accurately captures the continuous relationship between whisker-pad motion duration and the spectral characteristics of theta oscillations, which likely reflect underlying variations in arousal state. To further substantiate this interpretation, we included a color-coded representation of pupil amplitude across all whisker-pad motion events, sorted by duration (**Fig. 4c**). This analysis shows a close temporal coupling between whisker-pad motion and pupil dilation, consistent with a shared modulation by arousal.

Second, the former **Supplementary Fig. 4** has been revised to display quantile-specific trigger-averaged spectrograms, aligned to theta onsets. This analysis shows spectral dynamics across distinct event categories (**Supplementary Fig. 4e,f**) quantified as power within the theta (6–10 Hz), delta (0.5–4 Hz), and gamma (70–90 Hz) frequency bands. These analyses reveal a graded modulation across quantiles, corroborating and extending the observations reported in the revised **Fig. 4**. Note that for the above analysis, quantiles were extracted using all whisker-pad events across subjects and sessions, thus representing the complete distribution of events (see Methods for details).

Third, we replaced the previous analysis by using identified theta epochs associated with whisker-pad motion events (see **Supplementary Fig. 4c**). In **Fig. 4a**, we present the distributions of frequency and power for detected theta epochs during immobility, compared to matched periods during locomotion (matched for both duration and inter-event interval) [of note: in line with Reviewer #2's recommendation, we recomputed theta frequencies based on spectrograms; while the results remained unchanged, this approach introduced a lower temporal resolution compared to the previous version of the figure]. Also **Fig. 4b**, which shows the spike entrainment of pyramidal neurons, has been recomputed on identified theta periods. In response to the Reviewers' suggestion, the new **Fig. 4f** presents a correlation analysis between theta frequency and power with whisker-pad motion duration (log scale). This updated analysis confirms, consistent with our previous findings, that both power and frequency of theta oscillations increase as a function of whisker-pad motion duration and provides a quantitative description of this relationship using a linear-log function.

3) Re-analysis of putative FS interneuron activity (former Fig. 3g,h).

As in the previous analysis, we corroborated the findings on LFP theta oscillations by examining the rhythmic firing properties of putative fast-spiking (FS) interneurons. In the revised manuscript, we refined this analysis as follows: for each individual theta epoch, we computed the population autocorrelogram of FS interneurons (**new Fig. 4g**) and improved the quantification of rhythmicity metrics by performing a Fast Fourier Transform (FFT) to extract both peak frequency and peak power (referred to as the Theta Index) (**new Fig. 4h**), allowing us to analyze their relationship with whisker-pad motion durations. Also here, a linear-log model was applied to describe the relationships presented in **new Fig. 4h**. We note that variability across recordings and the lower resolution of the autocorrelogram FFT may account for the relatively modest correlations observed; however, both correlations are statistically significant and consistent with the LFP analyses shown in **Fig. 4f**.

Altogether, this reanalysis of theta LFP dynamics and FS interneuron spiking is fully consistent with our previous results and further reinforces the conclusions of the study.

4) Temporal dynamics of theta following the onset of whisker pad motion.

As suggested by the Reviewers (see comment 16 of Reviewer #2), to further clarify the temporal dynamics of theta oscillations during active immobility, we divided whisker-pad motion events associated with theta into quantiles based on duration. We then computed trigger-averaged spectrograms around whisker-pad onsets and extracted power and frequency time series (see example in **Supplementary Fig. 4d**). This analysis shows that for each whisker-pad duration quantile, both theta frequency and power increase following the onset of whisker-pad motion (**Supplementary Fig. 4d**). These observations are in agreement with the Reviewer's intuition and provide support for our interpretation that whisker-pad motion duration can serve as an index of arousal level. We are grateful to the Reviewer for suggesting this analysis, which has strengthened the interpretation of our results.

A2. Improved visualization and quantification of theta oscillations (Fig. 2)

Following the Reviewers' comments (comment 10 of Reviewer #2 and comment 4 of Reviewer #3) we have improved the presentation of this figure by improving both the visualization and the quantification of brain-state transitions. Specifically, we improved the display of the triggered spectrogram in former **Fig. 2b** (presented in revised **Fig. 2b**) in two ways. First, in contrast to before, we included all behavioral state transitions in the triggered spectrogram. The prior display was restricted to transitions during immobility periods which included at least one place cell in the field; in the updated version, we now present all transitions and show the firing-rate traces in a dedicated panel (**Fig. 2d**). We believe that the revised presentation represents an improvement not only in terms of clarity and visualization, but also because it provides a more comprehensive description of brain state transitions and behavioral modulation by incorporating all available data. Second, we applied more stringent signal-to-noise criteria to ensure reliable detection of theta oscillations over low-frequency noise. This led to the exclusion of two sessions (n = 2 out of 34; see Methods) that did not meet this quality threshold, thereby affecting the session-averaged spectrogram. These refinements substantially improved the presentation of **Fig. 2b**, which is now supported by quantitative analyses and statistical comparisons of LFP activity across relevant frequency bands (see new **Fig. 2c** and **Supplementary Fig. 4e,f**). In the revised manuscript, brain state changes are quantified using well-established, non-overlapping frequency ranges for theta, delta, and gamma bands. (theta: [6, 10] Hz; delta: [0.5 4] Hz; gamma [70 90]). In addition, we provide a more comprehensive overview of LFP theta activity during behavioral state transitions, which we now present in the new **Supplementary Fig. 4d**. Specifically, we have refined our detection of theta events (see details in response to comment 13 of Reviewer #2 and comment 4, 12 of Reviewer #3) and show the distribution of durations of theta events in **Supplementary Fig. 4b**, along with their temporal relationships to whisker-pad motion (**Supplementary Fig. 4c**), and as a function of whisker-pad duration (quantiles; see **Supplementary Fig. 4d**).

A3. Bayesian decoding analysis

We thank the reviewers for suggesting the inclusion of a decoding analysis to further support our findings. In the revised manuscript, we assessed spatial information during immobility using a Bayesian population decoding algorithm, following standard procedures in the field ([27,28]; see section ‘Bayesian decoding analysis’ of Methods). This approach allowed us to reconstruct the animal’s spatial location across different conditions (running, active immobility, and quiet immobility), enabling a targeted assessment and quantification of the spatial code during immobility and across behavioral states. Decoding accuracy was restricted to immobility locations (**Reviewer_Fig. 10a**), and errors were compared to chance level using a shuffling procedure. This was implemented to factor out the variability across sessions in the number of cells and immobility periods, as well as in the location of immobility periods (see **Reviewer_Fig. 10b**, section ‘Bayesian decoding analysis’ in Methods).

Specifically, we addressed whether spatial information was present during immobility through two complementary approaches. First, by training on running epochs and using a ‘running’ encoding model, which is inherently spatial, we found that decoding the animal’s location during immobility was only possible during active and not during quiet states (see **Reviewer_Fig. 10c,d**). Second, to further quantify spatial information during immobility, we implemented the reverse approach: we trained encoding models on active and quiet immobility epochs and decoded the animal’s location during running. Consistently, the model trained on active immobility provided more accurate decoding of running location, whereas decoding with the quiet immobility model remained below chance (see **Reviewer_Fig. 10c**; these findings were confirmed with session-level analysis; see **Reviewer_Fig. 10d**) pointing to better preserved hippocampal spatial code during active than quite immobility.

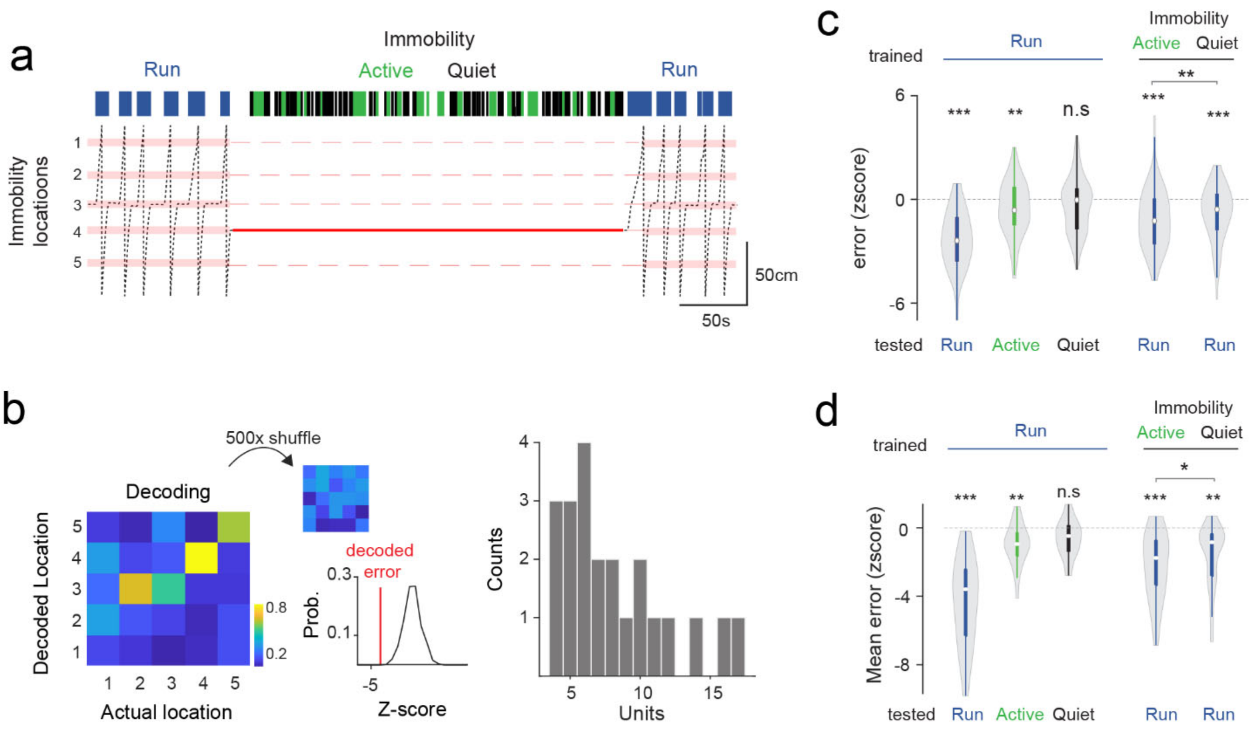

Reviewer_Figure 10. Bayesian population decoding during immobility.

(a) Schematic illustrating the decoding analysis in a representative session. Top: behavioral segmentation into running (blue), active immobility (green), and quiet immobility (black) epochs used in the analysis. Bottom: position trace (dashed black) with one immobility period highlighted by a solid red line; the other four immobility periods recorded in this session are indicated by red dashed lines. Training and testing during running were restricted to these five immobility locations, highlighted by red shaded rectangles (see details in Methods, “Bayesian decoding analysis”). Various training-testing combinations across active immobility,

quiet immobility, and running were conducted, as illustrated in (c,d) and in **revised Fig. 2g**. Also shown in **Supplementary Fig. 2a.**
(b) Left, confusion matrix showing decoding accuracies for the representative session shown in (a). In this example, training was conducted on active immobility and testing on running periods. Middle, errors (in cm) were z-scored with reference to a null distribution computed from 500 shuffled confusion matrices (an example is shown on top). The error normalization shown is at session-level; an analogous normalization procedure was conducted for immobility periods (for details, see 'Bayesian decoding analysis' in Methods). Also shown in **Supplementary Fig. 2b.** Right, numbers of cells used for the decoding analysis (n=22 sessions).
(c) Bayesian decoding errors using different combinations of training-testing behavioral windows (n=88 immobility periods, see also **Supplementary Fig. 2**). Labels indicate the behavioral periods used for training (top) and testing (bottom). Errors are z-scored with respect to a null distribution (see Methods). Trained run, tested run, p=1.27e-15; trained run, tested active immobility, p=1.92e-3; trained run, tested quiet immobility, p = 0.037; trained active immobility, tested run, p=1.86e-8; trained quiet immobility, tested run, p=3.10e-05; Bonferroni-corrected α=0.01, one-tailed Wilcoxon signed-rank test. Active vs quiet immobility encoding models, p=4.32e-3, Wilcoxon signed-rank test. ** p<0.01, *** p<0.001 after correction. Also shown in **Fig. 2g.**
(d) As in (c) but at session-level (n=22 sessions). Trained run, tested run, p = 2.15e-5; trained run, tested active immobility, p = 1.02e-3; trained run, tested quiet immobility, p = 1.06e-2; trained active immobility, tested run, p = 4.89e-05; trained quiet immobility, tested run, p = 2.01e-4; Bonferroni-corrected α=0.01, one-tailed Wilcoxon signed-rank test. Active vs quiet immobility encoding models, p = 1.36e-2, Wilcoxon signed-rank test. * p<0.05, ** p<0.01, *** p<0.001. Also shown in **Supplementary Fig. 2c.**

Additionally, **Reviewer_Fig. 11** shows that the average posterior probability during active immobility was sharper than during quiet immobility, even after excluding sharp-wave ripple events. Thus, the improved decoding performance during active immobility likely reflects enhanced spatial coding rather than being an artifact of reduced sharp-wave ripple incidence (and thus, of non-local reactivations).

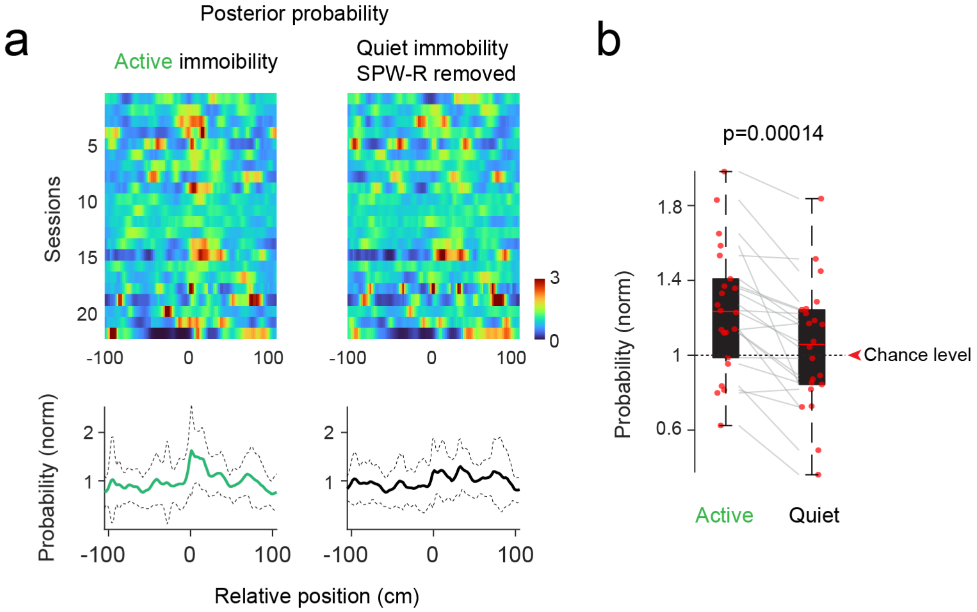

Reviewer_Figure 11. Posterior probability becomes sharper around the animal's location.
(a) Top, posterior probability over spatial location during active and quiet (non-SPW-R) immobility (n=22 sessions). Probabilities are normalized by dividing by the chance level (1/#spatial bins). Bottom, average posterior probability for active and quiet (non-SPW-R) immobility (n=22 sessions). Error bars indicate SEM.
(b) Average posterior probability during active immobility and quiet immobility around the animal's location (p=1.40e-4, Wilcoxon signed-rank test).

As a second step, we aimed to evaluate how the degree of behavioral modulation in individual cells relates to spatial coding during periods of immobility. To accomplish this, one straightforward approach could have been to train separately on the two cell groups - modulated and non-modulated - and compare their decoding accuracy. However, this was not feasible due to the low number of co-recorded behaviorally

modulated and non-modulated place cells. In fact, only 4 of 34 sessions met the minimum decoding criteria of ≥4 cells per training group (i.e. at least 4 modulated and 4 non-modulated cells; see Methods). To overcome these limitations, we used an alternative approach - specifically, a 'neuron dropping' procedure, previously employed in the literature to evaluate the contribution of individual cells to decoding (**Reviewer_Fig. 12;** [46–48]). In this analysis, we related the increase in the error (in the estimate of running location) caused by removing a single place cell from the training set with its behavioral modulation cross-correlation peak (see section 'Bayesian decoding analysis' of Methods for details). This approach allowed us to assess how behavioral modulation impacts spatial coding across different conditions by selectively testing encoding models derived from running and immobility. Importantly, while both the 'running' and 'immobility' encoding models successfully decoded the animal's location during running (see **Reviewer_Fig. 10c,d**), a positive correlation between the strength of behavioral modulation and decoding error was observed only for the 'immobility' model (**Reviewer_Fig. 12a**). This indicates that the more a place cell is behaviorally modulated, the greater its contribution to the immobility spatial code. Furthermore, when analyzing active and quiet immobility separately, a significant correlation was observed only for the 'active immobility' model (**Reviewer_Fig. 12b**), directly linking behavioral state to the hippocampal spatial code during immobility. The absence of a correlation between modulation strength and decoding error in the running model is consistent with the lack of firing rate differences (**Reviewer_Fig. 12a**) and spatial information differences (**Reviewer_Fig. 12c**) between modulated and non-modulated cells during running, emphasizing that behavioral modulation is a distinctive feature of the immobility spatial code.

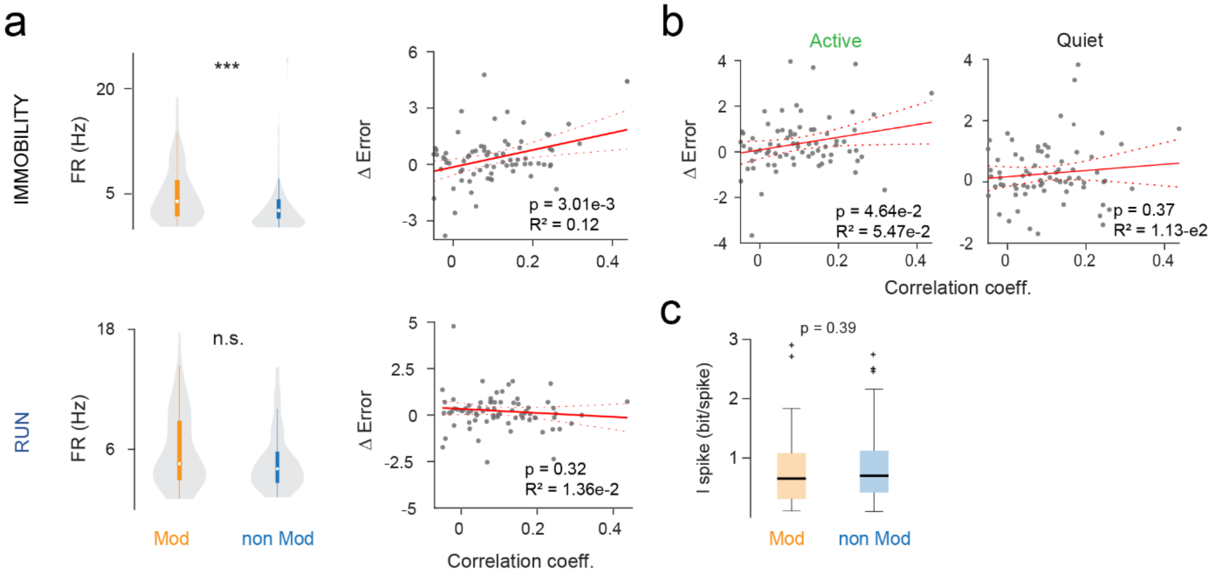

Reviewer_Figure 12. Assessment of the contribution of Behavioral Modulation to spatial coding.
(a) Left, firing rate of behaviorally-modulated and non-modulated cells during immobility (top, p=1.04e-06, Wilcoxon rank-sum test) and running periods (bottom, p=0.07, Wilcoxon rank-sum test). Right, linear models comparing the increase in the error caused by the removal of a cell from the training set (delta error) to its Behavioral Modulation strength (i.e., cross-correlation peak) for immobility (top, R^2=1.17e-1, $F_{(73,71)}$=9.44, p=3.01e-3) and running (bottom, R^2=1.36e-2, $F_{(73,71)}$=0.98, p=0.33). This analysis is now shown in **Fig. 5i.**
(b) Same as in (a), splitting general immobility into active immobility (left, R^2=5.47e-2, $F_{(73,71)}$=4.11, p=4.64e-2) and quiet immobility (right, R^2=1.13e-2, $F_{(73,71)}$=0.81, p=0.37). This analysis is now shown in **Supplementary Fig. 2d.**
(c) Spatial information of modulated and non-modulated cells during running (p=0.39, Wilcoxon rank-sum test). This analysis is now shown in **Supplementary Fig. 6d.**

In essence, the decoding analyses above strengthen three key aspects of our work:

(i) CA1 place cells retain spatial coding during immobility;
(ii) Spatial coding during immobility is most robust during active states and reduced during quiet states, indicative of behavioral-state modulation;
(iii) Behavioral modulation represents a distinctive feature of spatial coding during immobility, whereas this functional distinction is not observed during locomotion.

A4. Sound stimulation controls

Based on prior literature, we considered it unlikely that a sound alone would evoke place cell activity, since previous studies have consistently shown that individual sensory cues are not incorporated into the hippocampal map (as place field activity) unless they are behaviorally relevant or they can serve as a reliable anchoring cue[13,49]. In our experiments, sensory cues were delivered across recordings, with no association to specific belt locations, and were not coupled to the task (i.e., no behavioral contingency). Therefore, it could not serve as a reliable anchoring cue or as a behaviorally relevant stimulus, and we assumed that under these conditions, the sound alone would not affect the rapid emergence of place field activity.
Despite these considerations, to rigorously address the Reviewer's comment, we performed additional control experiments in which only the sound was delivered, without electrical stimulation (i.e., sound-only controls). Specifically, we conducted a subset of juxtacellular recordings (n=20 units) and silicon probe recordings (n=60 units), which were necessary to expand the dataset within the practical constraints of the revision period. As shown in **Reviewer_Fig. 13b**, the results from both datasets were consistent and were therefore pooled in **Reviewer_Fig. 13a**, indicating that the sound alone has no detectable effect on population place cell activity at the stimulus location.

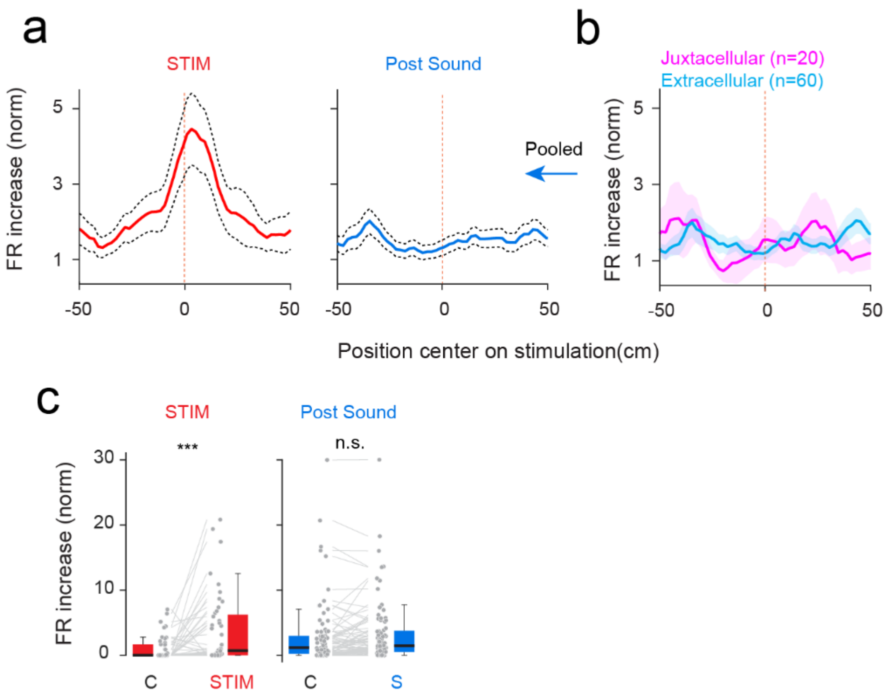

Reviewer_Figure 13. Single-cell juxtacellular stimulation of single CA1 neurons during immobility, and sound-only control.
(a) Normalized average firing rates centered on the juxtacellular stimulus location (left panel, n = 20) and on the sound-only stimulus location (right panel, n = 60). Note the increased firing rate around the stimulus location in the juxtacellular stimulation dataset, which is not observed in the sound-only control dataset. This analysis is now shown in **Fig 3e.**
(b) The "sound-only" dataset in (a) includes n = 20 juxtacellularly recorded cells and n = 60 extracellularly recorded cells, pooled together because both recording methods showed similar results.
(c) Quantification of the average firing rate around the stimulus location for juxtacellularly stimulated cells (n = 44 cells, $p = 4.90 \times 10^{-4}$, Wilcoxon signed-rank test) and for sound-only stimulated cells (n = 80 cells, p = 0.13, Wilcoxon signed-rank test). Error bars indicate SEM. This analysis is now shown in **Fig 3f.**

In **Reviewer_Fig. 13c,** we show that rapid place-field plasticity effects are observed only when neurons are electrically stimulated, as evident both in the population average (**Fig. 3e**) and in the firing rate increase at the stimulus location following juxtacellular stimulation, but not after 'sound-only' stimulation (**Reviewer Fig. 13c, Fig. 3g**). This was further confirmed by a quantification of stimulation effects at the single-cell level, i.e. 11 out of 44 cells (~25%) for juxtacellular stimulation, and 2 out of 80 cells (~2.5%) for sound-only stimulation (p=1.89e-04, Fisher exact test; for details, see section 'Juxtacellular stimulation analysis' of Methods).

A5. Dataset overview

The following section describe the dataset used in the study, providing a overview at the single-unit level, at the recording session-level. A summarized version of this section has been added to the Methods (see section 'Dataset overview').

Single-unit level overview
From a total of 644 CA1 units (n=34 recording sessions from N=5 mice), 520 units were classified as putative pyramidal neurons and 108 units as FS interneurons, based on spike waveform features (see section '*Spike sorting and unit classification*' of Methods). Among FS interneurons, 105 out of 108 units were included in the analysis because of additional firing stability criteria (see section '*Behavioral modulation identification*' of Methods for details).
340 out of 520 putative pyramidal units were classified as place cells (see criteria in '*Place cell analysis*') and formed the basis of the following analysis:

- **'In field' group (n=166 units)** – i.e. place cells with at least one immobility period within the place field - used for analysis related to **Fig. 2** and **Fig. 5**. Of these, **n=81** were classified as behaviorally-modulated, and **n=85** as non-modulated (see **Fig. 5**).
- **'Out of field' group (n=164 units)** – i.e. place cells with at least one immobility period outside the place field - used for analysis related to **Fig. 2**.
- **'In & Out of field' group (n=105 units)** – i.e. place cells with at least one recording inside and outside the place field - used for per-session validation analyses in **Supplementary Fig. 2e**.

For Bayesian decoding analysis, to ensure ratemap stability where decoding is performed – which is an implicit assumption of this analysis (see e.g. [27]) – we applied additional stability criteria on place cell firing, which resulted in only a subset of place cells being included in this analysis: n=180 out of 340 place cells (from 22 sessions) for the analysis shown in **Fig. 2g**; and 73 out of 166 "in field" cells (from 13 sessions) for the impact of behavioral modulation analysis shown in **Fig. 5j** (details in '*Bayesian decoding analysis*' in Methods).
For computing population tuning curves during immobility, we applied additional inclusion criteria (e.g. by restricting the analysis to place cells with single fields, see details in Methods) which resulted in a selection of 280 immobility periods coming from n=131 place cells (displayed in **Fig. 2e**); n=93 immobility periods from n=44 behaviorally-modulated place cells, and n=139 immobility periods from n=57 non-modulated place cells (displayed in **Fig. 5g**) (see details in '*Analysis of place cell firing during awake immobility*' of Methods). We note that these visualizations were intended solely for display purposes, and no analysis was performed on this subset of data.

Session-level overview
Findings were also confirmed with a session-level analysis (n=34 sessions) (see **Supplementary Fig. 2e**). For theta-oscillations related analyses (**Fig. 2b,c, Fig. 4**), 32 out of 34 sessions were included (2 sessions were excluded as a result of a more stringent signal-to-noise criteria to ensure reliable detection of theta oscillations over low-frequency noise; see section 'Theta oscillations segmentation and analysis' of Methods). In the SPW-R related analyses (**Fig. 2b, Supplementary Fig. 3**) 30 out of 34 sessions were included (4 sessions were discarded because of high-frequency artifacts contamination impacting ripple detection, which resulted in the absence of the polarity reversal in the average ripple across the probe shanks; see section '*Sharp-wave ripple detection and analysis*' of Methods).

Juxtacellular stimulation dataset.
The stimulation dataset (**Fig. 3**) included 44 juxtacellularly-stimulated and 80 sound-stimulated putative pyramidal neurons (n=20 juxtacellular, n=60 extracellular). A subset of juxtacellularly recorded cells (n=4) underwent both sound and juxtacellular stimulation sequentially, as illustrated by the representative cell in

Fig. 3c. We had initially opted for this sequential stimulation protocol to address Reviewers' comments on the sound control, but this proved challenging and delayed the revision substantially. Due to the limited duration and stability of juxtacellular recordings, only a few cells underwent both stimulations; therefore, we opted for having separate groups for "juxtacellular stimulation" and "sound-only" controls; see comment 11.2 of Reviewer #1, comment 12 of Reviewer #2, comment 5 of Reviewer #3).

References

1. Mizuseki, K., Diba, K., Pastalkova, E. & Buzsáki, G. Hippocampal CA1 pyramidal cells form functionally distinct sublayers. *Nat Neurosci* **14**, 1174–1181 (2011).
2. Sharif, F., Tayebi, B., Buzsáki, G., Royer, S. & Fernandez-Ruiz, A. Subcircuits of Deep and Superficial CA1 Place Cells Support Efficient Spatial Coding across Heterogeneous Environments. *Neuron* **109**, 363-376.e6 (2021).
3. Pastalkova, E., Itskov, V., Amarasingham, A. & Buzsáki, G. Internally Generated Cell Assembly Sequences in the Rat Hippocampus. *Science* **321**, 1322–1327 (2008).
4. Eichenbaum, H. Time cells in the hippocampus: a new dimension for mapping memories. *Nat Rev Neurosci* **15**, 732–744 (2014).
5. MacDonald, C. J., Lepage, K. Q., Eden, U. T. & Eichenbaum, H. Hippocampal "Time Cells" Bridge the Gap in Memory for Discontiguous Events. *Neuron* **71**, 737–749 (2011).
6. Eichenbaum, H. Memory on time. *Trends in Cognitive Sciences* **17**, 81–88 (2013).
7. Diamond, M. E., von Heimendahl, M., Knutsen, P. M., Kleinfeld, D. & Ahissar, E. 'Where' and 'what' in the whisker sensorimotor system. *Nat Rev Neurosci* **9**, 601–612 (2008).
8. Cheung, J., Maire, P., Kim, J., Sy, J. & Hires, S. A. The Sensorimotor Basis of Whisker-Guided Anteroposterior Object Localization in Head-Fixed Mice. *Current Biology* **29**, 3029-3040.e4 (2019).
9. Bannister, N. J. & Larkman, A. U. Dendritic morphology of CA1 pyramidal neurones from the rat hippocampus: I. Branching patterns. *Journal of Comparative Neurology* **360**, 150–160 (1995).
10. Helton, T. D., Zhao, M., Farris, S. & Dudek, S. M. Diversity of dendritic morphology and entorhinal cortex synaptic effectiveness in mouse CA2 pyramidal neurons. *Hippocampus* **29**, 78–92 (2019).
11. Hodapp, A. *et al.* Dendritic axon origin enables information gating by perisomatic inhibition in pyramidal neurons. *Science* **377**, 1448–1452 (2022).
12. Valero, M. *et al.* Determinants of different deep and superficial CA1 pyramidal cell dynamics during sharp-wave ripples. *Nat Neurosci* **18**, 1281–1290 (2015).
13. Moita, M. A. P., Rosis, S., Zhou, Y., LeDoux, J. E. & Blair, H. T. Hippocampal Place Cells Acquire Location-Specific Responses to the Conditioned Stimulus during Auditory Fear Conditioning. *Neuron* **37**, 485–497 (2003).
14. Clayton, K. K. *et al.* Sound elicits stereotyped facial movements that provide a sensitive index of hearing abilities in mice. *Current Biology* **34**, 1605-1620.e5 (2024).
15. Blanco-Hernández, E., Balsamo, G., Preston-Ferrer, P. & Burgalossi, A. Sensory and behavioral modulation of thalamic head-direction cells. *Nat Neurosci* **27**, 28–33 (2024).
16. Gauthier, J. L. & Tank, D. W. A Dedicated Population for Reward Coding in the Hippocampus. *Neuron* **99**, 179-193.e7 (2018).
17. Mitchinson, B., Martin, C. J., Grant, R. A. & Prescott, T. J. Feedback control in active sensing: rat exploratory whisking is modulated by environmental contact. *Proceedings of the Royal Society B: Biological Sciences* **274**, 1035–1041 (2007).
18. Sofroniew, N. J., Cohen, J. D., Lee, A. K. & Svoboda, K. Natural Whisker-Guided Behavior by Head-Fixed Mice in Tactile Virtual Reality. *J. Neurosci.* **34**, 9537–9550 (2014).
19. Brown, S. T. & Raman, I. M. Sensorimotor Integration and Amplification of Reflexive Whisking by Well-Timed Spiking in the Cerebellar Corticonuclear Circuit. *Neuron* **99**, 564-575.e2 (2018).
20. Collins, L., Francis, J., Emanuel, B. & McCormick, D. A. Cholinergic and noradrenergic axonal activity contains a behavioral-state signal that is coordinated across the dorsal cortex. *eLife* **12**, e81826 (2023).
21. Sharif, F., Tayebi, B., Buzsáki, G., Royer, S. & Fernandez-Ruiz, A. Subcircuits of Deep and Superficial CA1 Place Cells Support Efficient Spatial Coding across Heterogeneous Environments. *Neuron* **109**, 363-376.e6 (2021).
22. Bittner, K. C. *et al.* Conjunctive input processing drives feature selectivity in hippocampal CA1 neurons. *Nat Neurosci* **18**, 1133–1142 (2015).
23. Bittner, K. C., Milstein, A. D., Grienberger, C., Romani, S. & Magee, J. C. Behavioral time scale synaptic plasticity underlies CA1 place fields. *Science* **357**, 1033–1036 (2017).
24. Hodapp, A. *et al.* Dendritic axon origin enables information gating by perisomatic inhibition in pyramidal neurons. *Science* **377**, 1448–1452 (2022).
25. Fenton, A. A. Remapping revisited: how the hippocampus represents different spaces. *Nat. Rev. Neurosci.* **25**, 428–448 (2024).
26. Colgin, L. L., Moser, E. I. & Moser, M.-B. Understanding memory through hippocampal remapping. *Trends in Neurosciences* **31**, 469–477 (2008).

27. Zhang, K., Ginzburg, I., McNaughton, B. L. & Sejnowski, T. J. Interpreting Neuronal Population Activity by Reconstruction: Unified Framework With Application to Hippocampal Place Cells. *Journal of Neurophysiology* **79**, 1017–1044 (1998).
28. Brown, E. N., Frank, L. M., Tang, D., Quirk, M. C. & Wilson, M. A. A Statistical Paradigm for Neural Spike Train Decoding Applied to Position Prediction from Ensemble Firing Patterns of Rat Hippocampal Place Cells. *J. Neurosci.* **18**, 7411–7425 (1998).
29. Kloosterman, F., Layton, S. P., Chen, Z. & Wilson, M. A. Bayesian decoding using unsorted spikes in the rat hippocampus. *Journal of Neurophysiology* **111**, 217–227 (2014).
30. Liu, A. A. *et al.* A consensus statement on detection of hippocampal sharp wave ripples and differentiation from other fast oscillations. *Nat Commun* **13**, 6000 (2022).
31. Buzsáki, G. Hippocampal sharp wave-ripple: A cognitive biomarker for episodic memory and planning. *Hippocampus* **25**, 1073–1188 (2015).
32. Hulse, B. K., Lubenov, E. V. & Siapas, A. G. Brain State Dependence of Hippocampal Subthreshold Activity in Awake Mice. *Cell Reports* **18**, 136–147 (2017).
33. Bagur, S. *et al.* Harnessing olfactory bulb oscillations to perform fully brain-based sleep-scoring and real-time monitoring of anaesthesia depth. *PLOS Biology* **16**, e2005458 (2018).
34. Nestvogel, D. B. & McCormick, D. A. Visual thalamocortical mechanisms of waking state-dependent activity and alpha oscillations. *Neuron* **110**, 120-138.e4 (2022).
35. Markus, E. J., Barnes, C. A., McNaughton, B. L., Gladden, V. L. & Skaggs, W. E. Spatial information content and reliability of hippocampal CA1 neurons: Effects of visual input. *Hippocampus* **4**, 410–421 (1994).
36. Skaggs, W. E., McNaughton, B. L., Wilson, M. A. & Barnes, C. A. Theta phase precession in hippocampal neuronal populations and the compression of temporal sequences. *Hippocampus* **6**, 149–172 (1996).
37. Wu, X. & Foster, D. J. Hippocampal Replay Captures the Unique Topological Structure of a Novel Environment. *J. Neurosci.* **34**, 6459–6469 (2014).
38. Davidson, T. J., Kloosterman, F. & Wilson, M. A. Hippocampal Replay of Extended Experience. *Neuron* **63**, 497–507 (2009).
39. Ambrose, R. E., Pfeiffer, B. E. & Foster, D. J. Reverse Replay of Hippocampal Place Cells Is Uniquely Modulated by Changing Reward. *Neuron* **91**, 1124–1136 (2016).
40. Singer, A. C. & Frank, L. M. Rewarded Outcomes Enhance Reactivation of Experience in the Hippocampus. *Neuron* **64**, 910–921 (2009).
41. Houweling, A. R. & Brecht, M. Behavioural report of single neuron stimulation in somatosensory cortex. *Nature* **451**, 65–68 (2008).
42. Diamantaki, M. *et al.* Manipulating Hippocampal Place Cell Activity by Single-Cell Stimulation in Freely Moving Mice. *Cell Reports* **23**, 32–38 (2018).
43. Dunn, S. L. S., Town, S. M., Bizley, J. K. & Bendor, D. Behaviourally modulated hippocampal theta oscillations in the ferret persist during both locomotion and immobility. *Nat Commun* **13**, 5905 (2022).
44. Lasztóczi, B. & Klausberger, T. Hippocampal Place Cells Couple to Three Different Gamma Oscillations during Place Field Traversal. *Neuron* **91**, 34–40 (2016).
45. Malezieux, M., Kees, A. L. & Mulle, C. Theta Oscillations Coincide with Sustained Hyperpolarization in CA3 Pyramidal Cells, Underlying Decreased Firing. *Cell Reports* **32**, 107868 (2020).
46. Chiang, F.-K., Wallis, J. D. & Rich, E. L. Cognitive strategies shift information from single neurons to populations in prefrontal cortex. *Neuron* **110**, 709-721.e4 (2022).
47. Narayanan, N. S., Kimchi, E. Y. & Laubach, M. Redundancy and Synergy of Neuronal Ensembles in Motor Cortex. *J. Neurosci.* **25**, 4207–4216 (2005).
48. *Methods for Neural Ensemble Recordings*. (CRC Press/Taylor & Francis, Boca Raton (FL), 2008).
49. Shan, K. Q., Lubenov, E. V., Papadopoulou, M. & Siapas, A. G. Spatial tuning and brain state account for dorsal hippocampal CA1 activity in a non-spatial learning task. *eLife* **5**, e14321 (2016).

Dear Editor, dear Reviewers,

We thank the Reviewers for their helpful and constructive feedback.

In this submission, we have clarified the few remaining minor points raised by the Reviewers and made corresponding adjustments in the manuscript text where appropriate, improving both clarity and presentation. All points have been addressed in detail in our response letter.

We therefore submit the revised manuscript, which we hope will now be acceptable for publication in *Nature Communications*.

Andrea Burgalossi
(on behalf of all authors)

Reviewer #1

I have read the response to reviewer 1 and the common response to all reviewers and have also browsed through the paper and the response to all reviewers, and think the paper has substantially improved. I appreciate the well thought of and well written rebuttal, which has taken seriously all the comments written by the reviewers.
COMMENT: We thank the reviewer for their positive feedback. We also believe their insights greatly improved the manuscript.

I have some minor comments left, that may be fixed towards publication by refining the Discussion:
1. The fact that the second map was not revealed in your experiment does not mean it does not exist. Specifically, maps could flip between whisking and non-whisking states, such that the hippocampus toggles between two different maps belonging to two different contexts. The pre-post correlation does not answer this because it could be that there was one map before the stop, similar to the map after the stop, with a different map in-between. Maps could actually even flip quickly at a speed of one map per theta cycle (Jezek, Karel, Espen J. Henriksen, Alessandro Treves, Edvard I. Moser, and May-Britt Moser. "Theta-paced flickering between place-cell maps in the hippocampus." Nature 478, no. 7368 (2011): 246-249). The main point is that I do not expect the non-modulated group to be anatomically distinct from the modulated group. Things in CA1 are more dynamic than this.
COMMENT: We thank the reviewer for the comment. While we acknowledge that flickering between hippocampal maps can occur, it is important to note that our experimental design involved a single, consistent environment (the treadmill belt). This differs fundamentally from the paradigm used by Jezek et al. (2011), where flickering was triggered by the competition between two representations of two different contexts. Furthermore, our decoding analysis (**Fig. 2g**) shows that place coding is partially preserved during immobility, suggesting that the spatial map remains, on average, stable even in the absence of locomotion.
Nevertheless, we agree with the Reviewer that transient 'flickering' at the theta-cycle level remains a possibility and could contribute to the degradation of the spatial representation observed during immobility in our conditions.
We agree the anatomical distinction between the two groups is not strongly supported by our data and further studies should clarify it.
CHANGE: We have acknowledged this point in the Methods (line 137-141, page 3).

2. Reviewer figure 3 - Precession could be clearly seen also during immobility (although panel b is noisier). All you have to do is spatially smooth panel b and some precession will emerge.
COMMENT: We thank the reviewer for this thoughtful comment. The reviewer is correct in noting that, after spatial smoothing, panel B may suggest the presence of phase precession during immobility. However, this population-averaged representation does not capture the substantial variability we observe at the level of individual cells. In our current dataset, we do not observe a consistent or robust pattern of phase precession across neurons during immobility.
Given the potential conceptual impact of demonstrating phase precession in the absence of movement, we believe that the present data are still too preliminary to support a strong conclusion. For this reason, we prefer not to include this analysis in the current manuscript. Instead, we plan to address this question more thoroughly in a follow-up study with additional experiments and a more comprehensive analysis.
CHANGE: none.

These points are not essential and do not preclude publication, as the paper is really nice and all essential points have been answered.
COMMENT: We thank the reviewer, and we are glad they appreciated our work.

Reviewer #2
First, I would like to commend the authors. They put in a lot of work to improve their manuscript and it is much more polished now. The revisions have clarified which results are robust and which do not stand up to more rigorous statistical assessment. I have a few remaining issues, and pending the resolution of these issues, I support publication of the paper.
COMMENT: We thank the reviewer for the positive feedback. Thanks to their advice, the manuscript greatly improved.

1. The authors argue in their rebuttal:
"*The Reviewer raised a concern related to the computation of statistics using cells as individual units. While we acknowledge that this is, in principle, a valid concern, it is standard practice in hippocampal research to treat place cells as independent units (see, e.g., 3, 21–24). This assumption is supported by extensive evidence showing that individual cells map space independently of one another (see e.g. remapping, and the uncoordinated behavior of cell-pairs relative to one another; for a review, see 25,26). Moreover, the standard Bayesian decoders widely used in the hippocampal literature also rely on the assumption of independence of place cell firing given the animal's position 27–29.*" Here, I don't think their reasoning is good, but only have a small requested change.
First, I agree with the authors that in the hippocampal literature, treating co-recorded place cells as independent samples is common. So, I can understand why they do it. Unfortunately, common practice doesn't always mean best practice, especially in different and new experimental conditions. I encourage the authors to inform themselves about some of the common pseudo-replication errors throughout neuroscience-related research fields and their implications (see for example "Better statistical reporting does not lead to statistical rigour: lessons from two decades of pseudo-replication in mouse-model studies of neurological disorders. Eleftheriou et al. 2025).
Second, they should be careful about what exactly is independent and whether that independence is the right kind of independence for their statistical tests. Even if "…individual cells map space independently of one another…", place cells are of course not only coding space (as the authors show in their manuscript, they encode behavioral state variables too). Moreover, network interactions explain a substantial amount of population activity in CA1 (see Collective Behavior of Place and Non-place Neurons in the Hippocampal Network, Meshulam et al. Neuron 2017), meaning even if place fields are independent, the general spiking activity of CA1 neurons is not. Many of the statistical tests in the manuscript are not about spatial coding alone, but rather they pertain to behavioral-modulation of spatial coding during immobility (Active vs Quiet). About half the cells are behaviorally modulated in a place-specific manner, but that does not mean that behavioral modulation is also independent in co-recorded cells (even if it is only visible at uncorrelated place field centers). Their results suggest that inputs that drive behavioral modulation target some cells more than others (not all cells display it), and depending on the session, we might have more or less cells more strongly targeted by these inputs. It is also possible that the inputs driving behavioral modulation themselves are more or less active in different sessions. Both of these possibilities mean that we might have within session correlations of behavioral modulation that are seen only at place field centers. Within session correlations would imply a lack of independence. Treating cells individually masks this co-variability, which could potentially lead to false positive ridden statistics (see again Eleftheriou et al. 2025 Fig. 3B-D).
For these two reasons, 1) the common practice cannot be blindly applied in new situations, and 2) when looking at behavioral modulations of spatial coding (Active vs Quiet, not spatial coding alone), independence is not established. I reiterate the need for session-based statistics even if it does not change the conclusions (great news if it does not). The authors do not need to move anything, and they can leave the figures, just refer the reader to session-based stats (when available and applicable, even if they are in supp figs) explicitly in the main text.
COMMENT: We fully agree with the Reviewer that, although treating co-recorded place cells as independent samples is common practice in the hippocampal literature, such assumptions should not be applied uncritically. We appreciate the Reviewer's emphasis on the need to carefully evaluate the relevant form of statistical independence.
Importantly, we would like to clarify that we have already conducted extensive session-level analyses wherever this was feasible and appropriate, and that the main conclusions of the manuscript are consistently supported at the session level. Specifically, all key findings have been confirmed by session-level analysis, including e.g. **Fig. 1d**, **Fig. 2b-d**, **Supplementary Fig. 2c,e**, and **Fig. 4** (for **Fig. 5**, see below).

We understand the concern of the Reviewer that, if firing rate of place cells covary with fluctuating arousal levels, cells might not satisfy the independence requirement of statistical tests (i.e., resulting in correlated variability). For this reason, we conducted additional session level analyses related to **Fig. 5h** and **Fig. 5i**, (see **Reviewer_Figure 1**) the results of which are fully in line with our previous analysis and interpretation. We refer to this additional analysis in the revised manuscript.

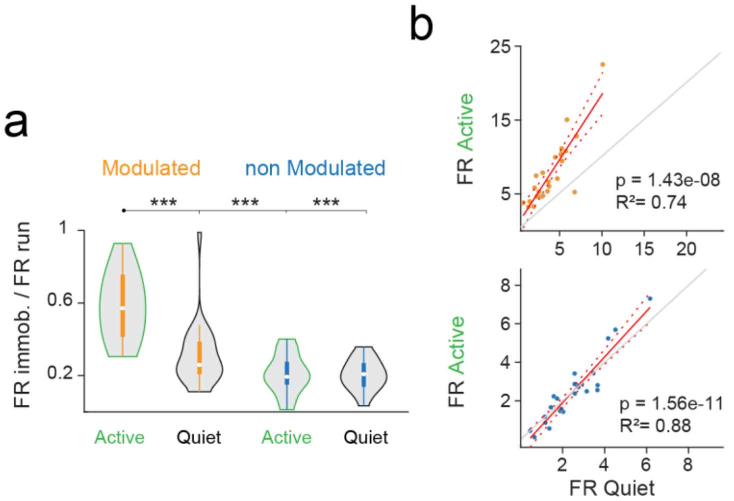

Reviewer_Figure 1. New session level analyses related to Fig. 5.
(a) Session level-analysis of Fig. 5h. In-field firing rates during immobility (normalized to in-field rates during running) of behaviorally-modulated (orange) and non-modulated (blue) place cells, during active (green) and quiet (black) immobility. p=3.01e-11, Kruskal-Wallis test, post-hoc test: *** p<0.001, n=26 sessions for modulated cells, n=24 sessions for non-modulated cells.
(b) Session level-analysis of Fig. 5i. Firing rate comparison between quiet and active immobility for behaviorally-modulated cells (top, test of slope $\beta \neq 1$, p = 1.40e-3; linear model, R^2=0.74, $F(26,24)$=69.90, p=1.43e-08) and non-modulated cells (bottom, test of slope $\beta \neq 1$, p = 0.06; linear model, R^2=0.88, $F(24,22)$=159.00, p=1.56e-11).

Taken together, we believe that the session-level analyses included in the manuscript and supplementary material adequately address concerns about statistical independence. We are grateful to the Reviewer for this comment, as it has led to a clearer and more rigorous presentation of our analyses.
CHANGE: In the revised results (lines 242, 248, page 9), we specified that the analysis related to **Fig. 5h** and **Fig. 5i** was verified at the session-level.

2. The authors use the concept of "excitability" 4 times in the manuscript (lines 133, 218, 220, and 298, but also many times in the rebuttal letter) to explain why firing rates of certain neurons are higher than other neurons. For me, changes in excitability is a borrowed concept from intracellular physiology that cannot be concluded here. In intracellular physiology, cells that are more "excitable" fire more readily for a fixed level of direct current injection. Here, in extracellular conditions where we only record spikes of large neuronal populations with no control over their synaptic input, we cannot know if the cells are receiving the same input levels to then conclude that one is more excitable than another. Some cells might receive inputs that other cells do not, so it might not be excitability, it could just be different signals that are integrated. I suggest they reword these 4 sentences to avoid using misleading or speculative language.
CHANGE: We agree with the reviewer that in systems neuroscience, the term “excitability” has been used more broadly and somewhat loosely to refer to observed firing rates or the propensity of a neuron to spike. We have thus revised the manuscript to remove the term ‘excitability’ in the mentioned lines, replacing it with precise terminology.

3. I still think some of the stats exhibited in Fig. 5h are circular, despite down-sampling or shuffling that was performed in the rebuttal. The authors separate cells into behaviorally modulated and non-modulated using a correlation-based (cross-corr peak between spiking activity and whisker motion during immobility) modulation index statistical test. This identifies cells that fire more during Active immobility. Then, they perform a second round of statistical tests in Fig. 5h on these cells to assess whether modulated vs non-modulated cells have different firing rates between active and quiet immobility. Stats between modulated and non-modulated populations are contrived by the classification they make in the first modulation index statistical test. Why this is (see atatached drawing for graphical description) … If I had any distribution across cells (firing rates or correlations with whisker movements here will be very similarly distributed), separated its left tail and right tail based on a first statistical assessment (see Supp Fig. 6a), then performed a second statistical test on the left tail vs right tail, it passes this second statistical test simply because I classified the cells based on the first one. The authors should remove stats that echo prior tests (any stat bars that compare modulated vs non-modulated in this case). If they still want to show and quantify the firing rate modulations WITHIN the modulated or non-modulated groups, that helps to understand the depth of the active vs passive modulation within the cell classes (only 2 stat comparison bars, a significant one for the modulated side and a non-

significant one for the non-modulated), but comparisons between the groups are contrived. I think they should also show here that run firing rates at field center are indistinguishable between modulated and non-modulated cells to be more in line with their rebuttal argument ("… specifically, modulated and non-modulated cells exhibit similar excitability during running (i.e., comparable firing rates), despite the different rates during immobility "). They show something about this in 5f but only for corr coeff, so it would be simpler to see just run firing rates at field centers for non-modulated vs modulated, which they suggest are similar but I do not see it anywhere in the figures (or supp figures, apologies if I missed it).

COMMENT: We thank the Reviewer for the comment. We understand the concern that if classification were dependent on firing rate magnitude, subsequent quantification of that same rate would be circular. However, we would like to clarify the following:

1. The Reviewer states that our classification analysis *"…identifies cells that fire more during active immobility*". We apologize for this lack of clarity. In fact, our classification is based on a *normalized* cross-correlation, which measures how much two signals covary together – in this case, how well the spikes follow the whisker movement – rather than the number of spikes. Cells with low firing rates can have high cross-correlation peaks if spikes accumulate at the onsets of whisker-pad motion. On the other hand, cells with high firing rates whose spikes do not precisely align at the onset of whisker-pad motion will have a low cross-correlation peak.
2. Having a high cross-correlation peak does not necessarily entail high firing rates during active immobility compared to quiet immobility. For instance, the same average firing rate in active and quiet immobility can result in a high cross-correlation peak if spikes systematically cluster at the onsets of whisker-pad motion.

In spite of the above considerations, we acknowledge that firing rates alone can contribute to correlation quantifications and therefore we included in the first version of our study two ways to control for this, namely: down-sampling (directly addressing the FR influence) and shuffling (addressing the temporal coordination), to demonstrate that behavioral modulation is not a byproduct of higher firing rates in modulated cells (**Supplementary Fig. S6b**).

To further address the Reviewer's concern in this second round, we performed an additional analysis using a "rate-matched classification." First, we downsampled the spikes of modulated cells to match the firing rates of non-modulated cells. We then re-classified the cells as modulated or non-modulated by computing the cross-correlation and modulation index based on shuffling, as described previously. This analysis revealed that even after downsampling, the vast majority of cells (~95%; 77 out of 81) remained significantly modulated. These results indicate that behavioral modulation is driven primarily by the precise temporal clustering of spikes around whisker-pad motion onsets, rather than by differences in overall firing rates. We have included the results of this additional analysis in the revised manuscript.

In summary, for the reasons outlined above, we believe that the comparison in **Fig. 5h** remains valuable, as it serves as a functional characterization of the two groups.

Regarding the comparison of firing rates between modulated and non-modulated cells during running, we had removed this plot from the figure in response to the reviewer's in first revision comment. The corresponding comparison is however provided within the text (line 223, page 8).

CHANGE: The "rate-matched classification" analysis is now referred to in the revised results (page 9 lines 231-232).

4. Many heatmap figures have no color bar (3c at the right, 3d left, 4b, 4c, etc). Please add color bars to all heatmaps and carefully review figures for other missing labels and details.

CHANGE: We thank the Reviewer for spotting this, we have added the color bars and checked all the figures.

Reviewer #3

The authors provided a highly detailed and comprehensive rebuttal to the comments I and the other reviewers provided: I was impressed by the scope and depth of the new analysis and experiments presented in the rebuttal. Specifically, the authors strengthened 1) analysis of LFP signals (specifically theta power and frequency) during arousal states, 2) the cognitive representation of location during active immobility, by implementing bayesian decoding, and 3) a sound-only control during the place field induction experiments. I think these changes have made the story much more comprehensive, and strengthened the claims that behavioral modulation of hippocampal activity sharpens spatial representations, even when the mouse is stationary. In light of these improvements, I have some minor comments. Should these be addressed I would be happy to see this work published:
COMMENT: We thank the reviewer for the positive assessment of our work.

Minor Comments:
1. Figure 2b,c. What level were statistical tests over? n=32/34 sessions according to the legend of b, but the p-values/error bars seem quite small given that n. Are the tests calculated over all whisking events or average zscore of the change in LFP power per session?
COMMENT: We apologize for the lack of clarity. As specified in the legend of **Fig. 2b**, error bars indicate the standard error of the mean across sessions (n=32 sessions). We think that the combination between the use of the SEM and the narrow layout of the panel leads to error bars to appear narrow. The significance tests of **Fig. 2c** (Wilcoxon signed-rank tests) were conducted over the z-scored values (normalized to baseline, from -2 to 0 s) for each session in a specific LFP band (i.e., theta, delta or gamma).
CHANGE: The above points have been clarified in the corresponding figure legend (**Fig. 2c**).

2. Line 90: "sharp-wave ripples predominantly occurred during 'quiet' immobility." This statement suggests that ripples did not occur during 'active' immobility, but is unclear what the true ripple rate is during the active state because it is a z-score (ie. despite decreasing relative to the quiet state, the ripple rate could be quite high for all we know). It would be more transparent to show the raw ripple rates or to change the interpretation accordingly ("there is a decrease in the ripple rate after a transition to 'active' immobility"). If there truly are no ripples in the active immobile state, this is a very interesting finding and should be reported clearly.
COMMENT: We thank the Reviewer for this comment. To address the concern, we quantified ripple rates during transitions from quiet to active immobility without applying normalization. Ripple rates decreased from 0.18 ± 0.16 Hz during quiet immobility to 0.05 ± 0.10 Hz during active immobility, corresponding to an approximate 70% reduction ($p = 2.60 \times 10^{-6}$, Wilcoxon signed-rank test). Based on these results, we believe that describing ripples as "predominantly occurring during quiet immobility" is both accurate and a conservative characterization.
CHANGE: The above ripple rates have been added to the manuscript (line 91-92, page 5).

3. Figure 2g. I appreciate the new decoding analysis, however, the presentation of decoding error as a z-score relative to shuffled error is somewhat confusing. I am curious what the true error of the decoder is (traditionally reported as average error in cm), which, as far as I can tell, is not plotted or reported anywhere. Maybe a more transparent way to present this would be to show the confusion matrix and shuffle distribution Supp Fig 2b in main figure 2, and to instead label the x axis of the shuffle distribution as the true decoder error in cm. Then indicate the z-score of the true error relative to the shuffle. I think this would make the exact procedure/change in error plotted in 2g much more clear.
COMMENT: We thank the Reviewer for the feedback regarding the decoding analysis. While decoding errors are often reported in absolute units (such as centimeters) this metric is not appropriate for the current study. Unlike standard decoding, where training data typically spans the entire environment with consecutive, regularly spaced positions, our "space-clamping" approach restricts the training data to discrete immobility locations, which are neither contiguous nor regularly spaced. As a result, the decoder is constrained to predict only these specific locations, causing absolute error values to appear artificially large, and making them difficult to compare with values reported in the literature. Additionally, the number and spatial distribution of immobility locations vary across sessions, which determines a different chance-level error for each session. For these reasons, normalizing the decoding error relative to a shuffled distribution provides a more meaningful and unbiased comparison across conditions, allowing us to quantify decoding performance while accounting

for differences in location sampling. We thus opted to report in the manuscript only the normalized error and not the absolute error, to avoid misleading comparisons to standard decoding performances.
Regarding the layout, we thank the reviewer for the suggestion. As recommended, we have now labeled the shuffle distribution in terms of true decoder error in centimeters. We also attempted to incorporate this panel into main **Figure 2,** but found that it made the figure overly busy and reduced clarity. For this reason, we have retained these elements in **Supplementary Figure 2b**, ensuring the results remain fully accessible while keeping the main figure clear and readable.
CHANGE: We acknowledged this point in the Methods (lines 337-339, page 7-8) and labeled the shuffle distribution in centimeters in **Supplementary Fig. 2b**.

4. Figure 3. The sound-only control is a nice addition, but the general role of this finding in relation to the other results presented here is still unclear to me. While I agree with the author's statement: "This evidence … indicates that place field plasticity mechanisms can be engaged even in the absence of locomotion", this finding would be much more noteworthy in comparison to a similar induction experiment during "quiet" immobility (ie. in a closed-loop fashion when whisking did not occur). To me this result only demonstrates that the place field induction protocol is effective: I am not surprised that it works whether the mouse is running (as in Bittner 2015) or awake and immobile (here). Given unlimited experimental time and a closed loop system, if the authors were to use the same induction protocol during running, "active" immobility, and "inactive" immobility and found it equally likely to induce a place field in all three conditions, it would simply demonstrate the induction protocol works regardless of behavioral state, and the contribution of this finding becomes somewhat moot given the story of the paper. Without these other conditions, it is impossible to say whether the induction protocol "just works" or if there is something special about active arousal states. I don't think there is anything more to address here if the authors cannot do the same experiment during "quiet" immobility, but this result doesn't say anything about how arousal affects place cell formation and therefore feels out of place.
COMMENT: We thank the Reviewer for their feedback and fully agree with their assessment. As the Reviewer correctly points out, without a direct comparison to "quiet" immobility, we cannot determine whether the induction protocol works generally or whether there is something specific about active arousal states. Testing this question is non-trivial, as it would require closed-loop stimulation and within-subject comparisons across behavioral states, involving a substantial number of cells and animals. We plan to address this question in future studies specifically designed to tackle this brain-state dependency.
Nevertheless, we believe that demonstrating place field induction during immobility is a non-trivial finding in its own right. It is not obvious that a place cell would form during immobility simply by driving a given cell to fire, and to our knowledge, this has not been previously described. Our results thus provide valuable evidence that locomotion is not a necessary condition for place cell formation and that hippocampal circuits can incorporate new spatial information even during immobility.
In the revised manuscript, we explicitly acknowledge that state-dependent differences between active and quiet immobility remain an open question. We have added a statement in the Discussion noting that distinguishing between these states is an important direction for future research.
CHANGE: We added a future research statement to the Discussion (line 375-377, page 14).

5. Figure 4a. Line 159: "theta oscillations during immobility showed a larger variability in frequency". Based on the data presented this is not clear to me, the frequency distributions on the top of 4a seem quite similar to me… if anything there looks like a frequency shift rather than change in variability.
CHANGE: We apologize to the Reviewer for the lack of clarity. In the revised manuscript we clarified this point conducting new statistical tests, which showed indeed that, compared to running, theta oscillations during immobility showed a largely overlapping frequency distribution with a small, but significant reduction in frequency (immobility, 7.31 ± 0.80 Hz; run, 7.44 ± 1.10 Hz; $p = 4.55 \times 10^{-25}$, Wilcoxon rank-sum test). We thus rephrased the sentence accordingly in the revised results (see lines 161-165, page 7).

6. In several places in the text here, "theta/delta ratio" is referred to as "theta power" (line 160, 175). I am not sure if this is common convention, but seems somewhat misleading?
CHANGE: We thank the Reviewer for pointing out this apparent inconsistency. Although in our data (**Fig. 2b,c**) an increase in theta/delta ratio reflects, on average, an increase in theta power and a decrease in delta power, we agree that 'theta power' and 'theta/delta ratio' are distinct measures and that these terms cannot be

used interchangeably. We have thus revised the text, substituting 'theta power' with 'theta/delta ratio' where appropriate.

7. Figure 5g. Line 234: "Spatial modulation was predominantly preserved in behaviorally-modulated place cells during active immobility." I'm not sure what this sentence means, both modulated and non-modulated cells appear to have spatial "modulation" (ie. are spatially tuned) during active or quiet immobility. Did you mean to say something like "spatial representations were primarily modulated during 'active' immobility" or "modulation of spatial tuning was predominantly observed in behaviorally-modulated cells during 'active' immobility"?
CHANGE: We apologize to the reviewer for the lack of clarity. We indeed intended to convey the message of the second sentence. We rephrased it accordingly.

8. Typos. Line 77: "while during locomotion" isn't grammatically correct
Line 223: "downsamling"
Line 313: grammar issue "heterogeneity at the … properties", maybe you meant "level"?
CHANGE: We thank the reviewer for spotting these mistakes. We corrected them.

REVIEWER'S COMMENTS

Reviewer #2

I thank the authors for the last revisions. I still have a lingering problem, but I think despite that, the paper is ready for publication because all I am asking them to do is remove two stats tests.
However, I will still try one more time to explain because I don't think the authors have accepted or addressed one comment. Down-sampling is not fixing the problem, nor is firing rate adjustment.
It is a logical problem. So I will try one last time to make it clear why they should remove the stats test bars between the 1st and 3rd row and the 2nd and 4th row in Fig 5h. The comparison between rows 1 and 2 is valid (modulated cells FR ratio in active vs passive state), as is the comparison between rows 3 and 4 (non-modulated cells FR ratio in active vs passive). These quantify the firing rate modulation depth that is depicted also in Fig 5g.
Now, to see what I have a problem with, they should make a scatter plot, at first of all cells with Behavioral Modulation index (BMI) on the x-axis and the firing rate during "active" immobility divided by the firing rate during running (FRactiveimmob / FRrunning) on the y-axis (this is what they quantify on the x-axis in Figure 5h). Modulated cells (determined by their BMI stats test) will be skewed towards the upper right quadrant of this plot. They have higher modulation indexes (pushing them to the right on the x-axis), and higher FRactiveimmob / FRrunning ratios (pushing them up on the y-axis). If this is wrong, I apologize for dwelling on it too long, but I don't see how it can be different. They can now color the modulated cells orange.
The remaining non-modulated cells should not be skewed and be more centered on this plot. They can leave them blue. So, there should be two reasonably separable clusters of dots on the plot in orange and blue (maybe some small overlap because some cells are noisier than others), with orange dots skewed up and to the right. This separation should be apparent on both axes, x- and y-, which indicates these values are correlated. This correlation between BMI and FRratio is a problem because it is definitional, I don't see how (based on their definitions and FR normalization to running) a cell that has relatively higher firing rates during whisking (normalized to running) would not also have a high whisking cross-correlation peak compared to shuffle. For me, the two measures are definitionally related to each other. This is why we cannot classify cells into two groups based on the x-axis stats and then perform a second stats test on the two group averages based on y-axis stats. A neuron that has higher cross-correlations with whisking, will also have higher Firing rates during whisking normalized to run. I cannot do better in explaining this, and I urge the authors to not perform redundant statistics. Removing these tests changing nothing regarding their conclusions, it simply doesn't flood the reader with unnecessary tests.

COMMENT & CHANGE: We thank the reviewer for the comment. We acknowledge the Reviewer's point, and we resolved the issue as suggested. Specifically, we removed the statistical comparisons requested by the Reviewer and, accordingly, (i) revised the text (at line 240) and (ii) updated Fig. 5h, where we have replaced the Kruskal-Wallis test with two Wilcoxon signed-rank tests.

The rest is great for me and I thank the authors for their patience and perseverance.
COMMENT: We thank the Reviewer for the positive assessment of our work.

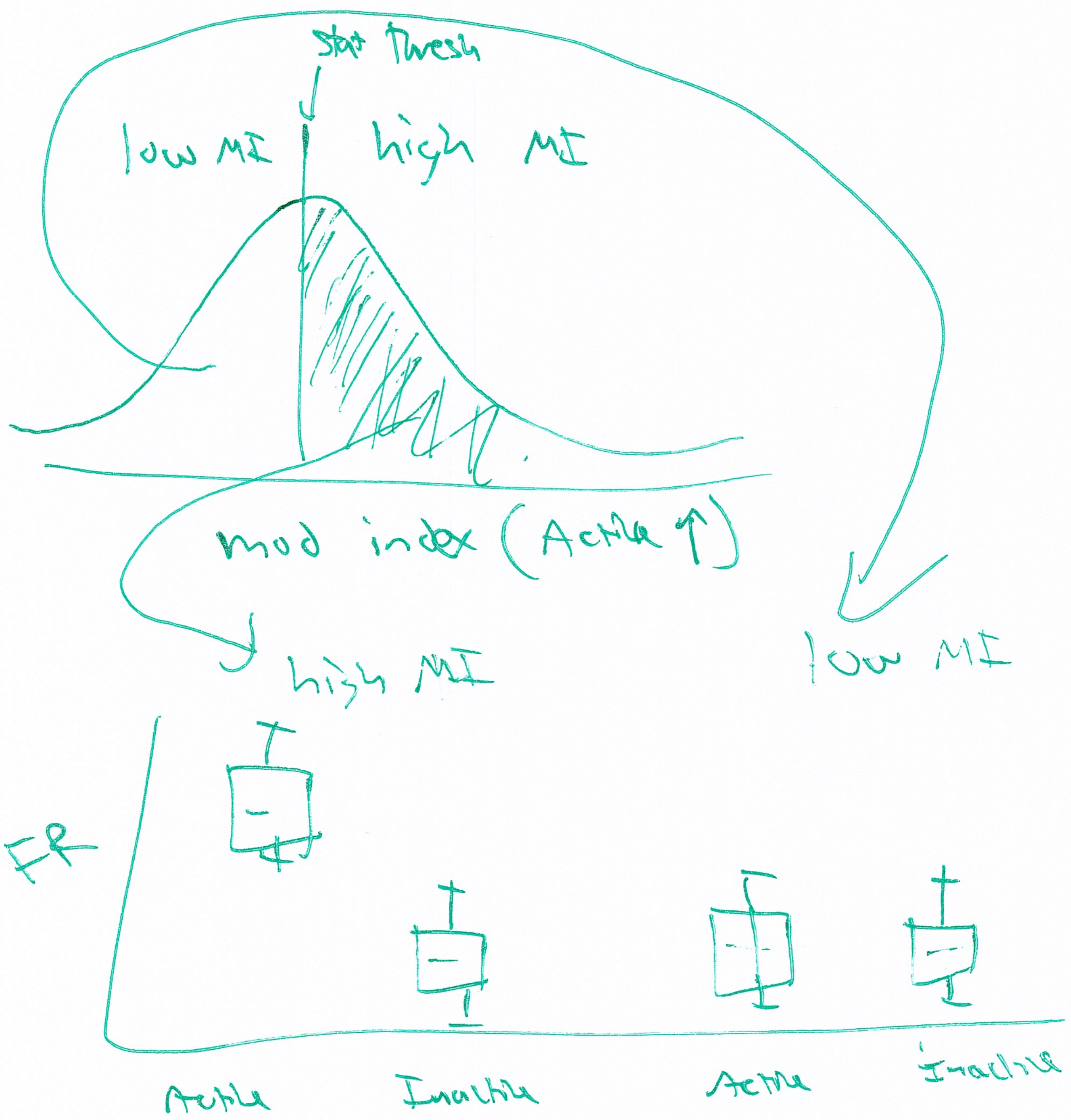
Stat Thresh
low MI
high MI
mod index (Active ↑)
high MI
low MI
FR
Active
Inactive
Active
Inactive
